# Designing Impact Resistance and Robustness into Slippery Lubricant Infused Porous Surfaces

**DOI:** 10.1002/adma.202409818

**Published:** 2024-10-29

**Authors:** Vikramjeet Singh, Jianhui Zhang, Priya Mandal, Dingyu Hou, Ioannis Papakonstantinou, Manish K. Tiwari

**Affiliations:** ^1^ Nanoengineered Systems Laboratory UCL Mechanical Engineering University College London London WC1E 7JE UK; ^2^ Wellcome/EPSRC Centre for Interventional and Surgical Sciences University College London London W1W 7TS UK; ^3^ Photonic Innovations Lab Department of Electronic & Electrical Engineering University College London Torrington Place London WC1E 7JE UK

**Keywords:** biofouling, impact resistance, lubricant retention, metal‐organic framework (MOF), SLIPS

## Abstract

Slippery lubricant infused porous surfaces (SLIPS) have the potential to address daunting challenges such as undesirable surface fouling/biofouling, icing, etc. However, the depletion of lubricants hampers their practical utility. As a solution, here a rational strategy is introduced that operates synergistically in three parts. First, ultra‐high capillary pressure is exploited from the reticular structure of metal‐organic frameworks (MOFs) with sub‐nanometer pores. Second, the need for geometric compatibility is demonstrated; the lubricant chain diameter must be smaller than the MOF pore to enable lubricant chain intercalation. Lastly, the MOF pore chemistry is tailored to achieve a strong intermolecular interaction for any given MOF/lubricant combination. The strategy is investigated through experiments and quantum/molecular simulations, which show that the approach helps lock the intercalated lubricant chains inside the MOF pores and forms a non‐conventional supramolecular structure. The resulting SLIPS (including those with fluorine‐free chemistry) not only show typical low wetting hysteresis, friction, and ice adhesion but are uniquely resistant to more stringent tests such as continuous dripping and sliding of water droplets (up to 50 hrs), repeated impacts of high‐speed water jets (liquid impact Weber number > 4 × 10^4^) and prevent bacterial biofilm formation even in dynamic flow conditions. The findings may widen the practical applications of SLIPS.

## Graphical abstract

Slippery lubricant infused porous surfaces (SLIPS) are presented which seek to address the lubricant drainage issue that impairs such surfaces. The strong lubricant retention achieved enables the SLIPS to maintain their slipperiness under high‐speed liquid impact and other dynamic/flow conditions. These findings should widen the practical applications of SLIPS.

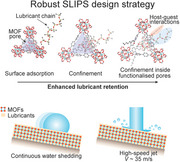

## 1 Introduction

*Nepenthes* pitcher plant‐inspired slippery lubricant‐infused porous surfaces (SLIPS) have been widely researched due to their tremendous potential in solving real‐life problems such as undesirable ice formation on surfaces, fouling, biofouling, and drag in water treatment systems and on underwater vehicles.^[^ ^1^, ^2^, ^3^, ^4^ ^]^ SLIPS present a smooth, defect‐free interface and can be designed to repel all immiscible liquids,^[^ ^5^, ^6^, ^7^ ^]^ with minimal wetting hysteresis. However, with use, the lubricant depletes from the surface over time, leading to the failure of its slippery behavior.^[^ ^8^, ^9^, ^10^ ^]^ Strategies such as holding oil inside a polymer film or using capillary forces from surface nanopores have only had limited success. Durability challenges, especially under dynamic and flow environments, remain unsolved.^[^ ^11^, ^12^ ^]^

Various strategies to hold lubricants have been exploited to overcome the lubricant depletion issue. Efforts have been made to 1) identify the suitable combination of hierarchical textures, materials, and lubricants;^[^ ^13^, ^14^ ^]^ 2) to achieve lubricant replenishment through surface energy gradients, external stimuli, and use of reservoirs;^[^ ^15^, ^16^ ^]^ and 3) to entrap the lubricant in small surface pores via capillary forces^[^ ^8^, ^17^ ^]^ and covalent immobilization.^[^ ^18^, ^19^ ^]^ For example, Laney et al. used nanoscale surface texture to increase the depletion time^[^ ^17^ ^]^ and few studies have used NH_2_‐UiO‐66 metal‐organic frameworks (MOFs) infused with silicone oils to prepare SLIPS with anti‐icing capabilities.^[^ ^19^, ^20^ ^]^ However, the improvements were limited due either to a lack of adequately fine surface texture or to the use of MOFs without a rational consideration for the pore size and chemistry. The surface slipperiness–marked by ready slippage of droplets on them at < 10° surface inclination – was lost in < 2 hrs under continuous water shedding or the ice‐adhesion strength increased (> 20 kPa) just after 10 icing/de‐icing cycles. In another example,^[^ ^21^ ^]^ silane functionalized UiO‐66 MOF infused with silicone oil with a viscosity of 500 cSt (designated as Si500 henceforth) failed in less than 30 min of water shedding, i.e., steady dripping of droplets and allowing them to slide past the surfaces. A random combination of MOFs with lubricants is not likely to succeed.

Another common issue for SLIPS is the cloaking (coverage) of droplets by a film of lubricant due to strong interactions between the oil and the droplet liquid. Continuous droplet shedding with cloaking accelerates the lubricant drainage on SLIPS.^[^ ^12^ ^]^ Recently, viscous lubricants were shown to minimize the cloaking of droplets of ethanol (i.e., a low surface tension liquid).^[^ ^22^ ^]^ Unfortunately, this strategy does not work for water droplets, and the high viscosity lubricants also lower the droplet mobility, which is inversely proportional to the oil's viscosity.^[^ ^10^ ^]^ Solid and/or highly viscous liquid lubricants show prolonged performance in anti‐icing and condensation applications.^[^ ^22^, ^23^ ^]^ However, such surfaces remain susceptible to high‐speed liquid impacts. We need a better and more rational strategy to “hold” low‐viscosity lubricants on the surface for optimal drop mobility and improved impact resistance. For liquid impacts, a convenient way to quantify the intensity of impact, independent of liquid properties is to determine the liquid Weber number (We), which is the ratio of liquid kinetic energy to interfacial energy. Indeed, SLIPS in the literature reporting liquid impact investigation is rare; Ma et al. are notable to this end,^[^ ^24^ ^]^ they used porous polytetrafluoroethylene (PTFE) films to hold perfluorinated lubricants and tested them up to We∼10^4^. Raindrops in harsh (stormy) weather can readily exceed this. Furthermore, the use of fluorinated materials – particularly per and polyfluoroalkyl substances (PFAS) – with strong environmental and toxicity concerns is a major challenge.^[^ ^3^, ^19^, ^25^, ^26^, ^27^, ^28^, ^29^, ^30^ ^]^ In an attempt to address these limitations, we present a rational three‐part strategy that exploits a combination of strong capillarity in MOF pores, molecular intercalation, and strong host‐guest interactions to overcome cloaking, shear from sliding drops, and impact from drops/liquids that cause the lubricant depletion. Exploiting the rational combination of reticular chemistry and lubricant chains at the molecular level also enabled the desirable durability of fluorine‐free SLIPS.

## 2 Results and Discussion

### 2.1 Rational Three‐Fold Strategy

**Figure 1:**
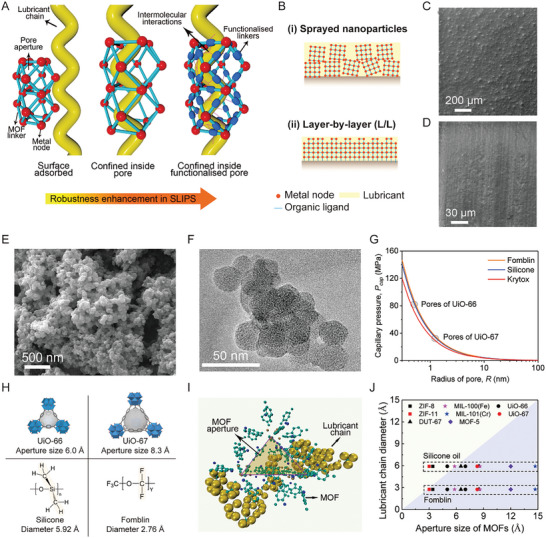
Robust SLIPS design strategy and intercalation of lubricants into MOF pores. A) Schematics depicting the need for a rational combination of MOF pore size, surface chemistry, and lubricant to enable size‐dependent lubricant intercalation and strong oil entrapment on the surface. Left: poor SLIPS design where lubricant is not able to enter the MOF pore due to size mismatch and is adsorbed on the surface. Middle: successful intercalation into as‐prepared MOF offering only physical confinement with limited stability. Right: intercalation in functionalized and size‐matched MOF pores with enhanced host‐guest interactions for maximal robustness. Red spheres indicate metal clusters, cyan lines represent organic linkers, and blue dots are hydrophobic functional groups (alkyl or fluoroalkyl). B) Schematics showing the fabrication of SLIPS using two different techniques: spraying MOF nanoparticles and layer‐by‐layer (L/L) grown on substrates. C) SEM images showing the morphology of a surface sprayed with the alkyl functionalized UiO‐66 nanoparticles, followed by a lubricant (Fomblin) infusion. D) SEM image of surface‐grown alkyl functionalized UiO‐66 on aluminum infused with Fomblin. E) SEM image showing morphology, and F) TEM image showing porosity of the UiO‐66 nanoparticles. G) Capillary pressure of lubricants inside MOF based on Young‐Laplace equation. Pore radii for UiO‐66 and UiO‐67 are marked by two circles. H) Schematics showing two MOFs with their aperture sizes and two lubricants with different diameters. White balls represent the inner pore space of MOF. I) Intercalation and confinement of a lubricant chain (yellow color) inside the octahedral UiO‐66 pore (the triangular aperture is highlighted for clarity). J) The diameters of silicone oil and Fomblin lubricant chains against the aperture size of the different MOFs including ZIF‐11, DUT‐67, MIL‐100, MIL‐101, and the ones used in this study, ZIF‐8, UiO‐66, MOF‐5, and UiO‐67.

**Figure** **1**A schematically shows the details of our three‐fold strategy for robust SLIPS. First, the use of MOF with sub‐nanometre pores enabled us to achieve high capillary forces between oil and the surface. Second, we selected MOF with appropriate pore sizes to fit the diameter of the lubricant molecular chains to allow its intercalation into the aligned MOF pores. Third, the chemistry of the MOF organic linkers was altered through post‐synthetic functionalization to facilitate a strong host‐guest interaction between MOF and the lubricant, and to elicit the formation of a supramolecular structure. To establish this rational approach, a range of MOFs with different pore sizes (i.e., ZIF‐8, UiO‐66, UiO‐67, and MOF‐5) and chemistries (i.e., ─NH_2_, ─CH_3_, and ─CF_3_) were synthesized and used in combination with various lubricants (i.e., silicone, Krytox, and Fomblin). The chemical structures and physical properties of the lubricants are summarized in Figure S1 and Table S1 (Supporting Information). The hydrophilic UiO‐66 (with ─NH_2_ functionalized linker) is designated as NH_2_‐UiO‐66, whereas those functionalized with hydrophobic alkyl (─CH_3,_ with n = 8 carbons) and fluorine (─CF_3_, n = 7 carbons) chains are abbreviated as A‐UiO‐66 and F‐UiO‐66, respectively. MOF‐5 and UiO‐67 followed the same abbreviation rules.

The SLIPS were prepared by infusing lubricants into MOF particles either sprayed or grown on substrates using the layer‐by‐layer (L/L) technique (Figure 1B), the latter expectedly resulted in better mechanical integrity of the MOF layer due to covalent bonding with the substrate. The morphologies of the sprayed and L/L surface‐grown UiO‐66 MOF on aluminum are presented in Figure 1C,D, respectively. The sprayed surface was relatively rougher (Figure 1C), and as expected, Figure 1D shows the highly uniform surface obtained from L/L grown MOF nanoparticles. To achieve the desired surface chemistry, post‐synthetic functionalization was performed on the MOF before spraying and after L/L growth on the substrate (see Experimental Section for details). Scanning electron microscope (SEM) image of UiO‐66 nanoparticles are presented in Figure 1E (other MOF particles are presented in Figure S2, Supporting Information). The nanoporous structure of the UiO‐66 nanoparticles shown in Figure 1F, was confirmed using a transmission electron microscope (TEM). The corresponding chemical and crystalline structures before and after functionalization were confirmed by Fourier transform infrared spectroscopy (FTIR) and powder X‐ray diffraction (PXRD) and the data is presented in Figures S3–S5 (Supporting Information). The specific peaks at 1690, 1230, and 2926 cm^−1^ in FTIR spectra can be attributed to stretching of the C═O, C─F, and C─H bonds, respectively, confirming the successful linkage of alkyl and fluorine chains to the MOF linkers. The consistency of the main diffraction peaks in PXRD patterns of functionalized UiO‐66 suggested that the substituted side chains did not alter the MOF crystal structure.^[^ ^31^, ^32^, ^33^ ^]^ Height profiles of the sprayed (∼20 µm thick) and L/L grown (∼2–3 µm thick) UiO‐66 MOF surfaces on a metal substrate were obtained using focused ion beam SEM (details in Figure S6, Supporting Information).

To decipher the importance of oil nanoconfinement, we started with SLIPS comprising silicone oil and fluorinated lubricants, including Fomblin and Krytox, infused into pristine UiO‐66 without any functionalization. The capillary pressure (P_cap_) for UiO‐66 pores was estimated using the Young‐Laplace equation (Figure 1G). The ultra‐high capillarity was attributed to the sub‐nanoscale aperture size of UiO‐66 ∼6 Å^[^ ^34^ ^]^ (Figure 1H). The water droplet advancing contact angle (θ_Adv_) and contact angle hysteresis (Δθ) were measured for SLIPS with the four different lubricants infused into UiO‐66 (Table S2, Supporting Information). Next, as a first test for stability, we subjected the SLIPS to water shedding, which involves dripping water drops on the surfaces tilted at 45° and allowing them to slide off. The shedding test was performed in cycles, where each cycle consisted of dripping 50 mL water over a ∼60 min time span followed by contact angle measurements either immediately afterward or after a recovery period in which the lubricant was observed to recover and heal the impact zone. The sliding water droplets gradually deplete the lubricant from the surface through shear forces and/or encapsulation of the droplet by the lubricant film, so‐called “cloaking”.^[^ ^10^ ^]^ The SLIPS prepared from the pristine UiO‐66 failed quickly; droplets started to get pinned on the corresponding silicone oil‐based SLIPS within the first hr of the shedding test whereas the ones with fluorinated lubricants performed slightly better, sustaining slipperiness for ∼4 hrs (Table S2, Supporting Information).

To gather first insights into this early failure and differences among silicone and fluorinated oil‐infused surfaces, we investigated the intercalation of lubricant chains into the highly organized UiO‐66 pores^[^ ^35^, ^36^ ^]^ using long‐time molecular dynamic (MD) simulations (see Experimental Section). The lubricant chain diameters, calculated as the sum of branch bond lengths using the quantum method PM7, were 2.76 Å for Fomblin and ∼5.92 Å for the silicone, which explains the differences in their intercalation into UiO‐66 pores (Figure 1H). The diameter of the silicone oil chain was very close to the aperture size of UiO‐66 (∼6 Å), resulting in extremely strong intermolecular repulsion (scaling as ∼1/r^12^ according to Lennard‐Jones theory, with r being the atomic separation). The simulations also made it clear that the Fomblin chain could enter the UiO‐66 pores (Figure 1I), whereas the silicone chain was too large to do so (see Figure S8, Movie S1, Supporting Information). Additionally, and as expected, the silicone chain could fit comfortably in the bigger pore (∼8 Å) of UiO‐67 (Figure 1J). Following this geometric principle, Figure 1J summarises the relevance of pore sizes of different MOFs against the diameters of the silicone and Fomblin chains, and serves as an initial guiding step for the design of MOF‐based SLIPS.

**Figure 2:**
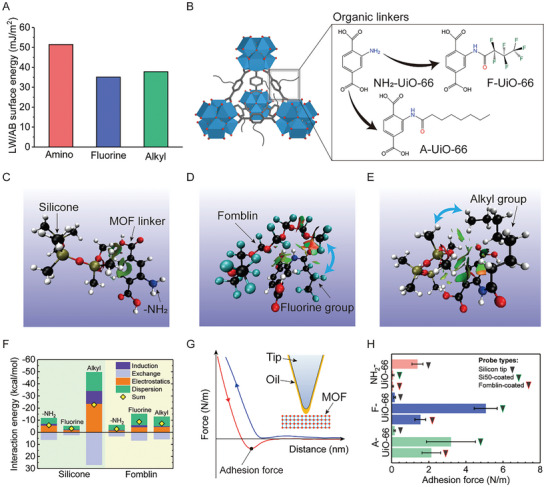
Enhanced host‐guest interactions for robust SLIPS. A) Surface energy for different functional groups was calculated using the LW/AB method. B) Schematics of functionalized UiO‐66 MOF. Molecular structures of the organic linkers in hydrophilic amino (NH_2_‐UiO‐66), alkyl (A‐UiO‐66), and fluorine functionalized (F‐UiO‐66) UiO‐66. Gradient isosurfaces for C) amino functionalized linker and silicone chain, D) fluorine functionalized linker with Fomblin chain, and E) alkyl functionalized linker with silicone chain. Colored balls represent different atoms: white is H, red is O, blue is N, black is C, cyan is F, and olive is Si. The two‐way blue arrows indicate the interaction between lubricants and functional groups. F) Contribution of the SAPT derived constructive dispersion, electrostatic, induction, and exchange forces toward interaction energy between different combinations of functionalized linkers and lubricants. ─NH_2_, alkyl, and fluorine represent the amino functional group, alkyl chain, and fluorine chain, respectively. G) Schematic illustration of AFM adhesion force measurement and representative force‐distance curves of MOF and lubricant. The approach and retract curves were blue and red, respectively. Adhesion force was determined from the minimal point during the retracting of probes. H) Lubricant‐MOF adhesion forces obtained from atomic force microscope force–distance curves. Silicon tips are used as a control.

To understand the host‐guest interactions that facilitate the intercalation of oil into the MOF pore and also, the interaction, an examination of surface energies and the interactions between different lubricants and the MOF functional groups is required. Our contact angle measurements combined with Lifshitz–van der Waals/acid–base (LW/AB) method^[^ ^37^ ^]^ show the expected surface energy trend for functional groups to be: ─NH_2_> ─CH_3_ > ─CF_3_ (**Figure** **2**A, see methodology details in Section 4.2.1, Supporting Information). This suggests easy wetting of MOF with ─NH_2_ terminated linker by oils due to their low surface energy, but that does not necessarily point to a strong interaction. Thus, for a deeper insight, different UiO‐66 functionalization (Figure 2B) and lubricant combinations were investigated both, theoretically using density functional theory (DFT) and experimentally using an atomic force microscope (AFM). Electron density and its derivatives obtained from DFT were first used to compute the reduced density gradient (RDG) function.^[^ ^38^ ^]^ RDG was then used to analyze the strength of noncovalent interactions. The resulting interaction isosurfaces for different combinations are presented in Figure 2C–E (the complete set is presented in Figure S9, Supporting Information). The location of the isosurfaces, particularly those indicated by blue double‐sided arrows, offers some insight into which molecular groups have van der Waals interaction. For example, with a hydrophilic ─NH_2_ group added to the MOF linker, the isosurface appears between the linker's benzene ring and the methyl group (Figure 2C) in the lubricants. On the other hand, it appears between the perfluoro groups in Fomblin and fluorine functionalized linker (Figure 2D) or the methyl groups from the alkyl functionalized linker and the silicone oil (Figure 2E). Similarly, we also calculated electrostatic potential (ESP) using the DFT data (Figure S10, Supporting Information), which captures the progressive changes in the electrostatic potentials for different lubricant/linker functionalization combinations. Overall, these point to the electropositive nature of methyl groups and the strong electronegativity introduced by fluorinated groups. However, the individual interaction components are unclear. To address this, we used symmetry‐adapted perturbation theory (SAPT) which could separate the contributions from electrostatic, dispersion (London), induction (Debye), and exchange (repulsion) interactions (Figure 2F). In agreement with the results from RDG calculations, the sum of dispersion and induction (i.e., van der Waals) interactions in Figure 2F is markedly greater for each lubricant chain and hydrophobic linker combination compared to that for the hydrophilic linker. Additionally, the electrostatic interaction in Figure 2F is highest for alkylated linkers and silicone oil. Overall, the DFT results point to a need for matching the functional groups grafted to the MOF linked with those in the lubricant carbon chain, rather than simply relying on surface energy calculations.

Although offering clarity on the nature/types of oil and MOF interactions, the DFT calculations are naturally constrained by computational costs. Indeed, we could only use limited lubricant chain length, i.e., number of carbon atoms, and thus are unable to offer a quantitative assessment of the lubricant and MOF interactions. Therefore, the lubricant/MOF adhesion forces were measured using AFM (as shown in Figure 2G). Essentially, the force‐distance curves obtained using lubricant‐coated silicon tips and MOF particles were used to calculate the adhesion forces (as illustrated in Figure 2G and the methodology details in Experimental Section). The measurement using dry silicon tips and UiO‐66 nanoparticles was used as a control. Only the relatively low‐viscosity lubricants, including Si50 (a viscosity of 50 cSt) and Fomblin, were used to avoid errors due to the formation of a stable capillary bridge between the AFM tip and the MOF particles.^[^ ^39^ ^]^ Overall, these measurements indicate significantly stronger adhesion (interaction) forces between hydrophobic MOF and lubricants compared to NH_2_‐UiO‐66/Si50 and NH_2_‐UiO‐66/Fomblin (Figure 2H). This confirms that matching the surface functionalization with the lubricant chemistry (e.g., A‐UiO‐66/silicone and F‐UiO‐66/Fomblin) will result in significantly enhanced host‐guest interactions.

### 2.2 Experimental Corroboration of Lubricant Intercalation into MOF Pores

**Figure 3:**
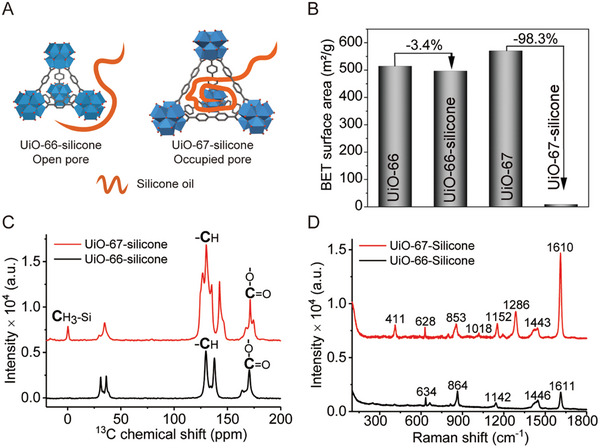
Experimental evidence of intercalation of lubricants into MOF pores. A) Schematics showing the occupied pore of UiO‐67 by silicone oil chains will lead to a decrease in surface area. B) Change in the BET‐surface area after successful infusion of silicone oil into UiO‐67 pores. C) ^13^C‐NMR, and D) Raman spectra confirming intercalation of silicone oil.

To verify the lubricant intercalation and nanoconfinement experimentally, we followed a similar approach to prepare SLIPS and analyzed them using various techniques such as BET‐surface area, ^13^C‐NMR, and Raman Spectroscopy. Following silicone oil infusion, the BET‐surface area reduced by 98.3% in UiO‐67 and only by 3.8% in UiO‐66, which indirectly confirms the penetration of the lubricant chains into the UiO‐67 pores with bigger aperture (**Figure** **3**A,B). The presence of Si‐Me carbon peak at ∼1 ppm in UiO‐67 ^13^C solid‐state NMR spectra again confirmed the successful loading of the lubricant chains into the MOF pore (Figure 3C). Similarly, the Raman spectra in Figure 3D show a clear peak at 411 cm^−1^, attributed to the Si‐O stretching, which is distinct from the UiO‐67 MOF characteristic peaks at 1610 cm^−1^ (C═C aromatic stretching), 1443 cm^−1^ (carboxylate ─COO symmetric stretching), 1286 cm^−1^ (C─C inter‐ring stretching), 1150 cm^−1^ (C─C symmetric ring breathing in linker), and 628 cm^−1^ (C─C─C aromatic ring in‐plane bending). This direct evidence further confirms the successful intercalation of the lubricant into the UiO‐67 pores. The importance of lubricant/pore size consistency was also verified using water‐shedding tests (Table S3, Supporting Information). The combinations of UiO‐66/Fomblin and UiO‐67/Si500 maintained slipperiness (determined by Δθ ≤ 10°) for up to 3–4 water shedding cycles, well beyond UiO‐66/Si500, which does not even last one cycle, underscoring the size dependence of intercalation and its role in enhancing the SLIPS durability. As an additional, indirect confirmation, θ_Adv_ for UiO‐66/Fomblin and UiO‐67/Si500 combination remained at ∼90° and ∼40° even after 10 continuous water shedding cycles. This was in sharp contrast to the pristine MOF surfaces, before any lubricant infusion, which was super hydrophilic and showed a hemi‐wicking behavior when a water droplet was placed on them. The finite values of θ_Adv_ measured even after multiple shedding cycles strongly point to size‐dependent intercalation. Lastly, as a visible confirmation of the lubricant intercalation, the infusion of oil into MOF pores should alter the roughness and light scattering property, thereby also influencing the optical transparency of the surfaces. This was apparent for all our SLIPS. For example, for the F‐UiO‐66/Fomblin combination, before and after lubricant infusion, the surfaces exhibited respectively ≤ 80 and ≥ 90% transmittance in the visible spectrum (Figure S13, Supporting Information).

### 2.3 Dynamic Shear Resistance

For further improvement in SLIPS durability, the pores of UiO‐66 were modified^[^ ^40^ ^]^ by covalently attaching low‐energy molecular chains (Figure 2B and synthesis procedure in Experimental Section), which could engender host‐guest intermolecular interactions, as investigated theoretically above. The wettability parameters of the sprayed MOF and lubricant‐infused surfaces are summarized in Figures S14 and S15 (Supporting Information). The SLIPS with Krytox and Fomblin showed comparable stability due to similar chemical structures. Hence, only Fomblin, Si50, and Si500 were used as lubricants for further stability investigations. The control surface was a rough boehmite substrate infused with Si500 (see Experimental Section and Figure S16, Supporting Information for fabrication details).

**Figure 4:**
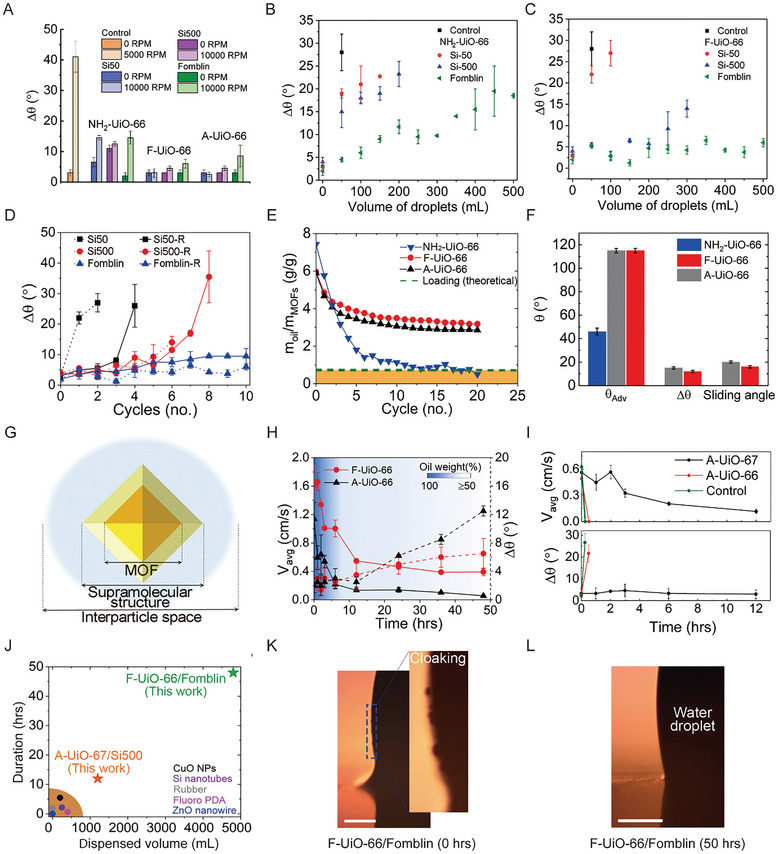
Spinning and water shedding tests. A) Change in Δθ of various SLIPS before and after spinning at 10,000 RPM. The control sample failed after spinning at 5000 RPM. The change in ∆θ caused by lubricant depletion from B) NH_2_‐UiO‐66 and C) F‐UiO‐66 was recorded for 10 water‐shedding cycles. The volume of water in each cycle is 50 mL. D) Change in Δθ on F‐UiO‐66 infused with different lubricants measured immediately following water shedding cycles (dotted lines) and after the recovery period (solid lines). E) Evolution of the lubricant (Fomblin) mass (m_oil_), normalized with the mass of MOF particles, with shedding cycles capturing final oil retention. The Green dashed line shows the theoretical loading capacity of UiO‐66 for Fomblin. The retained lubricant mass for the two hydrophobic MOFs plateaus down to a higher threshold and never falls to the orange area under the theoretical capacity for the pristine (unfunctionalized) MOF. F) Wetting angles (θ_Adv_, Δθ, and sliding) for the Fomblin containing SLIPS on different UiO‐66 MOFs, measured after reaching the plateau in the lubricant content. G) Schematic representation of the lubricant immobilization inside frameworks and the formation of a stable, supramolecular structure, contributes to the excellent stability of our SLIPS. H) Lubricant retention and surface slipperiness were tracked by measuring average droplet sliding velocity (V_avg_) on the surface and Δθ over a long (50 hrs) water shedding testing on Fomblin/A‐UiO‐66 and Fomblin/F‐UiO‐66. I) Evolution of surface slipperiness (droplet velocity and Δθ) over 12 hrs water shedding test on Si500/A‐UiO‐67. J) Comparison of the A‐UiO‐67/Si500 and F‐UiO‐66/Fomblin SLIPS introduced here against state‐of‐the‐art SLIPS in terms of stability against dripping water. Optical images showing wetting ridge formation on L/L F‐UiO‐66/Fomblin SLIPS, K) before, and L) after the water shedding test. The inset shows micro‐oil droplets and a clear wetting ridge on the interface of a 10 µL water drop placed at the freshly infused surface. No wetting ridge or cloaking was observed after long‐term (50 hrs) shedding while the surface still maintained its slipperiness. The scale bar is 200 µm.

Lubricant retention on the sprayed UiO‐66 MOF was first tested by subjecting the surfaces to high spinning speeds for 60 s (details in Experimental Section) followed by measurement of contact angles (**Figure** **4**A). The control surface and NH_2_‐UiO‐66‐based SLIPS suffered rapid lubricant depletion. In contrast, another two MOF‐based SLIPS maintained self‐cleaning properties, i.e., Δθ < 10°. The mass change of lubricant and contact angle changes with various rotational speeds ranging from 100 to 10000 RPM are presented in Figures S17 and S18 (Supporting Information). Three UiO‐66‐based SLIPS retained ∼50–60% of all different types of oil even after spinning at 10000 RPM. Clearly, as a volumetric force, centrifugal force was much smaller than the capillary force. Therefore, a harsher water‐shedding test was conducted next to assess lubricant retention.

Figure 4B,C show the evolution of surface wettability of NH_2_‐UiO‐66 and F‐UiO‐66 based SLIPS under water shedding test, respectively. For NH_2_‐UiO‐66/silicone combinations, Δθ increased dramatically after shedding droplets totaling 50 mL in volume, i.e., one shedding cycle; from ∼2° to ∼19° and from ∼3° to ∼15° for Si50 and Si500, respectively (Figure 4B). Fomblin infused into hydrophobic UiO‐66 MOF showed much better resistance to water shedding (Figure 4C). This is to be expected from the discussion above regarding the pore size of UiO‐66 being small for silicone chains and the stronger interaction of F‐UiO‐66 with the lubricants. SLIPS with silicone lubricants infused in F‐UiO‐66 were less stable, with Si500 fairing slightly better and retaining the slippery behavior for 4/5 cycles. Given the larger size of silicone chains compared to F‐UiO‐66 pores, the 4/5 cycles of stability must emerge from oil adsorption and retention in between the MOF nanoparticles, i.e., interparticle spaces, as a whole. A‐UiO‐66‐based SLIPS showed similar performance in retaining lubricant with a smaller size, i.e., Fomblin, but was relatively less durable (Figures S19 and S20, Supporting Information). To further verify the geometric compatibility, different MOFs with pores in the 4 ‐12 Å range, such as ZIF‐8 (4 Å), A‐UiO‐66 (6 Å), A‐UiO‐67 (8.3 Å), and A‐MOF‐5 (12 Å) were investigated with silicone lubricant Si500. As expected, for MOFs with smaller pores (ZIF‐8 and A‐UiO‐66), where silicone should not penetrate, the SLIPS failed within the first shedding cycle (Figure S21, Supporting Information). In contrast, Si500‐based SLIPS with bigger MOF pores, e.g. A‐UiO‐67 and A‐MOF‐5, preserved their slipperiness (Δθ ≤ 10°) for at least 8 water shedding cycles. The slightly earlier failure of the SLIPS prepared from A‐MOF‐5 was perhaps due to large interparticle gaps among inherent large crystals (∼10 µm, see Figures S22 and S2D, Supporting Information).

The reticulated porosity of MOFs offers an additional benefit: any local drainage of lubricant may be replenished from neighboring parts through seepage. This for example can enable “healing” of the local oil depletion introduced by water drop shedding tests. For proof‐of‐concept, SLIPS samples comprising F‐UiO‐66 infused with Si50, Si500, and Fomblin were used. To assess the recovery times, we measured Δθ, both immediately following a water‐shedding cycle and after a certain recovery period (Figure 4D). Δθ increased sharply from ∼3° to ∼22° for the Si50‐infused SLIPS immediately after the first cycle of water shedding. Likewise, Δθ for SLIPS infused with Si500 increased from ∼2° to ∼15° after 4 cycles. In contrast, following a recovery period, Δθ remained under ∼10° after 3 and 6 shedding cycles on the Si50 and Si500‐infused SLIPS, respectively. Figure S23, Movie S2 (Supporting Information) capture the gradual healing due to seepage of the oil from neighboring interparticle gaps and the MOF pores following water shedding. The recovery periods for the Si50, Si500, and Fomblin were ∼30, ∼180, and ∼60 min, respectively, showing consistent increases in the oil viscosity. Following the recovery period, the surfaces were then subjected to another round of water shedding. With Si50, the surface became dry after the second shedding cycle. Si500, which has a similar chemical composition to Si50 but higher viscosity, showed replenishment up to seven shedding cycles possibly due to high initial oil loading (Table S4, Supporting Information) and better shear resistance. The F‐UiO‐66/Fomblin combination performed best, showing full recovery even after 10 water‐shedding cycles. Additionally, the depleted area of Fomblin was smaller than that for the silicone lubricants, possibly due to Fomblin's excellent confinement in the F‐UiO‐66 nanopores, higher affinity to the functionalized UiO‐66, and weaker interactions with water.^[^ ^41^ ^]^

After the 10th cycle of water shedding, the Fomblin mass on A‐UiO‐66 and F‐UiO‐66 started to plateau, whereas the NH_2_‐UiO‐66 showed an unstable weight loss pattern (Figure 4E). The final (retained) lubricant masses in both A‐UiO‐66 and F‐UiO‐66‐based SLIPS (> 3 g/g) were approximately half of the initial masses. Regardless of the functionalization type, a certain amount of lubricant was permanently held; A‐UiO‐66 and F‐UiO‐66 retained slippery behaviors with low sliding angles of ∼20° and ∼15° and Δθ of <13° and <10°, respectively. The contact angle remained ∼115° for both hydrophobic UiO‐66 MOFs and decreased to ∼46° for the one NH_2_‐UiO‐66 (Figure 4F).

To explain this striking superior stability of our SLIPS–with a rational combination of lubricant chain size, MOF pore size, and chemistry–we hypothesized the formation of a supramolecular structure (Figures 1A and 4G) in which the long lubricant chains are partially embedded in the porous MOF structure and the protruding part of the chains endows slippery behavior. However, unlike traditional thermodynamically controlled (formation of kinetically stable bonds) assembly of supramolecular structure,^[^ ^42^, ^43^, ^44^ ^]^ our structure is based on strong host‐guest interactions. This is akin to the interaction present in cyclodextrin‐based inclusion complexes.^[^ ^45^, ^46^ ^]^ These findings are supported by various chemical characterizations, including BET, Raman, and NMR characterizations, and high interaction energies calculated between the corresponding MOF functionalization and lubricant combination (e.g., alkyl/silicone and fluorine/Fomblin). We quantified lubricant retention on SLIPS and compared it against the theoretical loading capacity of UiO‐66 (0.72 g/g) for Fomblin (see Experimental Section section). The measured lubricant retention for NH_2_‐UiO‐66 was ∼0.5 g/g (Figure 4E), i.e., below the theoretical limit. This suggests that although the Fomblin chains entering the MOF pores (molecular cages) may remain trapped, those that are partly intercalated, lying on the particle surface and between the particles must have been depleted away due to weak interactions between amino and fluorine groups (Figure 2F). Conversely, with functionalized MOF particles, only the chains from the interparticle spaces are depleted under water‐shedding while strong guest‐host interactions lead to the formation of a layer of partially intercalated chains into a supramolecular structure (see Figure 4E,G), which is firmly retained and results in a much higher retention capacity.

For a long‐term stability assessment, water‐shedding was performed for 50 hrs at 100 mL hr^−1^ and with drops impacting on the SLIPS at ∼80 cm s^−1^ followed by sliding along the surface. Figure 4H shows that F‐UiO‐66 performed slightly better (Δθ ∼6°) than A‐UiO‐66 (Δθ ∼12°) due to stronger interaction of Fomblin with F‐UiO‐66, in agreement with results presented before. A shift in the characteristic peak of Fomblin (C─F stretching) from 1122 to 1188 cm^−1^ after infusion into F‐UiO‐66 was also observed due to strong F─F interactions (Figure S24, Supporting Information). The average speed of the droplets sliding (V_avg_) on the surfaces decreased from ∼1.2 to ∼0.2 and ∼1.6 to ∼0.4 cm s^−1^ on A‐UiO‐66/Fomblin and F‐UiO‐66/Fomblin, respectively, over the long test period. On the other hand, fluorine‐free SLIPS (prepared using A‐UiO‐67 and Si500 combination) retained slippery behavior for > 12 hr of water shedding (Figure 4I). In contrast, controls based on functionalized Al/Si500 and UiO‐66/Si500 failed within 1 hr. The drop sliding speed on A‐UiO‐67/Si500 went from ∼0.6 to ∼0.2 cm s^−1^.

Figure 4J summarises the excellent performance of our rational SLIPS, including fluorinated and fluorine‐free surfaces, prepared using sprayed nanoparticles against state‐of‐the‐art SLIPS,^[^ ^9^, ^17^, ^28^, ^47^, ^48^ ^]^ which survived only a couple of hours at most and sometimes as little as tens of seconds. Further, we investigated the cloaking of the sliding water droplets by the lubricants (see Figure 4K,L). Obvious cloaking, including wetting ridge, was observed before water shedding tests on F‐UiO‐66/Fomblin. However, after shedding tests, a clear and sharp contact line without any wetting ridge is observed on both SLIPS. Figure S25 (Supporting Information) showed the same phenomenon on A‐UiO‐66/Si500. Given that the drops still slide, the retention of lubricants becomes clear. This retained slipperiness (V_avg_ > 0) of our best‐performing SLIPS points to their stability gained through the three‐pronged, rational approach we have introduced in this work.

### 2.4 Enhanced Robustness and Liquid Impalement Resistance

**Figure 5:**
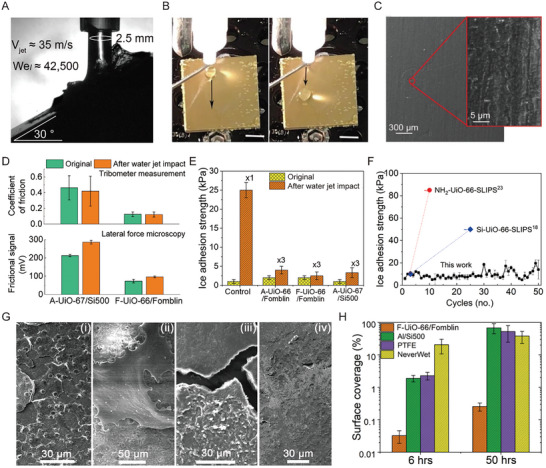
Jet impact resistance, friction, ice adhesion, and anti‐biofouling tests. A) Water jet (nozzlediameter = 2.5 mm) impacting at ∼35 m s^−1^. B) Free sliding of water droplets from the impact site after repeated jet impacts on F‐UiO‐66/Fomblin SLIPS (scale bar: 1 cm). C) SEM images showing morphological changes at the impact site. D) The friction coefficient and frictional signal measured before and after high‐speed water jet impacts (∼35 m s^−1^) on different SLIPS. E) Ice adhesion strength was measured before and after high‐speed water jet impacts (∼35 m s^−1^) on different SLIPS and compared with the control surface (rough aluminum/Si500). F) Ice adhesion strength comparison of robust SLIPS produced here (F‐UiO‐66/Fomblin) against previously reported MOF‐based SLIPS to confirm their poor design. G) SEM images of i) PTFE, ii) commercial NewerWet®, iii) Al/Si500, and iv) F‐UiO‐66/Fomblin showing biofilm formation after 50 hrs of incubation under the continuous flow of S. aureus culture and subsequent H) surface coverage quantified by ImageJ software.

While the SLIPS prepared by infusing the functionalized, sprayed MOF nanoparticles are good for assessing physical and chemical characteristics, formation of supramolecular structure, and developing a rational strategy to promote lubricant retention, these surfaces lack the overall mechanical robustness because the nanoparticles are held on the substrate with weak van der Waals forces only. Thus, we used L/L grown MOF to address this issue (see Experimental Section section). There are a few advantages to this. First, due to the absence of interparticle gaps, the reticulated structure of L/L MOF does not compromise the lubricant recovery discussed above. Second, the L/L MOF structure offers a defect‐free surface pattern (pores in sub‐nm size scale). The larger gap weakens the lubricant retention and with depletion, the overall increase in surface roughness is significant. This can eventually lead to a loss in surface smoothness which is at the core of SLIPS.^[^ ^3^ ^]^ The results from the long‐term water shedding test performed on L/L MOF‐based SLIPS, for the same set of functionalization/lubricant combinations as above, are shown in Movie S3, Figure S26 (Supporting Information). On L/L F‐UiO‐66/Fomblin SLIPS, after 50 hrs of shedding tests, the V_avg_ was ∼0.5 cm s^−1^, slightly faster than observed on the sprayed samples–this is reasonable since the L/L MOFs have a low roughness and nano‐hierarchical texture^[^ ^21^ ^]^–no cloaking and wetting ridge were observed (Figure 4L). For a much harsher test for SLIPS stability, we subjected the surfaces to a high‐speed water jet impacting at ∼35 m s^−1^ (**Figure** **5**A and Movies S4 and S5, Supporting Information). Both fluorinated and fluorine‐free SLIPS were able to resist jets up to ∼35 m s^−1^ with the corresponding liquid Weber number ∼42500, which may enable future outdoor usage unlike previously reported SLIPS. The surface slipperiness was assessed after three repeated jet impacts; water droplets readily slid off from the location of impact (Figure 5B; Figure S27, Supporting Information). The impact location was investigated using SEM, which showed superficial erosion and removal of the upper MOF layer without altering the overall structure (Figure 5C). The excellent mechanical integrity of the MOF layer emerged from the covalent bonding between MOFs and substrates (Figure S28, Supporting Information) and enabled the surfaces to sustain the tape peel test with 50 repeated cycles (Figure S29, Supporting Information) and pencil scratch test (Figure S30, Supporting Information).

The macroscopic tribological measurements using a tribometer, along with nanotribological measurements using a lateral force microscopy (LFM) mode in an AFM, further confirmed the robustness and slipperiness of SLIPS after jet impact (Figure 5D). The coefficient of friction and frictional signal on both fluorinated and fluorine‐free SLIPS exhibited minimal changes after impact. Notably, the fluorinated SLIPS showed relatively lower friction throughout the reciprocating shearing motion of steel balls (Figure S31, Supporting Information), probably benefiting from the low viscosity of Fomblin and the better intermolecular interaction between MOF and Fomblin. Similarly, lower friction was observed between the Fomblin‐coated tip and the F‐UiO‐66 surface (Figure S32, Supporting Information). The low friction properties of our robust SLIPS will benefit many applications, which we will demonstrate in the next section.

### 2.5 Anti‐Icing, Anti‐Fouling, and Anti‐Biofouling

Next, SLIPS using functionalized, L/L MOF surfaces were tested for durability in various applications, which rely on their low adhesion properties (Figure 5E,F). An ultra‐low ice adhesion strength of <10 kPa was observed on all rational SLIPS (i.e., both fluorinated and fluorine‐free versions in Figure 5E) (Movie S6, Supporting Information). As a measure of durability, the adhesion strength of the control SLIPS, i.e., rough aluminum/Si500, increased from ∼2 to ∼46 kPa after just 30 min of water shedding and to ∼24 kPa after a single water jet impact, respectively (Figure 5E; Figure S33, Supporting Information). In contrast, the ice adhesion strength on either of the two rational SLIPS remained under 5 kPa after repeated high‐speed jet impact tests (Figure 5E). Furthermore, our rationally designed SLIPS maintained the ultra‐low ice adhesion tested for 50 continuous icing/de‐icing cycles, whereas, the state‐of‐the‐art MOF‐based SLIPS^[^ ^21^ ^]^ failed to preserve anti‐icing characteristics beyond 20 cycles (Figure 5F).

The F‐UiO‐66/Fomblin SLIPS was also tested for dynamic anti‐fouling and anti‐biofouling. A custom chamber was designed (Figure S34, Supporting Information) to check and compare the resistance against the formation of calcium carbonate scaling and bacterial adhesion using S. aureus under dynamic conditions of velocity = 6 cm s^−1^ and wall shear stress = 8.5 mPa. For antifouling, the surfaces were placed in the middle of the chamber under a continuous flow of water containing scaling precursor, 1 mM of CaCl_2,_ and 2 mm of NaHCO_3_ at supersaturated conditions for 96 hrs. After that, the surfaces were rinsed with water and analyzed for crystal growth using SEM and energy‐dispersive X‐ray spectroscopy (EDS) (Figure S35, Supporting Information). Our F‐UiO‐66/Fomblin SLIPS performed exceptionally well, showing almost no crystal growth (< 4%) and maintaining θ_Adv_ = ∼119° and Δθ = 8°, whereas large crystals of CaCO_3_ were visible on the control samples (Figure S35, Supporting Information). The superhydrophobicity of commercial NeverWet coating was lost with θ_Adv_ changing from ∼152° to ∼70°. The total surface coverage with scaling for our SLIPS, Al/Si500, and NeverWet was recorded at < 4%, ∼81%, and ∼53%, respectively. For anti‐biofouling, surfaces were exposed to the continuous flow of dense S. aureus culture with OD_600_ = 0.3. The bacterial adhesion and biofilm formation were assessed after 6 and 50 hrs, respectively. The surface coverages were quantified using SEM (Figure 5G; Figure S36, Supporting Information). Bacteria colonies were visible on control samples, whereas none was observed on our SLIPS after 6 hrs. After 50 hrs, a thick biofilm formed on the Al/Si500 control; a biofilm was also visible on the other two controls, PTFE plate and NeverWet coating, but was less dense (Figure 5G). Few bacteria were spotted on SLIPS; the quantitative data of the surface coverage was presented in Figure 5H. Our SLIPS showed < 1% of coverage (with discrete cells) even after 50 hrs.

## 3 Conclusion

In conclusion, we sought to introduce and establish the need for a rational approach to lock the lubricant in SLIPS through geometric compatibility and host‐guest interactions between MOF and lubricants. Beyond simply relying on high capillary pressure from the small pore size of MOF, we identified the need to consider optimal aperture size and functionalization of MOF pores to enable the surfaces, including fluorine‐free SLIPS, to resist cloaking induced by continuous droplet shedding and the impacts of high‐speed water jets. The study demonstrates that after successful intercalation, the host‐guest interactions between MOF and lubricant can effectively lead to the formation of a stable supramolecular structure that can inhibit lubricant depletion and delay surface failure to a remarkable degree, resisting droplet shedding (shear) for over 50 hrs, preventing biofilms in dynamic conditions and even sustaining impacts of high‐speed liquid jets. Benefiting from the unique combination of lubricant chains and reticular materials, the obtained performance, including excellent anti‐icing, anti‐fouling, and anti‐biofouling characteristics should widen the applications for SLIPS and liquid‐repellent surfaces in general.

## 4 Experimental Section

### Sample Preparation—Synthesis of MOF Particles

Either a water‐based, green approach (for NH_2_‐UiO‐66 and NH_2_‐UiO‐67) or, a solvothermal technique (for UiO‐66, UiO‐67, ZIF‐8, and MOF‐5) were used to synthesize the various MOF particles.^[^ ^49^, ^50^, ^51^ ^]^ Specific and detailed synthesis procedures for each MOF type are presented in Section 3 (Supporting Information). Briefly, in the water‐based approach, sodium salt of organic linkers (2‐aminoterephthalic acid and 2‐amino‐4 4′‐biphenyldicarboxylic acid) and metal salts (ZrOCl_2_·8H_2_O) were solubilized in water and mixed slowly, forming MOF precipitates. After stirring for 12 hrs at room temperature, MOF crystals were centrifuged and washed 3 times with DI water to remove unreacted linker and sodium salt from the pores. The water that remained trapped in the pores was removed following the Soxhlet method in ethanol for 16 hrs at 120 °C. The resulting crystalline powder was dried under vacuum for 12 hrs at 100 °C.

In solvothermal synthesis, specified amounts (typically 50 mM) of organic linkers (terephthalic acid or 4 4′‐biphenyldicarboxylic terephthalic acid) and metal salts (ZrOCl_2_·8H_2_O and Zn (NO_3_)_2_·6H_2_O) were dissolved in dimethyl formamide (DMF) and the reaction mixture was kept 12 hrs at 120 °C. The obtained MOF particles were washed twice with DMF, acetone, and chloroform to remove unreacted chemicals. The resulting crystalline powder was dried for 12 hrs under vacuum at 100 °C.

### Sample Preparation—Post‐Synthetic Modification of MOFs

To alter the MOF pore chemistry, octanoic acid (OA) and heptafluoro‐butyric acid (HBA) were linked to the NH_2_ group present on the MOF linker. Dried MOF powder or substrate with L/L MOF was dispersed/immersed in DMF containing one equivalent of OA and HBA to the organic linker. Then, the solution was stirred/kept at 80 °C for 12 hrs. Following this, the particles/surfaces were washed twice with DMF and acetone, and three times with chloroform before drying under vacuum for 12 hrs at 100 °C.

### Sample Preparation—Particle Spraying

MOF particle dispersion in acetone was sprayed onto the cleaned metal/glass substrates using a spray gun. Briefly, 10 mg mL^−1^ of MOF particles were dispersed in acetone and then sonicated for 30 min to get a homogeneous suspension. The weight of each substrate was recorded to monitor the amount of deposited MOF. The suspension was sprayed with a commercial spray gun (Iwata Eclipse, ECL2000) using a nitrogen gas pressure of 2.5 bar as controlled from a nitrogen cylinder fitted with a controller and pressure gauge. The samples were dried for 12 hrs under vacuum at 100 °C.

### Sample Preparation—L/L Growth of MOFs

Following the self‐assembly of the organic linker, the substrate was then immersed in 25 mmm DMF solution of metal salt for 20 min at 120 °C followed by washing (which included 1 min sonication) and immersion in linker solution for another 20 min.^[^ ^21^ ^]^ This completed one cycle of MOF growth. More than 20 cycles were needed in total to achieve a relatively thick and uniform MOF coating. Finally, the surface was thoroughly washed by sonicating in DMF and then chloroform to remove metallic or linker aggregates. The surface was vacuum dried for 12 hrs at 100 °C.

### Sample Preparation—SLIPS Fabrication

Sprayed or L/L MOF (see Section 3.7, Supporting Information) surfaces were weighed and infused with the excess amount (∼20 times the MOF weight) of lubricant onto the horizontally placed surface maintained at 100 °C on a hot plate covered with a glass Petri dish. After infusion for 12 hrs, the samples were kept tilted at 90° for 1 h and then spun at 2000 RPM to get rid of excessive lubricant for all standard tests except the spinning test where surfaces were subjected to different spinning speeds ranging from 100–10000 RPM.

### Sample Preparation—Preparation of Control Surfaces

As a first control, aluminum substrates, cleaned in acetone, water, and isopropyl alcohol (10 min each), were immersed in a bath of boiling deionized water for 10 min. After etching, the samples were rinsed with deionized water and dried with nitrogen. Excess of Si500 oil was infused in the rough structures following the same procedure described above for 12 hrs at 100 °C. The samples were tilted at 90° for 1 h and then rotated at 2000 RPM for 1 min after cooled down to room temperature. As synthesized hydrophilic (NH_2_‐UiO‐66) MOF nanoparticles, sprayed and infused with Si500 lubricant were also used as the second control to study the effect of pore chemistry.

### Characterization and Stability Tests—Contact Angle Measurement

A bespoke setup consisting of an adjustable stage, retort stand, a syringe pump, a light source, and a zoom lens fitted to a CMOS camera was used for contact angle measurement.^[^ ^52^ ^]^ Contact angle hysteresis (Δθ) was calculated as the difference between advancing (θ_Adv_) and receding (θ_Rec_) angles, which were measured by processing the recorded video with MATLAB.

### Characterization and Stability Tests—Spinning Test

A spin coater (Laurell WS‐650Mz‐23NPPB) was used to study the retention of lubricants by different MOF. Oil‐infused substrates were spun at 100, 500, 1000, 2000, 5000, and 10,000 RPM for 1 min, followed by the measurement of the weight and Δθ.

### Characterization and Stability Tests—Water Shedding Test

The water‐shedding set‐up comprised of a water reservoir (plastic, 2 L water bag) connected via a flow controller to a syringe with a 0.5 mm outlet diameter. The surfaces were placed at 45° tilt stage and the syringe tip height was kept 3 cm. Water droplets (∼20 µL, ∼4 mm diameter) dripped from the syringe onto the center of the test surface. Two different rates, 50 mL h^−1^, and 100 mL h^−1^, were used to assess the SLIPS stability in this work.

### Characterization and Stability Tests—Jet Impact Test

A bespoke set‐up fitted with a nitrogen gas cylinder connected to a syringe (outlet diameter of 2.5 mm) via an electronic pressure valve was used to assess the impact resistance on SLIPS.^[^ ^7^, ^21^ ^]^ The maximum jet speed reached in the experiments was ∼35 ms^−1^ at 12 bar pressure and the time for each jet was ∼25 ms. The surfaces placed at the 30° tilt stage were impacted thrice at the same location and the process was recorded using a high‐speed camera (Phantom V411). After jet impact, the stability of surfaces was assessed by checking their slipperiness, followed by SEM imaging to check mechanical erosion.

### Characterization and Stability Tests—Ice Adhesion Measurement

A bench‐top icing chamber constructed using a heat‐exchanger, a rotary aluminum stage (to mount samples) connected to a refrigeration unit (FP50‐HL Refrigerated/Heating Circulator, Julabo) with bath fluids (H5, Julabo) was used for ice adhesion measurements.^[^ ^21^, ^52^ ^]^ A stepper motor‐controlled system with an extension rod connected to a force gauge (M4‐50, MARK‐10) was used to push the ice cuvettes at −25 °C. The system was operated using LABVIEW software to measure the adhesion forces.

### Adhesion Force Measurement using AFM

Adhesion forces between MOF particles (NH_2_‐UiO‐66, A‐UiO‐66, and F‐UiO‐66) and lubricants (Si50 and Fomblin) were measured using a Bruker Multimode 8 atomic force microscope (AFM) with PeakForce Quantitative Nanomechanics (PFQNM) in “tapping mode”. Lubricant‐coated AFM tips (with 8 nm radii) were used as probes and MOF particles were adhered to the piezo stage using double‐side carbon tapes. Force distance curves were obtained at ∼0.1 µm s^−1^ approach and retraction velocity at room temperature (20–25 °C). The ramp distance was 750 nm, and the maximum normal force applied on the mica surface was kept constant (750 nN) using a relative trigger threshold.

### Simulations and Calculations—Molecular Dynamics Simulations

Molecular dynamics simulations were conducted with the Large‐scale Atomic/Molecular Massively Parallel Simulator (LAMMPS)^[^ ^53^ ^]^ to investigate the intercalation of lubricant chains into MOF pores. The molecular structures, created using the Gaussian View program (see Figure S7, Supporting Information), were optimized using the PM7 method, and their atomic charges were calculated using the Merz‐Kollman ESP charge method.^[^ ^54^ ^]^ Periodic boundary conditions were applied with a 50 Å square box. The cut‐off for van der Waals and Coulombic interactions was set at 12.5 Å. The flexible UFF force field was employed. The particle‐particle particle‐mesh (pppm) simulation was used with a relative precision of 10^−4^. Each run was equilibrated for 0.6 ns at 300 K in the canonical (NVT) ensemble using the Nosé–Hoover thermostat^[^ ^55^, ^56^ ^]^ with a time step of 0.5 fs.

### Simulations and Calculations—Theoretical Loading Capacity of UiO‐66

The theoretical loading capacity (L_F_) of UiO‐66 for Fomblin was estimated by assuming that the volume occupancy of lubricant molecules inside MOF pores is similar to that of small molecules, L_F_ = V_MOF_ ρ_F_, where the specific volume (V_MOF_) of UiO‐66 pore^[^ ^57^ ^]^ is 0.379 cm^3^ g^−1^ and the density (ρ_F_) of Fomblin is 1.9 g mL^−1^.

### Statistical Analysis

One‐way ANOVA and Dunnet's multiple comparison tests were conducted for intergroup comparisons using the least significant difference method in R software. The comparisons involved data from contact angle measurements and droplet sliding velocity of SLIPS samples using different functionalized UiO‐66 during water shedding tests. A p‐value of less than 0.05 was deemed statistically significant. All results were expressed as the mean and standard deviation, based on at least three independent measurements.

## Conflict of Interest

MKT is involved in an upcoming spin‐out company from UCL which seeks to commercialise liquid‐repellent coating.

## Supporting information



Supporting Information

Supplemental Movie 1

Supplemental Movie 2

Supplemental Movie 3

Supplemental Movie 4

Supplemental Movie 5

Supplemental Movie 6

## Data Availability

The data that support the findings of this study are available on request from the corresponding author. The data are not publicly available due to privacy or ethical restrictions.
