# Supplementary material for: Designing Impact Resistance and Robustness into Slippery Lubricant Infused Porous Surfaces
Source: Adv Mater. 2024 Oct 29;36(50):2409818. doi: 10.1002/adma.202409818 (PMC11636059; doi:10.1002/adma.202409818)
Supplement: Supplementary file 1 — Supporting Information [file ADMA-36-2409818-s005.docx]

**Supplementary Materials**

**Designing Impact Resistance and Robustness into Slippery Lubricant Infused Porous Surfaces**

Vikramjeet Singh^1#^, Jianhui Zhang^1,3#^, Priya Mandal^1,3^, Dingyu Hou^1^, Ioannis Papakonstantinou^2^, Manish K. Tiwari^1,3,^*

^1^Nanoengineered Systems Laboratory, UCL Mechanical Engineering, University College London, London, WC1E 7JE, UK.

^2^Photonic Innovations Lab, Department of Electronic & Electrical Engineering, University College London, Torrington Place, London, WC1E 7JE UK

^3^Wellcome/EPSRC Centre for Interventional and Surgical Sciences, University College London, London, W1W 7TS, UK

***Corresponding author. Email:** [m.tiwari@ucl.ac.uk](mailto:m.tiwari@ucl.ac.uk)

**This file includes:**

1. Materials
2. Characterisation and instrumentation
3. Methodology
4. Supplementary text
5. Supplementary Figures S1 to S36
6. Captions for supplementary movies S1-S6
7. References
8. **Materials**

Aluminium substrates (annealed, 0.5 mm thick, 99.99% trace metals basis) were purchased from Alfa Aesar. All chemicals including, zirconium oxychloride octahydrate (ZrOCl_2_·8H_2_O), Zinc nitrate hexahydrate (Zn(NO_3_)_2_·6H_2_O), terephthalic acid, 2-methylimidazole, 2-amino terephthalic acid, heptafluorobutyric acid, octanoic acid, silicone oils (with 50 and 500 cSt viscosity, abbreviated as Si50 and Si500 in this paper), Fomblin, Krytox VPF 1525 (the fluorinated lubricants), dimethyl formamide (DMF), chloroform, acetone, isopropanol, calcium chloride (CaCl_2_), acetic acid, sodium hydroxide (NaOH), and sodium bicarbonate (Na_2_HCO_3_) were purchased from Sigma Aldrich. 2-amino-4 4'-biphenyldicarboxylic acid was purchased from CD BioSciences Inc. USA. All chemicals were used without further purification. Deionized (DI) water with resistivity 18.2 MΩ was used in all experiments.

1. **Characterisation and instrumentation**

Surface morphologies of MOF nanoparticles and SLIPS were imaged using scanning electron microscopy (SEM) (EVO25, Carl Zeiss, Germany). For SEM, the specimens were immobilized on a metal stub with double-sided adhesive carbon tape and coated with a thin gold film, then observed at 10 kV voltage and 10 pA current. A JEOL 4700F dual beam system (Focused gallium ion beam coupled SEM) was used to measure the thickness of sprayed and surface-grown MOFs at 3 kV. The powder X-ray diffraction (PXRD) of the MOF powders were obtained using a Stoe STADI-P spectrometer at ambient temperature, with tube voltage of 40 kV, tube current of 40 mA in a stepwise scan mode (5° min^-1^). Fourier-transform infrared spectroscopy (FTIR) spectra was recorded with a spectrophotometer (Spectrum Two^TM^, Perkin Elmer) in the range of 400 to 4000 cm^-1^. ^13^C-muclear magnetic resonance (NMR) spectra was obtained through a solid-state NMR using a Bruker Avance 300 spectrometer with 7.05 T wide-bore magnet at ambient probe temperature. High-resolution solid-state ^13^C spectra were recorded at 75.5 MHz using a standard Bruker 4 mm double-resonance magic-angle spinning (MAS) probe. Solid materials were packed into zirconia rotors of 4 mm external diameter and spun at the MAS frequency of 12 kHz. High-resolution solid-state ^13^C NMR spectra were recorded using cross-polarization (CP), MAS and high-power proton decoupling. Typical acquisition conditions for ^13^C CPMAS total suppression of spinning sidebands (TOSS) spectra were: 1H 90° pulse duration = 2.45 µs; contact time = 2 ms; recycle delay = 5 s. ^13^C chemical shifts are presented relative to tetramethylsilane which was calibrated using glycine (176.46 ppm). Raman spectra were recorded on Bruker (SENTERRA II) at 785 nm wavelength, 50 mW laser power and 1.5 cm^-1^ resolution. Adhesion forces between MOFs and lubricants were measured using a Bruker Multimode 8 atomic force microscope (AFM) and the method details are presented in section 4.2.5. The transmittance of surfaces before and after lubricant infusion was measured using UV-Vis spectroscopy (UV-3600 iplus, Shimadzu) in a wavelength range of 200 nm to 800 nm. Transmission electron Microscopy (TEM) was performed at 200 kV on a JEOL JEM-2100 F system with a Schottky field emission gun. The samples were prepared by dispersing the MOF powders in ethanol and then casting onto a copper grid mesh and followed by solvent evaporation in air. The TEM images were taken using a Gatan Onevier Camera with full 4 k × 4 k resolution. The BET-surface area was recorded using a Quantachrome surface area and pore volume analyser (Anton Paar Nova Touch). The MOF pores were activated through removal of solvent traces by immersing in chloroform for 3 days followed by drying for 12 hrs under vacuum at 100 °C. In a typical experiment, the activated MOF samples (~200 mg) were loaded into the analyser, after degassing at 150 °C for 2 hrs. Then samples were cooled to liquid nitrogen temperature (77 K), before carrying out adsorption and desorption of N_2_.

1. **Methodology**
   1. **Synthesis of pristine UiO-66 and UiO-67 nanoparticles**

Pristine UiO-66 and UiO-67 nanoparticles used in the intercalation (penetration of lubricants inside MOF architecture) studies were synthesised by solvothermal method. In case of UiO-66, terepthalic acid (0.41 g) and in case of UiO-67, 4 4'-biphenyldicarboxylic terepthalic acid (0.6 g) along with ZrOCl_2_·8H_2_O (0.8 g) were dissolved in 50 mL of DMF followed by heating for 12 hrs at 120 °C. The white precipitates were centrifuged and washed thrice with DMF and acetone followed by drying for 12 hrs at 100 °C under vacuum before using them in different experiments.

- 1. **Synthesis of NH_2_-UiO-66 and NH_2_-UiO-67**

A green method was adopted to produce the NH_2_-UiO-66 and NH_2_-UiO-67 nanoparticles[1]. Briefly, the sodium salt of organic linker 2-aminoterephthalic acid or 2-amino-4 4'-biphenyldicarboxylic terepthalic acid was obtained using 2 equivalents of NaOH in distilled water at 60 °C for 10 minutes with constant stirring. The mixture was then left to cool to room temperature. In the second step, ZrOCl_2_·8H_2_O was solubilized in DI water using 100 mM acetic acid. Both the above prepared solutions were mixed slowly, forming a yellow precipitate within 5 minutes. The mixture was then stirred for 12 hrs at room temperature. The obtained MOF crystals were centrifuged and washed 3 times with DI water to remove unreacted linker and sodium salt from the pores. Remaining water trapped in the pores was removed following the Soxhlet method in ethanol for 16 hrs at 120 °C. The resulting crystalline powder was dried for 12 hrs under vacuum at 100 °C.

- 1. **Synthesis of ZIF-8**

Solvothermal method was used for ZIF-8 synthesis as well[2]. A mixture of Zn(NO_3_)_2_·6H_2_O (0.67 g) and 2-methylimidazole (0.167 g) in 50 mL of DMF was stirred until a clear solution was obtained. The solution was incubated at 140 °C for 24 hrs in hot air oven. The precipitates were filtered after cooling down the reaction mixture, washed thrice with DMF and acetone several times and kept in MeOH for 3 days and dried for 12 hrs under vacuum at 100 °C.

- 1. **Synthesis of NH_2_-MOF-5**

NH_2_-MOF-5 was synthesised using solvothermal method adopted from previous published reports with some modifications[3, 4]. No modulator has been used during synthesis. Zn(NO_3_)_2_·6H_2_O (1.2 g) and 2-aminoterephthalic acid (0.34 g) were dissolved in 40 mL DMF in a glass bottle. Then the solution was kept at 120 °C for 24 hrs. The reaction products were cooled to room temperature, and the precipitates were collected by centrifugation, washed twice with DMF and acetone and dried for 12 hrs under vacuum at 100 °C.

- 1. **Post-synthetic modification of MOFs**

Pore chemistry of amino functionalised MOFs (NH_2_-UiO-66, NH_2_-UiO-67, and NH_2_-MOF-5) was modified through a simple post-synthesis procedure. Octanoic acid (OA) and heptafluoro-butyric acid (HBA) were used to produce alkyl and fluorine functionalisation, respectively. For each case, the dried MOF powder was dispersed in DMF and 1 equivalent of OA and HBA to organic linkers (2-amino terephthalic acid and 2-amino-4 4'-biphenyldicarboxylic acid) was added to the solution and stirred for 12 hrs at 80 °C. Following this, crystals were centrifuged and washed twice with DMF and acetone, and three times with chloroform before drying under vacuum at 100 °C. Only alkyl functionalisation was performed on NH_2_-UiO-67 and NH_2_-MOF-5.

- 1. **Particle spraying**

The synthesized MOF particles were characterized for their morphology, chemical structure and crystallinity using SEM, FTIR, and PXRD, respectively (Supplementary Figures S2, S3, S4, and S5). Following that, particles were sprayed on metallic aluminium and glass coupons of various sizes followed by oil infusion to create the corresponding SLIPS. The substrates were sonicated for 15 minutes each in acetone, water and isopropyl alcohol to clean them before spraying. MOF particles dispersion in acetone was sprayed onto the substrates using a spray gun. Operation parameters (such as sonication time for uniform dispersion of MOFs in acetone, spraying pressure, nozzle size, MOF dilution, etc.) were tuned to obtain uniform MOF nanoparticle layer on substrates (Figure 1C). Briefly, 10 mg/mL of MOF particles were dispersed in acetone and then sonicated for 30 minutes to get a homogeneous suspension. The suspension was sprayed with a commercial spray gun (Iwata Eclipse, ECL2000) using a nitrogen gas pressure of 2.5 bar as controlled from a nitrogen cylinder fitted with a controller and pressure gauge. Approximately equal number of coatings were sprayed (15-17 spray passes) onto each surface to obtain thickness of ~10 µm. Acetone was then removed from MOF pores by drying the samples for 12 hrs under vacuum at 100 °C. The weight of each substrate was recorded to monitor the amount of deposited MOF.

- 1. **Layer-by-layer growth of MOF films**

The surface-grown MOF films were obtained using a layer-by-layer (L/L) growth technique[5]. In an optimal cycle of L/L growth of MOF, the aluminium substrate was immersed in 100 mL of DMF solution of MOF linker (25 mM) in a tightly closed glass bottle for 4 hrs to get uniform self-assembly of linker at 120 °C. The substrate was then rinsed in DMF and immersed in 25 mM DMF solution of ZrCl_2_.8H_2_O for 20 minutes at 120 °C followed by washing (which included 1-minute sonication) and immersion in linker solution for another 20 minutes. This completed one cycle of MOF growth. More than 20 cycles were needed in total to achieve a relatively thick and uniform MOF coating (Figure 1D). Finally, the surface was thoroughly washed by sonicating in DMF and then chloroform to remove metal or linker aggregates. The surface was vacuum dried for 12 hrs at 100 °C. The surface-grown MOF films were modified with alkyl and fluorine molecular chains as described above. Briefly, the MOF grown on aluminium surface was immersed in DMF solutions of OA/HBA and the solution was stirred for 12 hrs at 80 °C. Then the surfaces were washed twice with DMF and acetone, and three times with chloroform before drying under vacuum at 100 °C.

- 1. **SLIPS preparation**

The surfaces with sprayed MOF particles were placed horizontally on the hot plate and infused with different lubricants (silicone or perfluorinated) for 12 hrs at 100 °C followed by cooling down to room temperature. The details of lubricants used are presented in Supplementary Table S1. After completing the infusion process, the samples were tilted at 90° for one hour and then rotated at 2000 RPM for one minute to get rid of the excessive lubricant. The surfaces were subsequently weighed again to obtain the final mass of lubricant infused (Supplementary Table S4). The SLIPS were then subjected to various stability tests. SLIPS prepared from L/L grown MOFs followed the same infusion procedure. Thickness of the sprayed and L/L grown MOF layer was measured using FIB-SEM as presented in Supplementary Figure S6.

| **Lubricants** | **Viscosity**  **(cSt)** | **Density**  **(g cm^-3^)** | **Surface tension (mN m^-1^)** | **Vapor pressure**  **(Pa)** | **Miscibility** |
| --- | --- | --- | --- | --- | --- |
| Si50 | 50 | 0.96 | 21 | <700 | Isopropyl alcohol, hexane, pentane and toluene |
| Si500 | 500 | 0.97 | 21 | <700 | -- |
| Krytox | 250 | 1.88 | 16-20 | 5x10^-5^ | Only in per-fluoro hexane |
| Fomblin | 125-145 | 1.88–1.90 | 21-22 | n.a. | -- |

**Supplementary Table S1.** The properties of lubricants used in this study. The data is obtained from online material safety data sheets (MSDS) available at Sigma Aldrich^TM^. ^--^same as above.

- 1. **Control sample preparation**

Boehmite surface was used as a control in some of the stability testing which include spinning, water shedding, and jet impact. Uniform roughness (Supplementary Figure S16) was generated by reacting the metal substrate with water[6]. Briefly, as-received aluminium samples were thoroughly cleaned in acetone, water, and isopropyl alcohol, and then immersed in a bath of boiling deionized water (100 °C) for 10 minutes. After etching, the samples were rinsed with deionized water (15 minutes each) and dried with nitrogen. Next as described above, Si500 oil was infused in the rough structures for 12 hrs at 100 °C and then cooled down to room temperature. The samples were then tilted at 90° for one hour and then rotated at 2000 RPM for one minute to get rid of excessive lubricant.

- 1. **Contact angle measurement**

A bespoke setup was used for the contact angle measurement[5, 7]. The setup consists of an adjustable stage, retort stand, a syringe pump (World Precision Instruments, Aladdin single-syringe infusion pump), a light source (Thorlabs, OSL2) and a zoom lens (Thorlabs, MVL7000) fitted to a CMOS camera. Contact angle hysteresis (Δθ), employed as a criterion for assessing SLIPS slipperiness, was calculated from advancing (θ_Adv_) and receding (θ_Rec_) angles measured by processing the recorded video with MATLAB[5, 7].

- 1. **Spinning stability test**

Retention of lubricants by different MOFs was tested using a spinning stability test. The change in mass and Δθ on substrates infused with excess of lubricant were measured before and after spinning. Substrates were spun at 100, 500, 1000, 2000, 5000, and 10000 RPM for 1 minute. Change in Δθ and loss in lubricant mass were plotted in Supplementary Figures S17 and S18.

- 1. **Water shedding test**

The water droplet shedding test, considered to be a harsh test for oil retention capability of SLIPS, can drain the lubricant due to shear force and cloaking of the water droplet by the lubricant[8]. To carry out this test, a custom setup was used, which comprised a water reservoir (plastic, 2 L water bag) connected via a flow controller to a syringe. The syringe tip was kept at 3 cm height above the surface under test, which was inclined at 45° from the horizontal. Water droplets (~4 mm diameter) dripped from the syringe onto the test surface, at a cumulative volume flow rate of 150 mL/h for early shedding tests which were used to establish the rational choice for MOF pore size, lubricant choice and the corresponding pore chemistry (functionalisation). This created a continuous stream of drops impacting the surface at ~80 cm/s. The Weber number (We) and Reynolds number (Re) were calculated to be ~16 and ~2500, respectively (We=ρV^2^d/σ and Re=Vd/ν, where ρ denotes the liquid density, σ denotes the liquid surface tension, ν is the kinematic viscosity and V is the impact speed of droplets). The syringe volume (50 mL) was used as a measure of shedding cycle; ∆θ and lubricant mass loss were measured after different cycles. Following the initial triage, once the appropriate combinations of pore size/chemistry and MOF/lubricant combinations were determined (e.g. F-UiO-66 /Fomblin and A-UiO-67/silicone oil), a cumulative flow rate of 100 mL/h was used for long-term shedding test. Change in ∆θ was recorded at pre-defined time intervals from 30 minutes to 50 hours for these tests. The obtained results from different water shedding assessments are listed in Supplementary Table S2 and Table S3.

| **Lubricants** | **Initial wettability** | | **No. of water shedding cycles** |
| --- | --- | --- | --- |
|  | θ_Adv_ (°) | Δθ (°) |  |
| Si50 | 101±1 | 3±1 | 0 |
| Si500 | 104±1 | 4±2 | 0 |
| Krytox | 115±1 | 6±2 | 4 |
| Fomblin | 116±1 | 6±2 | 4 |

**Supplementary Table S2.** Dynamic contact angles and water shedding stability of SLIPS prepared from UiO-66 against four different lubricants including Si50, Si500, Krytox and Fomblin. The water shedding cycles numbers represent the maximum duration sustained with Δθ *≤* 10°. Each cycle used 50 mL of DI water.

| **SLIPS** | **Initial wettability** | | **No. of water shedding cycles** |
| --- | --- | --- | --- |
|  | θ_Adv_ (°) | Δθ (°) |  |
| UiO-66/Fomblin | 115±1 | 3±1 | 4 |
| UiO-67/Si500 | 104±1 | 4±2 | 3 |

**Supplementary Table S3.** Dynamic contact angles and water shedding stability of SLIPS prepared from UiO-66/Fomblin and UiO-67/Si500. The water shedding cycles numbers represent the maximum duration sustained with Δθ *≤* 10°. Each cycle used 50 mL of DI water.

| **Lubricants** | **NH_2_-UiO-66** | | **F-UiO-66** | | **A-UiO-66** | |
| --- | --- | --- | --- | --- | --- | --- |
| **Treatments** | **90° tilt** | **2000 RPM** | **90° tilt** | **2000 RPM** | **90° tilt** | **2000 RPM** |
| Si50 | 2.0 | 1.6 | 2.4 | 2.4 | 2.5 | 1.9 |
| Si500 | 2.7 | 2.4 | 4.5 | 3.2 | 3.9 | 2.7 |
| Fomblin | 5.1 | 4.1 | 5.4 | 4.5 | 3.3 | 2.9 |

**Supplementary Table S4.** The mass of lubricant (presented in terms of MOF weight) retained by different MOFs after tilting at 90° for one hour and spinning at 2000 RPM for one minute.

1. **Supplementary text**
   1. **Molecular dynamics and experimental study on lubricant intercalation**
      1. **Capillary pressure in the MOF pores**

The capillary pressure in the MOF pores (e.g. UiO-66) for silicone, Krytox, and Fomblin lubricants were calculated using the Young-Laplace equation[9],

$P_{\mathrm{cap}}=\frac{2 \sigma cos\theta}{R},$ (S1)

where $R$ is the radius of MOF aperture (~0.6 nm); $\theta$ is the contact angle which represents the surface tension between MOF and the lubricant; and $\sigma$ is the interfacial tension at the interface. The capillary pressure in the MOF pore was ~62 MPa for silicone oil ($\sigma$ = 21.0 mN/m), ~60 MPa for Krytox ($\sigma$ = 18 mN/m) and ~73 MPa for Fomblin ($\sigma$ = 22.0 mN/m).

- - 1. **Molecular dynamics simulations**

Molecular dynamics simulations were conducted with the Large-scale Atomic/Molecular Massively Parallel Simulator (LAMMPS)[10] to investigate the intercalation of lubricant chains inside MOF pores. Molecular structures of NH_2_-UiO-66 and lubricants, silicone and Fomblin, were created using Gaussian View program (Supplementary Figure S7A-C). The molecular structure of MOF comprised of tetrahedral pores with four metal clusters occupied by related organic linkers. The chain lengths for both the lubricants, silicone ([Si-O-(CH_3_)_2_]_20_) and Fombilin (CF_3_O(C_3_F_6_O_2_)_5_(CF_2_O)_5_CF_3_) were chosen to be similar. It is important to note that the sizes of the simulated molecular structures are smaller than the actual molecules due to the computational limits.

The flexible UFF force field was employed and LAMMPS input files were created using the *lammps_interface* program[11]. The molecular structures were optimized using PM7 method and their atomic charges were calculated using the Merz-Kollman electrostatic potential (ESP) charge method in the Gaussian program[12]. Periodic boundary conditions were applied with a 50 Å square box. The cut-off for van der Waals and Coulombic interactions were set 12.5 Å. A time step of 0.5 fs was used for the whole simulation. The particle–particle particle-mesh (pppm) simulation was used to calculate the long-range electrostatic interactions, with a relative precision of 10^-4^ (dimensionless). Each run was equilibrated for 0.6 ns at 300 K in the canonical (NVT) ensemble using the Nosé−Hoover thermostat[13, 14]. The change in potential energy for all runs was less than 0.5% during equilibration (between 0.5 ns and 0.6 ns). At the beginning of the MD simulation, the MOF and lubricants were all separated. Following equilibration, two Fomblin chains were adsorbed on a single MOF particle (Supplementary Figure S8A). In contrast, the silicone chains were partially separated from MOF (Supplementary Figure S8B). We also observed that the Fomblin chain could enter and perfectly fits into the octahedral MOF pore whereas silicone chain failed to penetrate through the aperture as shown in Supplementary Movie 1. The penetration of Fomblin chain inside the MOF pore (~6 Å) could be justified by its smaller diameter (~2.76 Å) in comparison to the larger diameter (~5.92 Å) of silicon molecular chain. The diameters of lubricant chains were calculated using PM7 method. However, given the bigger aperture size of UiO-67 (8 Å), silicone chain could be able to enter comfortably inside its pore (Figures 1H and 1J).

- - 1. **Samples preparation to study lubricant intercalation**

Pore size dependent intercalation of lubricant into the MOFs was confirmed experimentally. Two types of MOFs, namely, 1 g of UiO-66 and UiO-67 powders were infused with 2 g of Si500 in a 15 mL falcon tube. After infusion, the samples were kept for 12 hrs at 100 °C to replicate the SLIPS fabrication procedure. Samples were then cooled down to room temperature and then washed extensively (three times each) with chloroform and hexane to remove physically adsorbed lubricant. Washed samples were dried for 12 hrs under vacuum at 100 °C and then the dried powders were subjected to BET surface area characterization, ^13^C-NMR, and Raman spectroscopy to confirm the presence and intercalation of silicone chains inside MOF.

- 1. **Pore chemistry and host-guest interactions**
     1. **Surface energy for different functional groups**

The Lifshitz–van der Waals/acid–base (LW/AB) method can determine the surface energy of solids[15]. The surface energy can be divided into three components,

$\gamma=\gamma^{\mathrm{LW}}+2\sqrt{\gamma^{+}\gamma^{-}},$ (S2)

where $\gamma^{\mathrm{LW}}$, $\gamma^{+}$, and $\gamma^{-}$ denote the Lifshitz–van der Waals component, acid component, and base component, respectively. The relationship between solid and liquid is

$\gamma_{L}(1+cos\theta)=2(\sqrt{\gamma_{S}^{\mathrm{LW}}\gamma_{L}^{\mathrm{LW}}}+\sqrt{\gamma_{S}^{+}\gamma_{L}^{-}}+\sqrt{\gamma_{S}^{-}\gamma_{L}^{+}}),$ (S3)

where $\theta$ is the contact angle between solid and test liquid, and the subscripts S and L denote solid and liquid, respectively. The surface energy can be calculated by measuring the contact angles of three test liquids (with different$\gamma^{\mathrm{LW}}$, $\gamma^{+}$, and$\gamma^{-}$).

To compare the wetting and adhesion between different functional groups (used in MOF functionalisation) and liquids (water and lubricants), the surface energy was measured quantitatively using the LW/AB method. The test liquids used in this experiment were water, diiodomethane, and ethylene glycol. The obtained surface energy parameters are shown in Supplementary Table S5[16]. By solving a set of three simultaneous equations, surface energy of solid (silica wafer) modified with three different functional groups was calculated (Supplementary Table S6).

| **Liquids** | $\boldsymbol{\gamma}_{\mathbf{L}}$ | $\boldsymbol{\gamma}_{\mathbf{L}}^{\mathbf{LW}}$ | $\boldsymbol{\gamma}_{\mathbf{L}}^{\mathbf{+}}$ | $\boldsymbol{\gamma}_{\mathbf{L}}^{\mathbf{-}}$ |
| --- | --- | --- | --- | --- |
| Water | 72.8 | 21.8 | 25.5 | 25.5 |
| Diiodomethane | 50.8 | 50.8 | 0 | 0 |
| Ethylene glycol | 48 | 29 | 1.92 | 47 |

**Supplementary Table S5.** Surface energy parameters (mJ·m^−2^) of the testing liquids at 20 °C.

| **Solid** | **Water**  **(°)** | **Diiodomethane (°)** | **Ethylene glycol (°)** | $\boldsymbol{\gamma}_{\mathbf{S}}$ **(mJ·m^-2^)** |
| --- | --- | --- | --- | --- |
| Amino | 25.5 | 15.1 | 17.5 | 51.4 |
| Fluorine | 104.0 | 48.6 | 70.5 | 35.1 |
| Alkyl | 70.8 | 46.8 | 57.9 | 37.8 |

**Supplementary Table S6.** Contact angles of three functionalized surfaces and their calculated surface energies.

- - 1. **Noncovalent interactions using density functional theory (DFT)**

To study noncovalent interactions between different MOF linkers and lubricants, as a first step, the molecular structure optimisation was done using DFT at B3LYP/6-311G basis set in the Gaussian 16 software and confirmed by frequency analysis in the absence of imaginary frequencies. The results were then used to calculate the reduced density gradient (RDG) function[17] using the Multiwfn program[18] and plotted using VMD software[19]. The RDG function is based on the electron density (ρ) and its derivatives (sign(λ_2_)ρ). Sign(λ_2_) denotes the sign of the second eigenvalue (λ_2_) of the electron-density Hessian matrix, which is used to distinguish the attractive and repulsive interactions.

Noncovalent interactions were characterized by low density and reduced gradient values (Supplementary Figure S9). The orange isosurfaces represent strong mutual exclusion (repulsion) and nonbonded overlaps. The green discs represent van der Waals (vdW) interactions. Isosurfaces appear between Fomblin chain and hydrophobic linkers (functionalised with alkyl and fluorine chains) suggesting strong vdW interactions.

- - 1. **Electrostatic potential analysis**

Electrostatic interactions are different from the vdW interactions[20, 21]. Hence, it is necessary to distinguish between electrostatic and vdW interactions separately. The electrostatic interaction might be able to control molecular orientation in the lubricant.

Isopotential surfaces of electrostatic potential (ESP) were also analysed using the Multiwfn program[18] and plotted in VMD software[19] (Supplementary Figure S10). Both, silicone and Fomblin chains showed weak electropositive behaviour. The amino, alkyl, and fluorine functional groups showed electropositive, electroneutral and electronegative behaviours, respectively. The carboxyl groups and the amide bonds in the organic linkers were also found to be electronegative. The strong electrostatic interactions were observed between Fomblin and fluorine functionalised linkers (Supplementary Figure S10F). Silicone chains were observed to have electrostatic interactions with carboxyl functional groups and amide bond presented in functionalised linkers (Supplementary Figure S10B).

- - 1. **Interaction energy decomposition**

Symmetry-adapted perturbation theory (SAPT) [22], implemented in PSI4 code[23], was used to identify the relative contributions of electrostatic, dispersion, induction, and exchange forces. The latter two forces belong to van der Waals interactions. The bronze standard method (sSAPT0/jun-cc-pVDZ) was employed to calculate the intermolecular interaction energies between the linkers and lubricants. The relative contributions of different interaction energies are shown in Figure 2F.

- - 1. **Adhesion force measurements using atomic force microscopy**

Adhesion forces between different MOF particles (NH_2_-UiO-66, A-UiO-66, and F-UiO-66) and the lubricants (Si50 and Fomblin) were investigated using a Bruker Multimode 8 atomic force microscope (AFM) with PeakForce Quantitative Nanomechanics (PFQNM) in ‘tapping mode’. Supplementary Figure S11 shows a schematic of the AFM set-up used. Bruker RTESPA-525 AFM cantilevers with 8 nm tip radii were used. Spring constant of the cantilevers were estimated using thermal noise method for calibration[24]. First, a thin layer of lubricant was adsorbed to the AFM tip by approaching a small quantity of lubricant dispensed on mica substrate. A cantilever without lubricant layer was used as a control. Next, MOF particles were adhered to piezo scanner using double-sided carbon tapes and the unbounded or loosely attached particles were removed by blowing with pipette bulb. All AFM experiments were performed at room temperature (20 - 25 ºC).

To exclude the possible capillary rise/imbibition into the MOF pores, the excessive lubricant presented on AFM tip was removed by adsorption on separate MOF particles until the adhesion forces became constant. The time for each MOF absorption event was kept higher than the capillary imbibition time, which was estimated using modified Washburn’s equation[25] as follows

$t_{b}=\frac{2 L^{2} \mu}{R cos\theta\gamma K_{1}},$ (S4)

where t_b_, L, and R are the capillary imbibition time, length of capillary tube and radius, respectively. L and R were estimated based on the typical UiO-66 nanoparticle (200 nm) and pore size (~1 nm), respectively. The symbols $\mu$, $\gamma$ and $\theta$ denote dynamic viscosity, surface tension of the lubricant and the contact angle between the penetrating liquid and the solid, respectively. For Si50 oil, dynamic viscosity and surface tension are around 48 mN·s/m^2^ and 20 mN/m, respectively. The anomalous coefficient of nanotubes K_1_ is around 0.5 when R is 1 nm[25]. Therefore, the imbibition time for the lubricant infiltrating a MOF particle was estimate as 0.4 ms. By ensuring that the contact time between MOF particle and tip exceeded the calculated imbibition time, i.e. 0.4 ms, after multiple contacts with dry MOF nanoparticles, we ensured that ultimately only a thin layer of lubricant was left on the AFM tips, which is held together by van der Waals interactions between the tip and the oil. This allowed us to assess the interaction between the lubricant and MOF particle without any lubricant in its pores. The approach is similar to previously published studies on the measurement of adhesion forces between solid-liquid using AFM[26, 27]. The adhesion forces between bare AFM tip (without lubricant) and MOF particles were also measured for comparison.

Representative force-distance curves for Fomblin against NH_2_-UiO-66 and A-UiO-66 are shown in Supplementary Figure S12 and Figure 2G. Tip approach velocity and retraction velocity were kept at ~0.1 µm/s during all the measurements. The ramp distance was 750 nm, and the maximum normal force applied on the mica surface was kept constant (750 nN) using a relative trigger threshold. Neither the ramp distance nor the maximum applied force were considered independent variables. The presented adhesion forces in Figure 2H are the average pull-off force measured from 8 individual force-distance curves.

Force-distance curves were analysed using Bruker NanoScope Analysis 9.1 software. Briefly, the adhesion force was calculated as the difference between the baseline (0 mN/m force) of the retract dataset and the largest negative value (pull-off point). Adhesion forces from each tip-MOF combination were averaged, and the standard deviation was calculated. Adhesion forces were normalised by 2πR, where R is the tip radius. Forces are expressed in units of tension per unit length (N/m) which is equivalent, numerically and dimensionally, to the interaction free energy (J/m^2^).

As shown in Figure 2H, adhesion forces decrease in the order F-UiO-66/Si50 > A-UiO-66/Si50 > A-UiO-66/Fomblin > F-UiO-66/Fomblin > NH_2_-UiO-66/Si50 > NH_2_-UiO-66/Fomblin. It is important to note that there might be some contribution of hydrodynamic and capillary forces in the measured adhesion values. But at the used low tip velocity of 0.1 µm/s, the contribution of hydrodynamic forces expected to be negligible[28, 29]. Despite the removal of excessive lubricant from AFM tips through multiple adsorption on bare MOF particles, there might be some capillary forces presented. Overall, the adhesion forces F_ad_ consist of the electrostatic force F_elec_, the van der Waals force F_vdW_, and the capillary force F_cap_ in the presence of lubricant meniscus[30]:

$F_{\mathrm{ad}}=F_{\mathrm{elec}}+F_{\mathrm{vdW}}+F_{\mathrm{cap}},$ (S5)

The capillary force F_cap_ between tip with radius R and a MOF particle can be calculated using:

$F_{\mathrm{cap}}=4\pi R cos\theta\gamma,$ (S6)

For silicone oil, the maximum value of $F_{\mathrm{cap}}$ is calculated around 2 nN and its normalised value (2πR) is 0.04 N/m. This value is equivalent to the adhesion forces measured between hydrophilic NH_2_-UiO-66 and silicone oil using AFM (~0.06 N/m) suggesting that the other interactions ($F_{\mathrm{elec}}$ and $F_{\mathrm{vdW}}$) are negligible. However, the adhesion forces we measured for silicone oil and hydrophobic MOFs using AFM were much larger, >3 N/m and can be interpreted as non-covalent interactions with negligible contribution of capillary and hydrodynamic forces.

- 1. **Lubricant replenishment and retention**
     1. **Lubricant recovery**

The reticulated porosity of MOFs enables local recovery/replenishment of lubricant from neighbouring MOF pores. The lubricant replenishment/recovery of SLIPS was thus studied alongside water shedding tests. For SLIPS with sprayed nanoparticles, the inter-particle gaps can also serve as source of lubricant replenishment (see Supplementary Figure S23). The bright, yellow spot enabled the presence or absence of lubricant to be discerned readily. Supplementary Figure S23 shows the snapshots from videos of the top-view of SLIPS recorded using a high-resolution camera. The boundary of the bare MOF surface (covering the path of the sliding droplets) can be easily distinguished from the lubricant-coated surface. Lubricant recovery over time was carefully assessed from these videos.

- - 1. **Theoretical loading capacity of UiO-66**

Theoretical loading capacity (L_F_) of UiO-66 (0.72 g/g) for Fomblin was estimated by assuming that the volume occupancy of lubricant molecules inside MOF pores is similar to that of small molecules,

$L_{F}=V_{\mathrm{MOF}}\rho_{F}$ (S7)

where the specific volume ($V_{\mathrm{MOF}}$) of UiO-66 pore[31] is 0.379 cm^3^ g^−1^  and the density ${(\rho}_{F}$) of Fomblin is 1.9 g mL^−1^.

- 1. **Robustness tests**
     1. **Comparison against state-of-the-art SLIPS**

We benchmarked the duration of lubricant retention on the surface and the maximum dispensed liquid (water) volume (see Figure 4J) and compared our results against control SLIPS. Δθ <10° for water droplets was used as a metric to assess the retention of surface liquid repellence (slipperiness) over time.

To the best of our knowledge, only handful of studies have assessed/reported similar durability studies on SLIPS under continuous water shedding and most of the surfaces are unable to survive for more than couple of hours. The volume of water droplets used in previous studies ranged from around 10 µL to 20 µL, and the test surface inclination angle ranged from 7° to 30°. A droplet dripping frequency of 1 s^-1^ was assumed in order to compare the duration data of the above studies with our own results. Each data point in Figure 4J shows the stability of different combinations of surfaces and lubricants during continuous water shedding experiments:

- Electrosprayed rubber: Vulcanized silicone rubber prepared using a one-step electrospray method infiltrated with a perfluoropolyether lubricant[32],
- ZnO nanowire: Zinc oxide nanowire arrays prepared by chemical bath deposition (CBD) and infused with silicone oil[33],
- CuO nanoparticles: Needle-like copper oxide nanoparticles (synthesized using CBD method) coated with polymethylsiloxane (PDMS) and infused with silicone oil[34],
- Si nanotubes: Silicon nanotube array fabricated by spacer defined intrinsic multiple patterning with lubricant Krytox 1525[35],
- Fluoro PDA: Polydopamine (PDA) layer grafted with the fluorinated poly(hexafluorobutyl methacrylate-co-glycidyl methacrylate) polymers and infused with perfluorinated lubricant[36].
  - 1. **Jet impact test**

A bespoke set-up fitted with nitrogen gas cylinder connected to an electronic pressure valve was used for jet impact test[7]. Diameter of water jet was 2.5 mm and the maximum back-pressure was $\sim$12 bar. The speed of jet was calculated from motion of the piston and related parameters including nozzle diameter using following the continuity equation:

$\pi d_{s}^{2}{\Delta h}/4=\pi d_{n}^{2}V_{\mathrm{jet}}{\Delta t}/4$ (S8)

where Δt is the elapsed time, Δh is the distance, 𝑑_𝑠_ is the cylinder diameter, 𝑑_𝑛_ the nozzle (jet) diameter and V_jet_ is the jet speed.

The maximum jet speed reached in our experiments (~35 m/s) was determined by averaging the maximum speeds in different tests. The corresponding liquid Weber number (𝑊𝑒_𝑙_=𝜌$V^{2}$𝑑_𝑛_/𝛾) calculated for the 2.5-mm jet was ~42,500 (Supplementary movie 4). The surface showed no signs of damage or permanent liquid impalement even after at least 3 jet impacts on the same spot. The liquid impalement resistance of surfaces was assessed using a droplet mobility test; a droplet was placed at the centre of the impact location following jet (Figure 5C and supplementary Figure S27 and Supplementary movies 4 and 5) and checked for pinning or sliding.

- - 1. **Tape peel and pencil scratch tests**

To investigate the adhesion of MOF (NH_2_-UiO-66) on substrates, a high-adhesion, pressure-sensitive tape (3M VHB™ Tape 5952, with an adhesive strength of 3,900 N/m) was used. The tape was applied to the coatings, which were laid flat, by rolling a 2-kilogram steel roller to ensure uniform contact. After waiting for 60 seconds, the tape was peeled off by hand. The test was repeated for 50 cycles, using a fresh strip of tape for each cycle. After tape peel cycles, no significant change in morphology was found for the MOF coatings on aluminium (Supplementary Figure S29). This strong adhesion was due to the ester bonds between carboxyl groups of the linker and the hydroxyl groups of the aluminium substrate (Supplementary Figure S28).

Next, a standard pencil hardness test (ASTM D 3363) was conducted to test the resistance of the MOF coatings to scratches. A pencil with hardness level of 5H was used. The pencil lead were rubbed on sand paper to achieve a flat cross-section before the test. The lead was then pushed horizontally laid MOF surface at ~ 45° tilt angle. After the test, integrity of the surface was checked by SEM (Supplementary Figure S30).

- - 1. **Tribological measurements**

To investigate the robustness of SLIPS under jet impact, both macroscopic tribological and nanotribological properties were measured using a tribometer and an AFM in lateral force microscopy (LFM) mode, respectively[37, 38]. A universal reciprocating macro-tribometer (Model: UMT-3MT, Center for Tribology Co., Inc. (CERT), America) with ball-on-disk mode was selected to measure the friction coefficient of SLIPS samples at ambient temperature before (freshly infused) and after high-speed water jet impact. A hardened bearing steel 52100 ball with a diameter of 6 mm was installed in the clamp of the tribometer as the upper sample. The load was set to 2 N during the sliding shearing test. The corresponding sliding speed and time were adjusted to 38 mm/s and 30 min, respectively. The reciprocating length was 10 mm. All tests were repeated at least thrice at room temperature and relative humidity of 40–60 %. The time-series results and the averaged values were presented in Supplementary Figure S31 and Figure 5D, respectively.

Next, the nanotribological properties were measured using a LFM mode in an AFM (Bruker Multimode 8). The smoother glass substrates were used to fabricate SLIPS for consistent and reliable results. LFM was performed in contact mode using a CONTV-A tip with a spring constant of 0.19 N/m and a deflection sensitivity of 165 nm/V. Friction was measured under constant load using a 90° scan angle and the tip was repeatedly scanned along the x-axis over the sample surface. The scan length was 500 nm and the frequency was 1 Hz. The friction signal was the average signal of the retrace scan. The normal load (31.9 nN) was controlled by changing the set-point voltage, which was 357.79 mV for all samples. The frictional signal are presented in Supplementary Figure S32 and Figure 5D.

- 1. **Applications**
     1. **Ice adhesion measurement**

A custom bench-top icing chamber was used for the ice adhesion experiments as reported in our previously published work[5, 39]. The chamber comprised of a transparent, double-walled container and a cooling base with external dimensions of 30 cm × 20 cm × 18 cm and a 10 mm air gap between its external and internal walls. The 5 mm walls were made of thermally insulating Perspex and the air gap was evacuated to improve the insulation. The base contains an aluminium frame, a base plate, a compact heat exchanger (P1805368, UK Exchangers), 4 axial fans below the heat exchanger (ARX CeraDyna Series, RS Components), a rotary aluminium stage, a plastic shaft and two Peltier cooling modules between the aluminium stage and the heat exchanger. 4 K-type thermocouples (HH506RA) were used to measure chamber temperature. Temperature was controlled using a refrigeration unit (FP50-HL Refrigerated/Heating Circulator, Julabo) with bath fluids (H5, Julabo) connected to the compact heat exchanger. A 3-pin humidity sensor (HIH-4000-001, RS Components) was used to measure humidity. The data acquisition system (DAQ) included a compact DAQ chassis (cDAQ-9174, National Instruments) with a temperature module (NI9213), a voltage module (NI-9263) and an analogue module (NI-9209). An extension rod connected to a force gauge (M4-50, MARK-10) was mounted on a custom-made driving system equipped with a stepper motor (17HS19-2004S1). LABVIEW software was used to operate and record the forces and determine the corresponding adhesion presented in Figures 5E-F and Supplementary Figure S33.

- - 1. **Dynamic anti-fouling and anti-biofouling tests**

*Flow chamber design:* A closed-loop flow chamber with dimensions of 255 mm $\times$ 205 mm $\times$205 mm ($w \times h \times d$) was 3D printed from polylactic acid (PLA) polymer (Supplementary Figure S34) using PLA based melted extrusion modelling (Tiertime). The nozzle diameter for printing was 1.75 mm and the geometry of chamber was developed on Autodesk Fusion 360 software. Inlet and outlet diameters were 20 mm. The chamber was connected to submersible pump (BARST, 350 L/h) and used for dynamic anti-fouling and anti-biofouling tests as explained below.

*Anti-fouling:* The fouling experiments were performed at dynamic conditions at 25 °C for 4 days (96 hrs). A supersaturated solution of CaCl_2_ (0.222 g, 1 mM) and Na_2_HCO_3_ (0.336 g, 2 mM) in 2 L of DI water was stirred for around 60 minutes to obtain a clear solution. Then the surfaces were fixed in vertical position along the chamber wall using commercial Blu Tac® adhesive. A submersible pump was used to drive the supersaturated solution of CaCl_2_ and Na_2_HCO_3_ in a closed loop through the chamber at a flow rate of 350 L/h. Then the surfaces were characterised for crystal deposition/growth after 96 hrs using SEM with Energy Dispersive Spectroscopy (EDS) (Supplementary Figures S35 and S36). The obtained high-resolution images were processed using imageJ for crystal growth quantification.

*Anti-biofouling:* Gram positive S. aureus bacteria (8325-4 strain) was used for anti-biofouling testing at a dynamic flow condition. A single colony was extracted from the glycerol stock of bacteria on LB agar plate (prepared using Lennox L Broth Base from Invitrogen^TM^) and incubated for 12 hrs in 10 mL of LB broth at 37 °C to prepare primary culture. After growth, bacteria containing broth was diluted to OD_600_=0.3 which was measured with a UV-vis Spectrophotometer (Orion™ AquaMate). 500 mL of bacterial culture flowed through the chamber containing control and SLIPS surfaces and the temperature was maintained at 37 °C in the incubator. The flow rate was 350 L/h and wall shear stress was calculated as 0.0085 Pa. Bacteria adhesion and biofilm behaviours were checked (after 6 hrs and 50 hrs) under flow condition simulating urinary catheters. The culture inside chamber was refreshed every 6 hrs to maintain the bacteria concentration level and avoid overloading. SEM (EVO, ZEISS) with voltage 5 kV and current 100 pA was used to image samples after air-drying for 1 hr. The high-resolution SEM images were processed in ImageJ software to quantitatively analyse the surface coverage with bacteria at five random locations.

*Wall shear stress in chamber:* For real-world applications, such as urinary catheters[40] and heat exchanger tubes[41], liquid containing contaminants, microbes or minerals flow across the surfaces, providing continuous shear that may also deteriorate the SLIPS or the retaining micro/nanoporous substrate structure. To evaluate our rational SLIPS, a closed-loop chamber was designed to test the surfaces under dynamic/flow conditions. The flow Reynolds number was calculated by

| $\mathrm{Re}=\frac{VL\rho}{\mu},$ | (S9) |
| --- | --- |

where average velocity of flow V is 0.06 m/s, width of the chamber L is 0.04 m. Density of LB broth or supersaturated solution of minerals ρ is around 1000 kg/m^3^.Viscosity of LB broth μ is 7x10^-4^ Pa·s. This yields Re=3428, which suggests that our chamber flow should be transitional.

To a first approximation, we can use Darcy–Weisbach equation, to estimate the mean wall shear stress τ inside the chamber channel[42]:

| $\tau=\frac{8}{\mathrm{Re}}\rho\left\langle V \right\rangle^{2}$. | (S10) |
| --- | --- |

The $\tau$ inside chamber under maximum flow rate (350 L/h) is calculated 8.5 mPa which is comparable to wall shear stresses in medical catheters[40].

1. **Supplementary Figures**


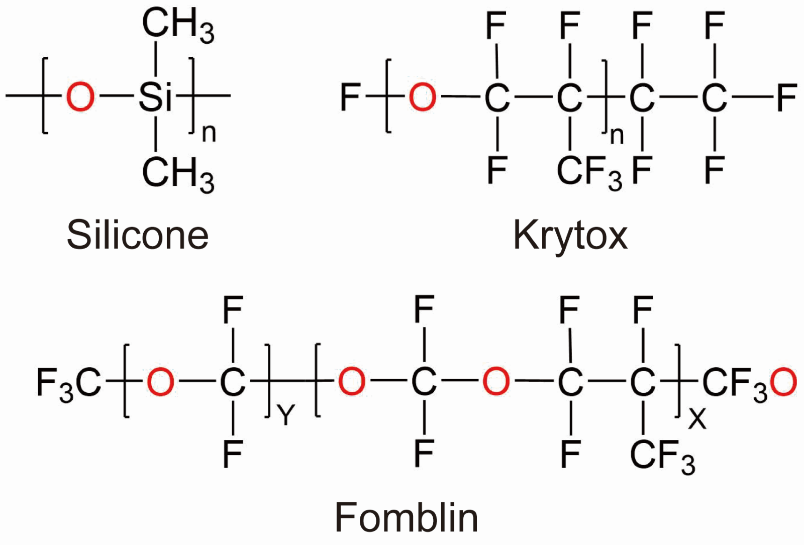


**Supplementary Figure S1.** Chemical structures of the lubricants, silicone, Fomblin, and Krytox.


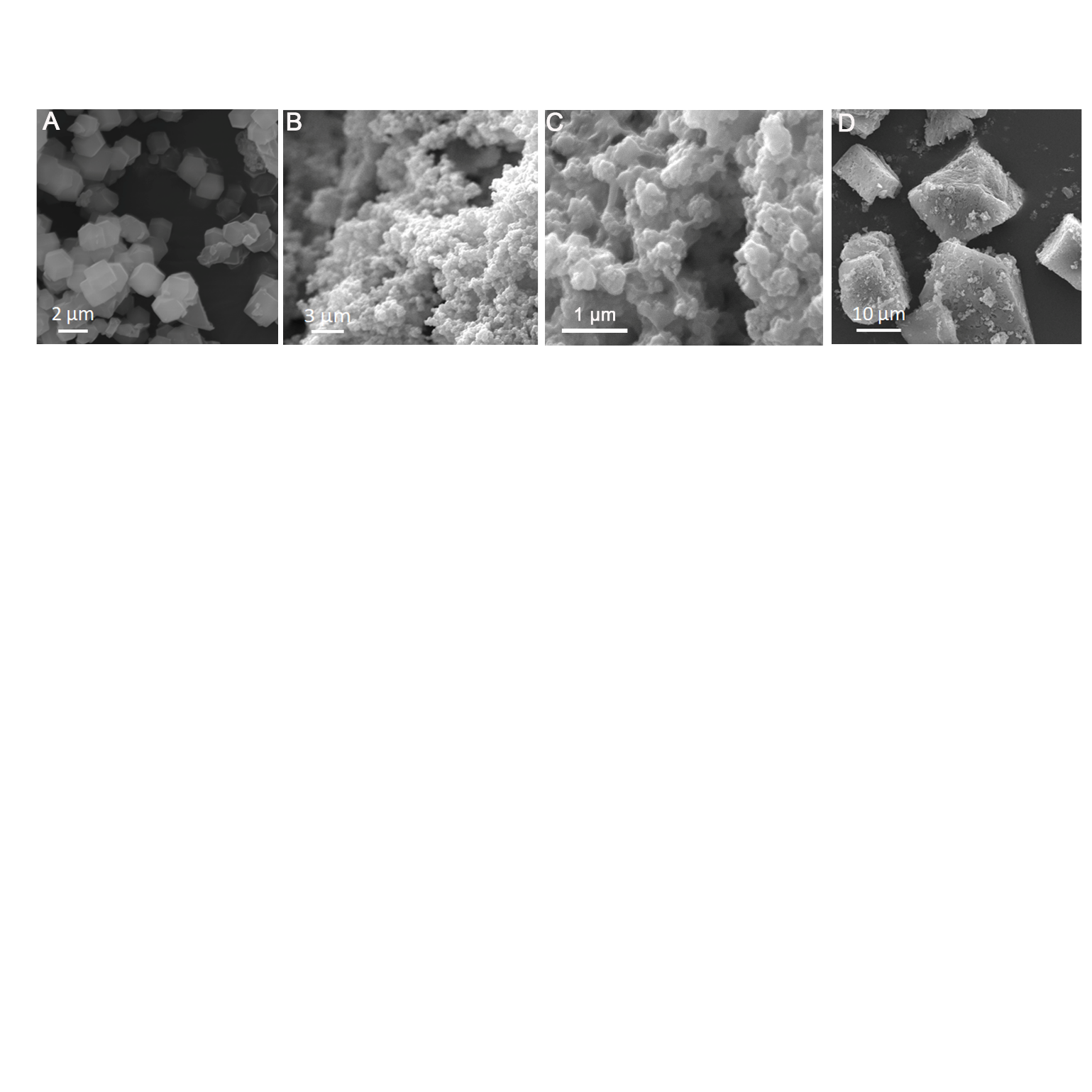


**Supplementary Figure S2.** SEM images of (A) ZIF-8, (B) NH_2_-UiO-66, (C) NH_2_-UiO-67, and (D) NH_2_-MOF-5 particles. A typical morphology was observed for each MOF type.


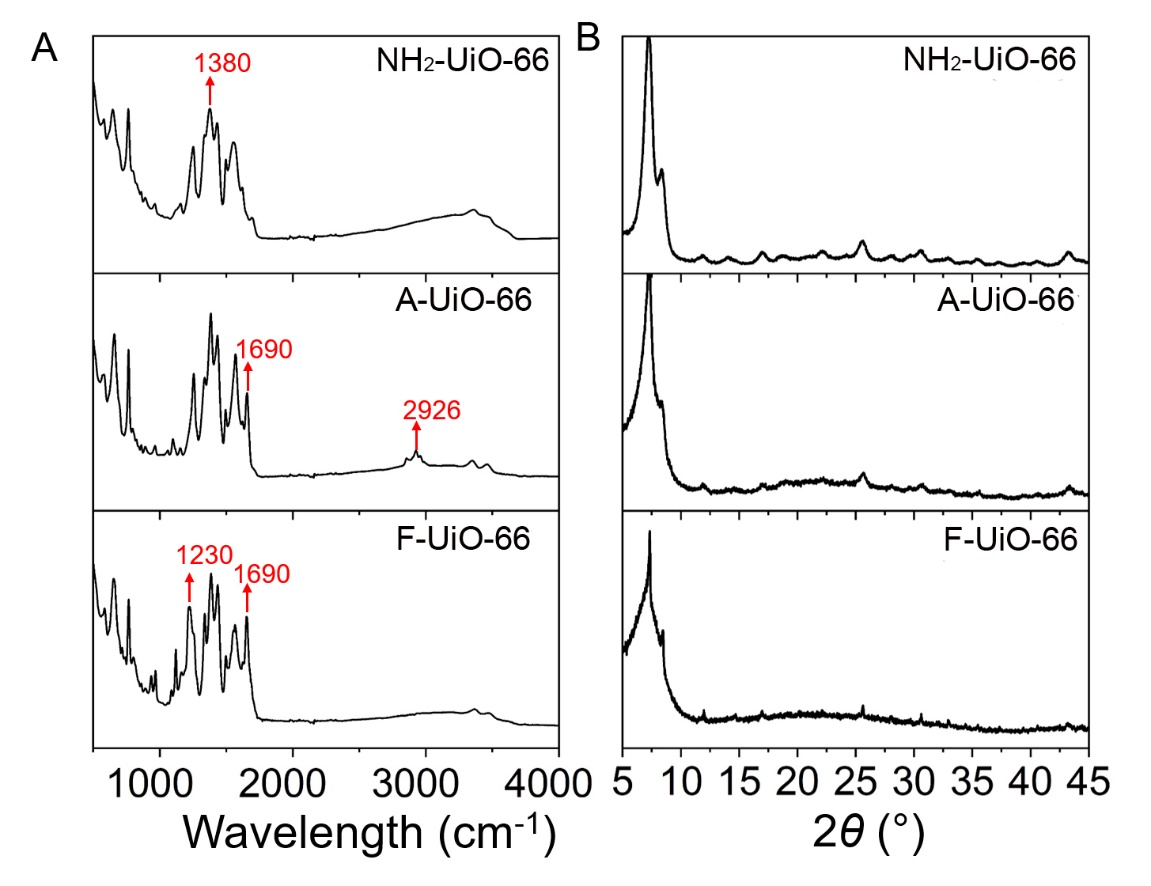


**Supplementary Figure S3.** (A) FTIR and (B) PXRD graphs of NH_2_-UiO-66 and functionalised MOFs (A-UiO-66 and F-UiO-66) confirming the successful synthesis and post-synthetic modifications. The red marked peaks at 1380 cm^-1^ in all three graphs indicate the presence of C-OH bond in the MOF linker. Peaks at 1690 cm^-1^ indicate C=O stretching, confirming the covalent linkage of flouro (in F-UiO-66) and alkyl (in A-UiO-66) chains to the amino functional group of hydrophilic NH_2_-UiO-66. Characteristic peaks at 1230 cm^-1^ and 2926 cm^-1^ for C-F and C-H stretching also confirmed the successful post-synthetic modifications. Retention of the characterstic peaks of UiO-66 crystalline structure in functionalised MOFs suggested that the molecular arrangements remained unaltered.


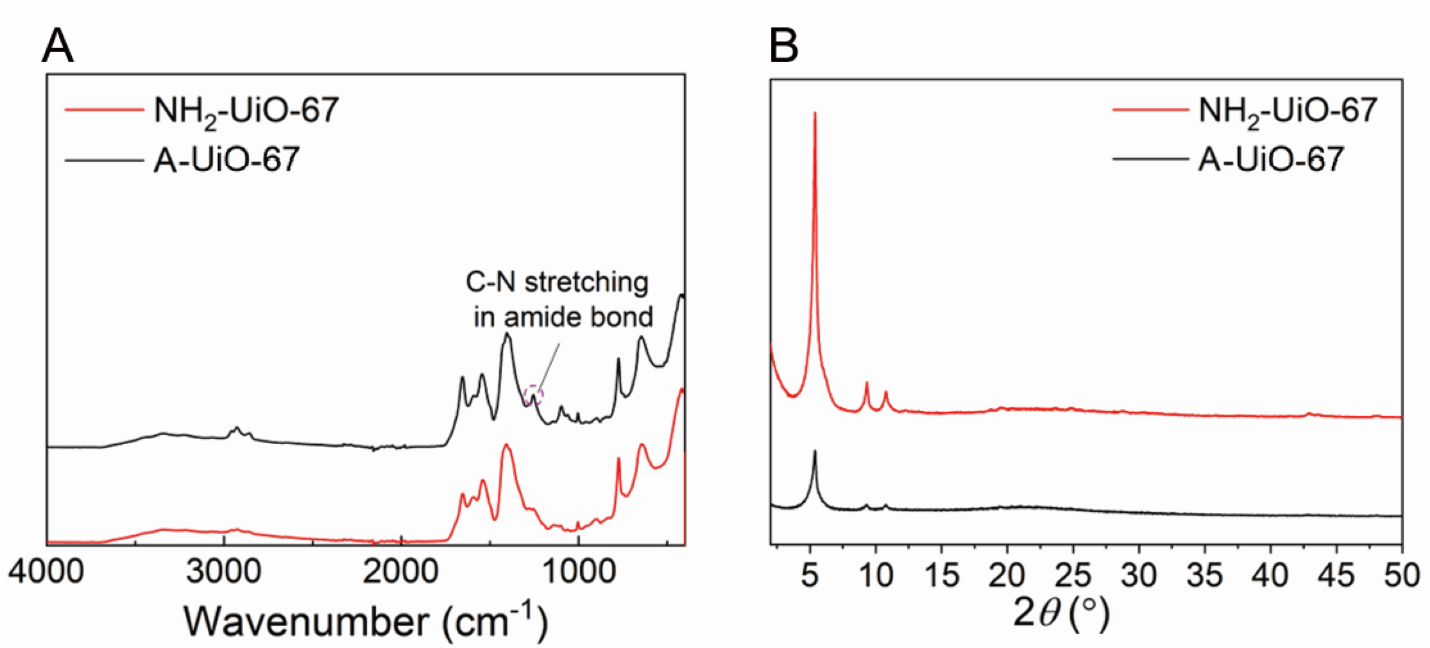


**Supplementary Figure S4.** (A) FTIR and (B) PXRD graphs of NH_2_-UiO-67 and alkyl functionalised A-UiO-67 confirming the successful synthesis and post-synthetic modifications. Presence of C-N stretching confirmed the sucesful linkage of alkyl chains to the MOF linker.


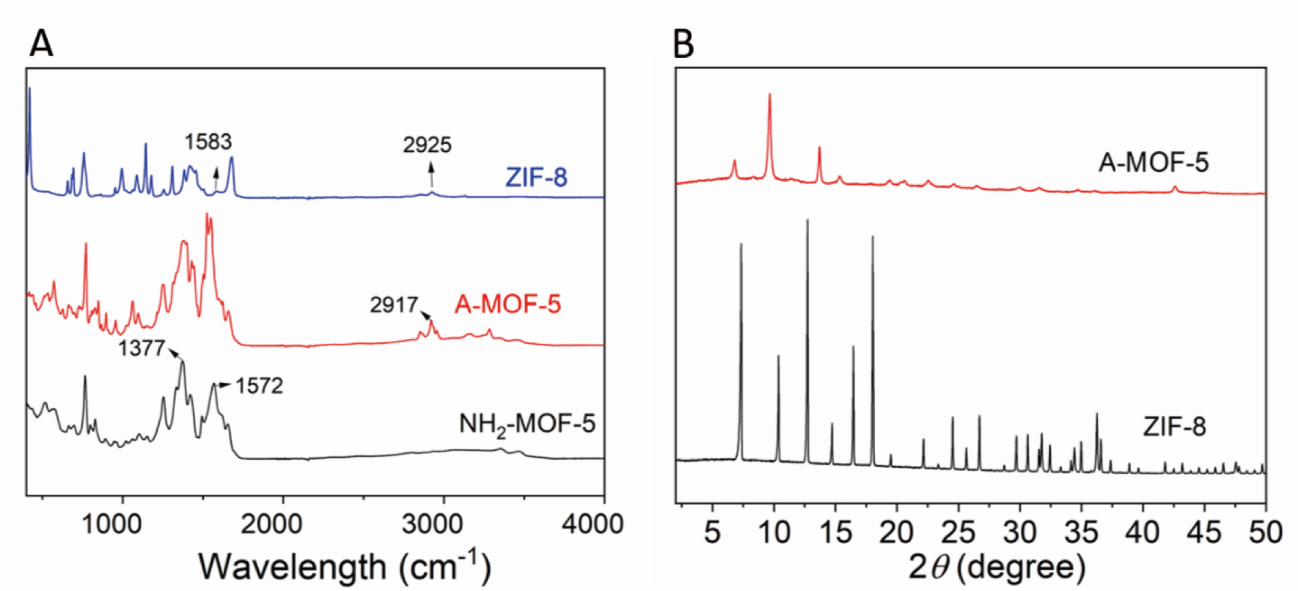


**Supplementary Figure S5.** (A) FTIR and (B) PXRD graphs of ZIF-8, NH_2_-MOF-5, and alkyl functionalised A-MOF-5 confirming the successful synthesis and post-synthetic modifications. C=N and C-H stretching peaks at 1583 cm^−1^ and 2925 cm^−1^, respectively confirmed the chemical structure of ZIF-8. Specific FTIR peaks at 1377 cm^−1^ and 1572 cm^−1^ attributed to symmetric and asymmetric stretching of C-O bonds in benzene ring confirmed the MOF-5 structure. The post-synthetic functionalisation of MOF-5 with alkyl chains was confirmed by C-N stretching peak at 2917 cm^−1^. PXRD spectra showed typical peaks of ZIF-8 and alkyl functionalised MOF-5 structures.


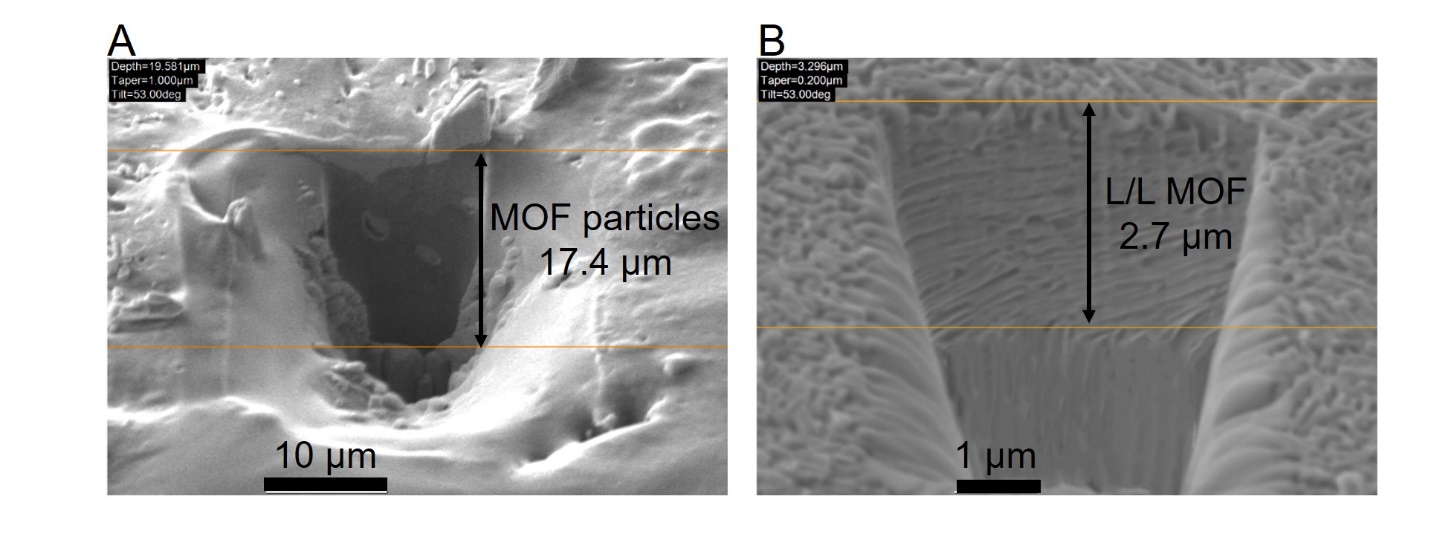


**Supplementary Figure S6.** Thickness measurement using Focused Ion Beam-Scanning Electron Microscopy (FIB-SEM). The thickness of (A) the sprayed and (B) L/L MOF samples (F-UiO-66) are estimated to be around ~20 µm and ~3 µm, respectively.


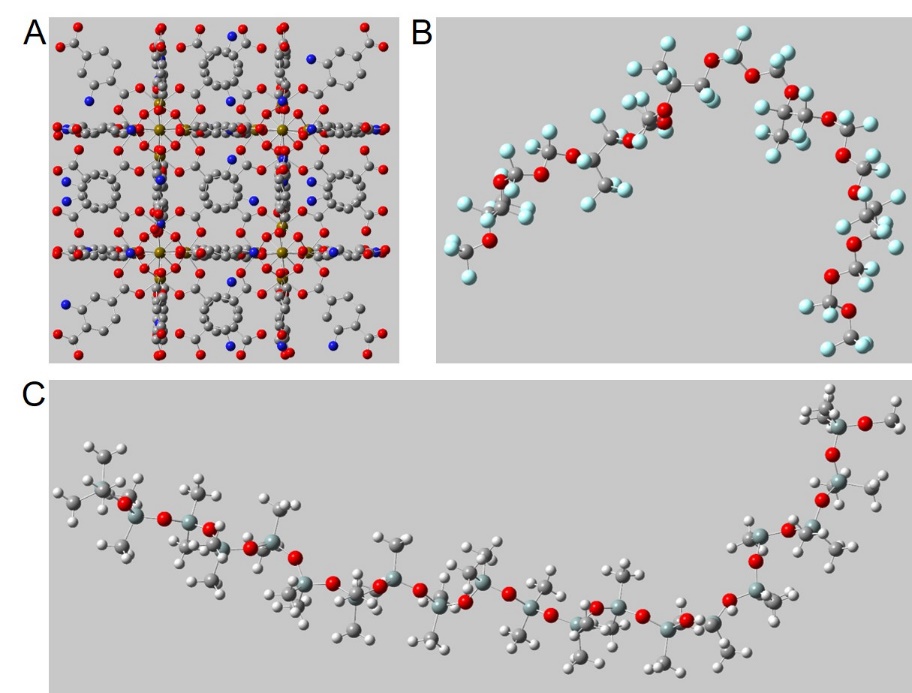


**Supplementary Figure S7.** Optimised molecular structures of MOF and lubricants. (A) NH_2_-UiO-66, (B) Fomblin chain, and (C) Silicone chain. Hydrogen atoms bonded to MOFs have been omitted for clarity.


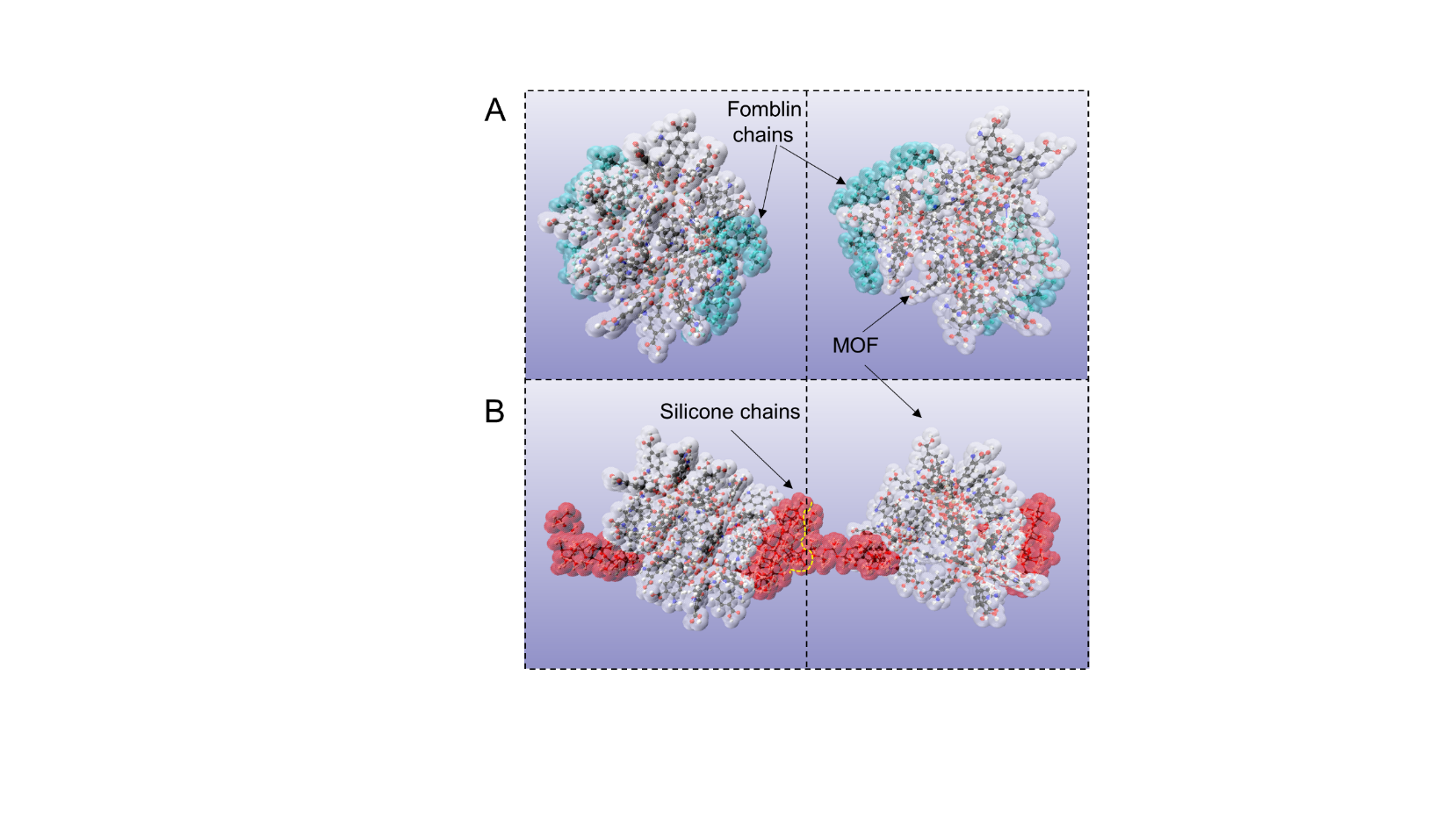


**Supplementary Figure S8.** Intercalation of lubricant chains in UiO-66 after NVT equilibration for 0.5 ns at 300K. (A) UiO-66 and Fomblin chains. (B) UiO-66 and silicone chains. (The translucent white blobs represent UiO-66, the translucent cyan surface represents Fomblin and the translucent red surface represents silicone lubricant. Fomblin chain is observed adhered to UiO-66 due to strong interactions. In contrast, silicone chains struggled to adhere to the UiO-66 as the interactions between two silicone chains surpassed their interactions with MOFs. Yellow dotted line shows the boundary of two silicone chains).


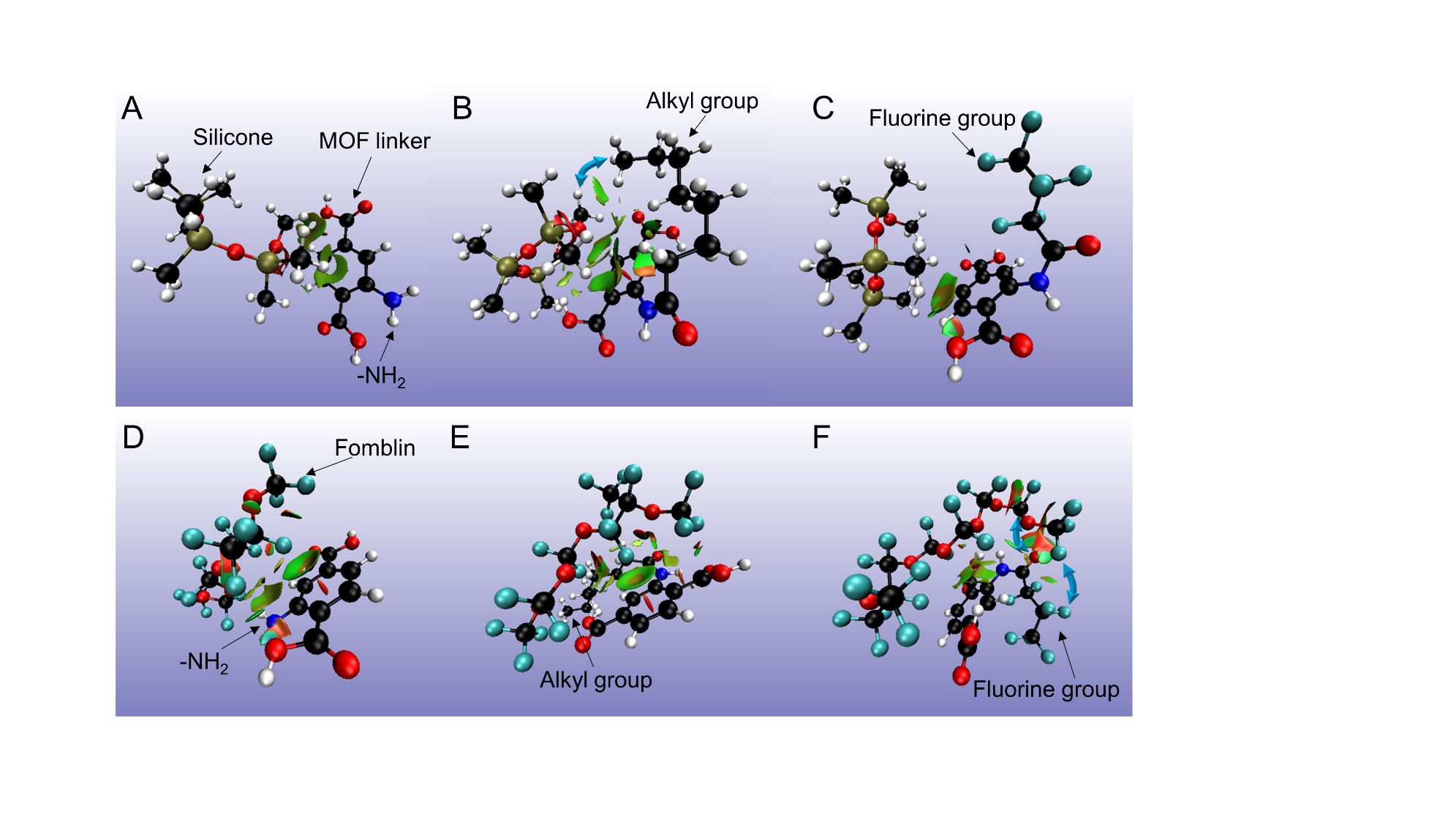


**Supplementary Figure S9.** Gradient isosurfaces for (A) amino functionalised linker and silicone chain, (B) alkyl functionalised linker and silicone chain, and (C) fluorine functionalised linker with silicone chain. (D) Amino functionalised linker and Fomblin chain, (E) alkyl functionalised linker and Fomblin chain, and (F) fluorine functionalised linker with Fomblin chain. Coloured balls represent different atoms: white is H, red is O, blue is N, black is C, cyan is F, olive is Si. The two-way blue arrows indicate the interaction between lubricants and functional groups.


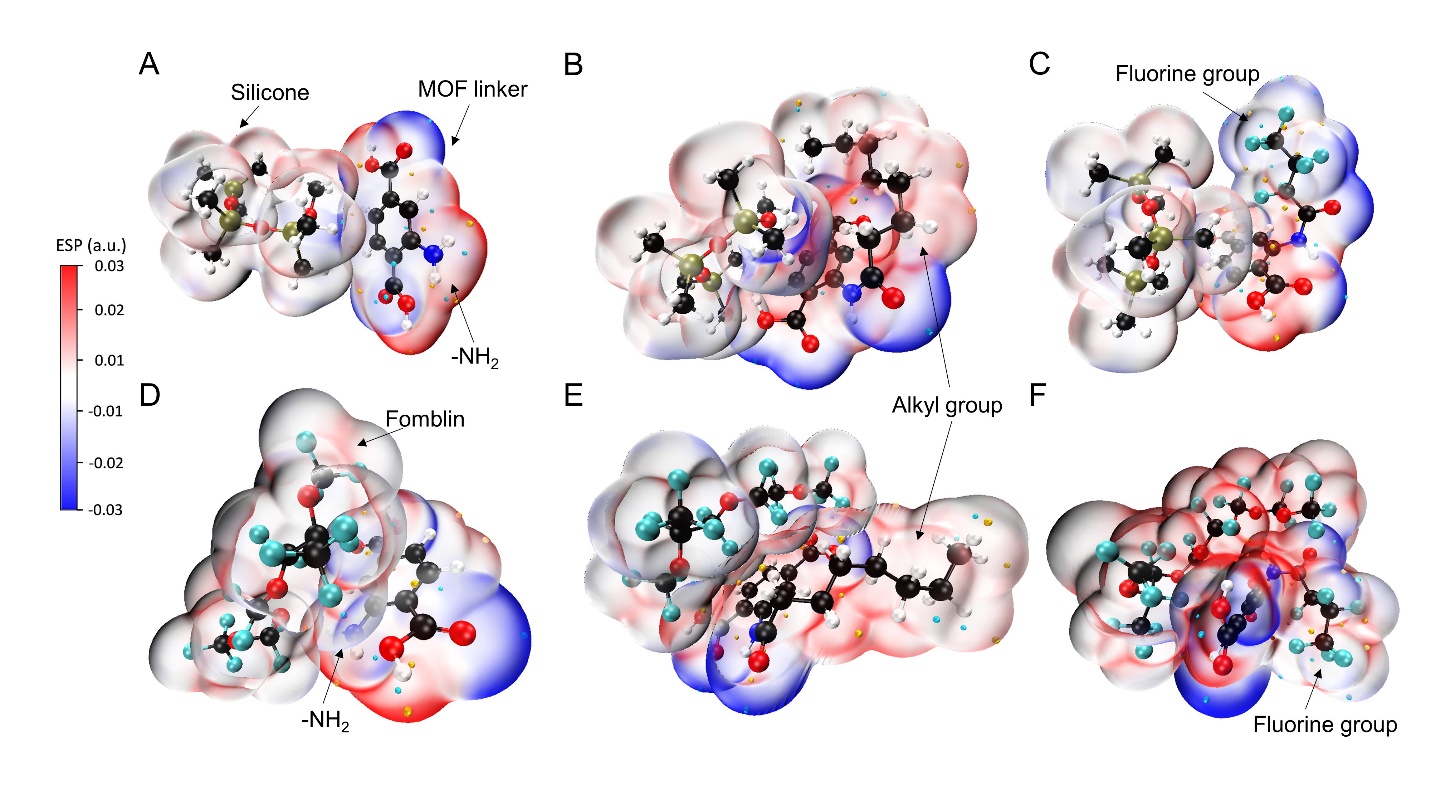


**Supplementary Figure S10.** Electrostatic potential (ESP) plots of (A) amino functionalised linker and silicone chain, (B) alkyl functionalised linker and silicone chain, and (C) fluorine functionalised linker with silicone chain, (D) amino functionalised linker and Fomblin chain, (E) alkyl functionalised linker and Fomblin chain, and (F) fluorine functionalised linker with Fomblin chain. Yellow dots represent electrostatic potential maxima and blue dots represents electrostatic potential minima.


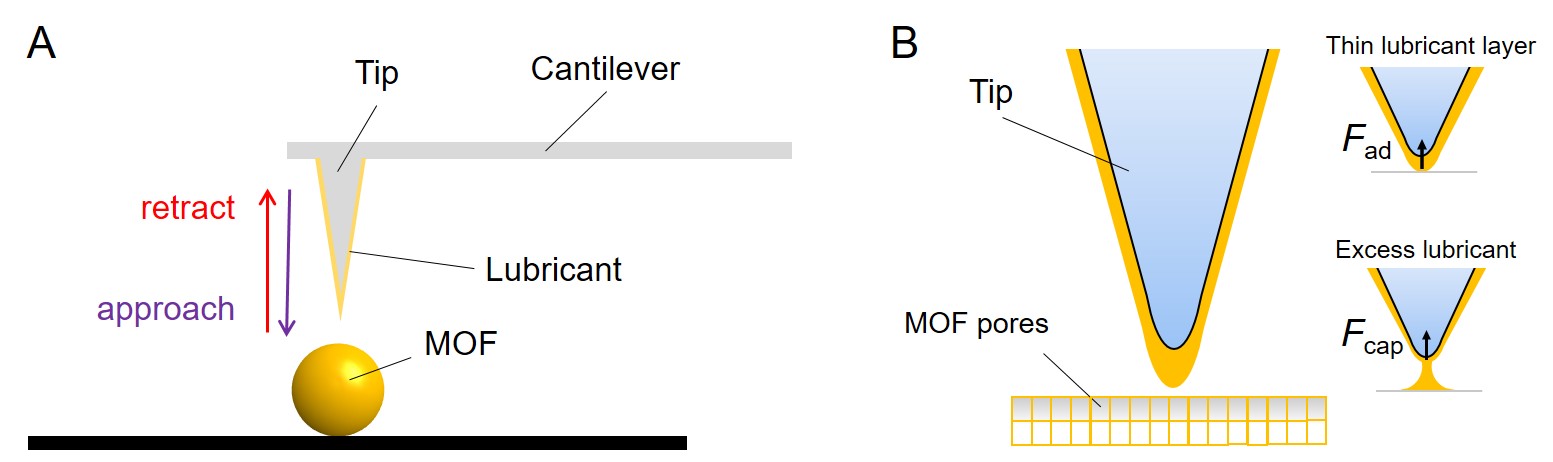


**Supplementary Figure S11.** (A) Schematic illustration of AFM adhesion force measurement and (B) contact interface between tip, lubricant, and MOF particle.


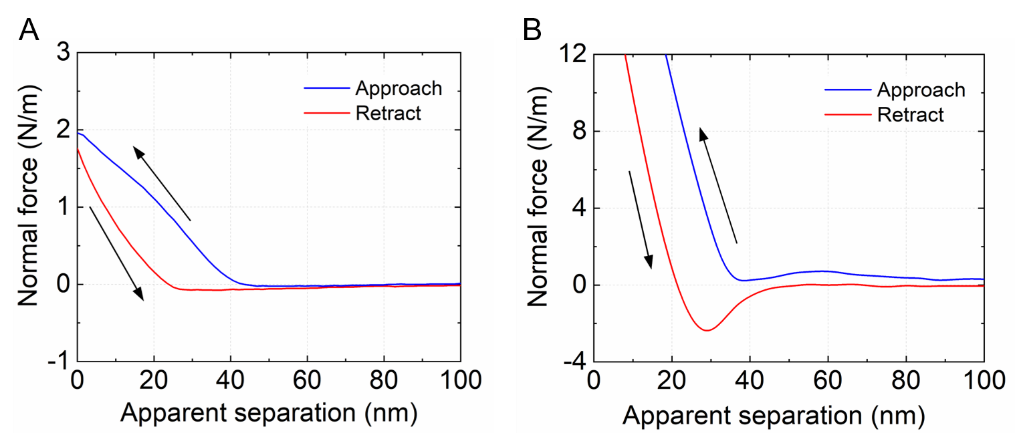


**Supplementary Figure S12.** Representative force-distance curves of (A) NH_2_-UiO-66 and Fomblin, and (B) A-UiO-66 and Fomblin. The approach and retract velocities were both 0.1 µm/s. The adhesion force was taken to be the difference between the baseline (0 N/m) and the largest negative normal force in the retract dataset.


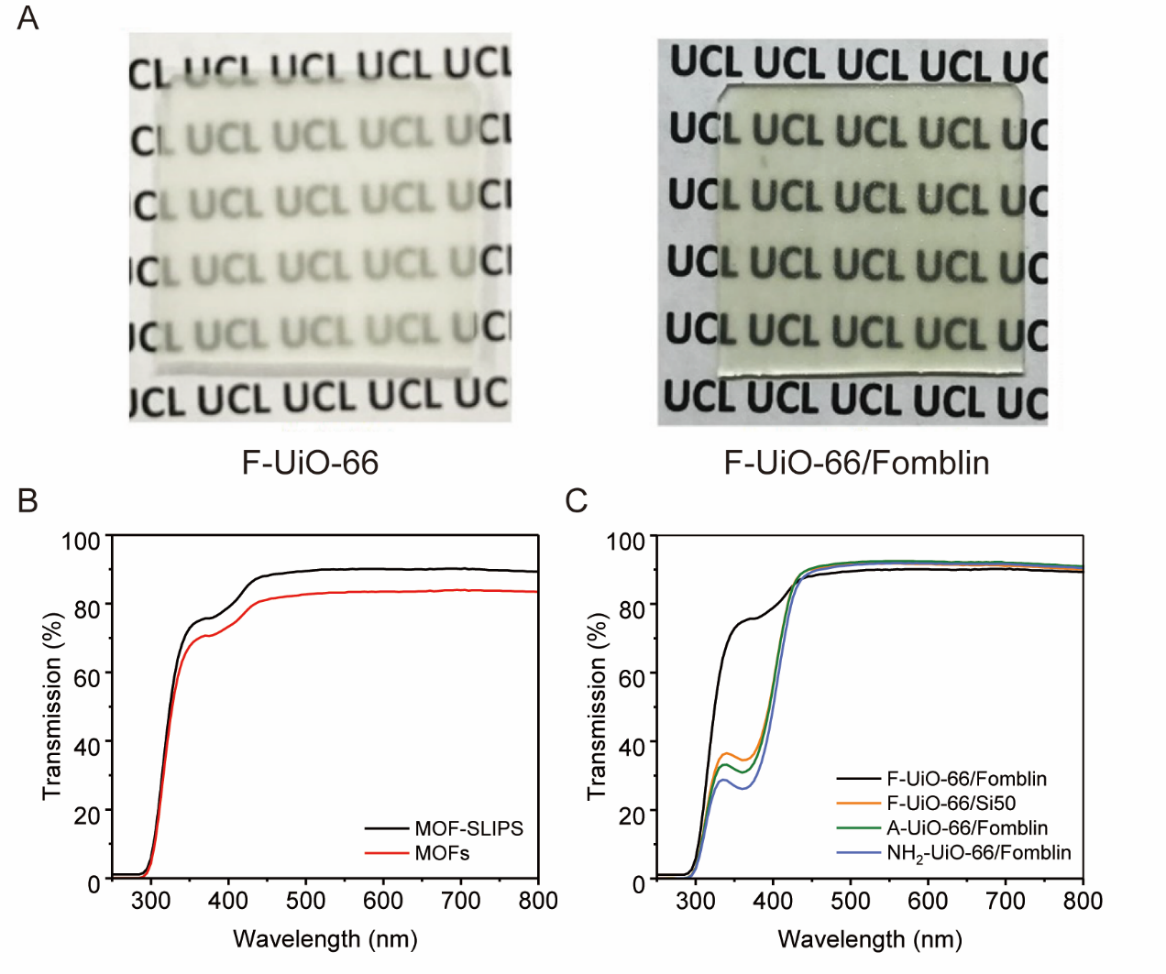


**Supplementary Figure S13.** **Transparency of the surfaces.** (A) Comparative photographs of a glass substrate sprayed with functionalised modified MOF (F-UiO-66) particles (left) and the surface after infusing lubricant (right). Reduction in scattering with oil infusion is clear from the pictures. (B) Transmittance of the dry functionalized MOFs (F-UiO-66) and corresponding SLIPS (F-UiO-66/Fomblin) measured using UV-Vis spectroscopy. (C) Transmittance of SLIPS with different functionalized MOFs and lubricants.


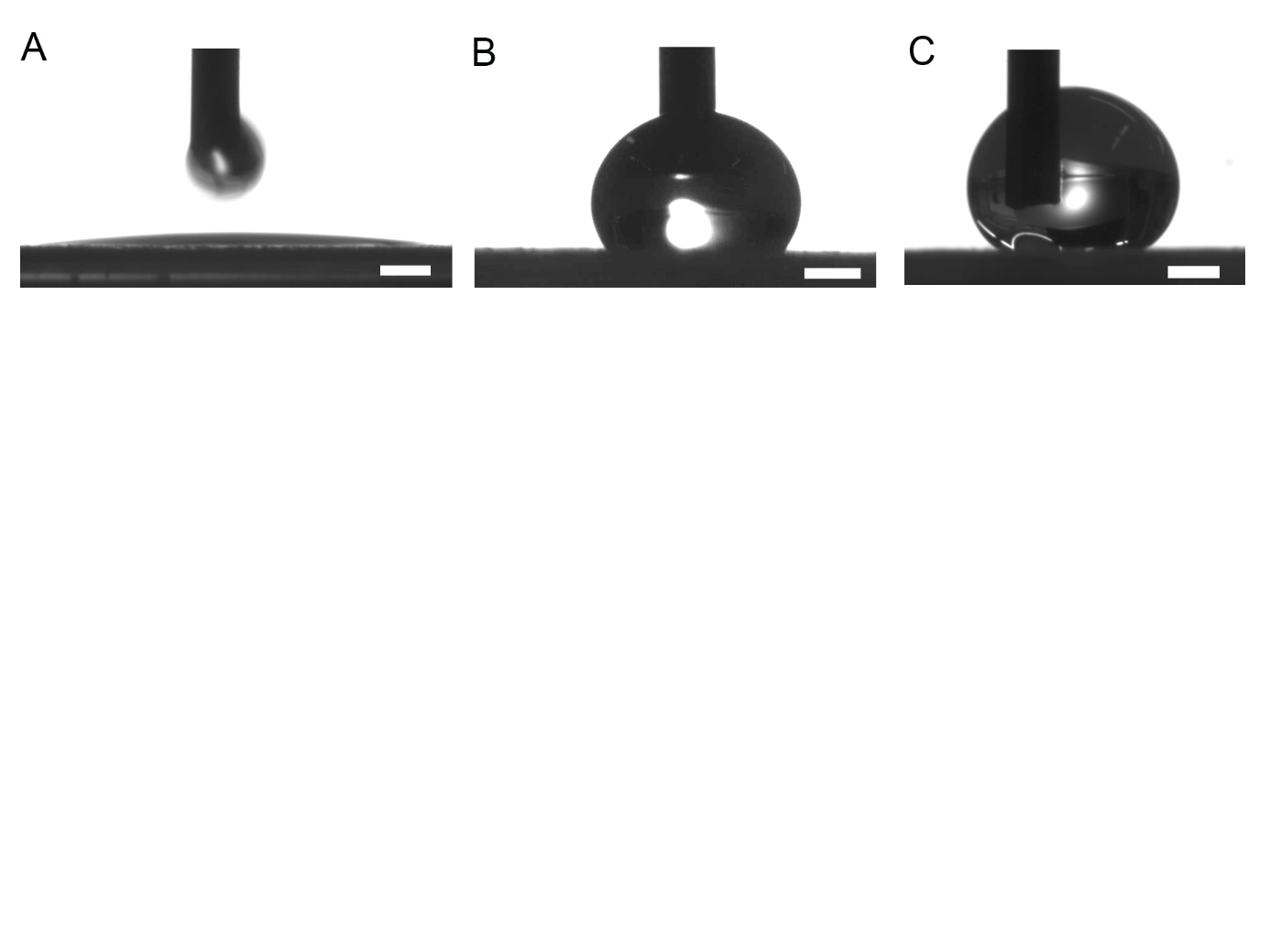


**Supplementary Figure S14.** Contact angles measured on (A) NH_2_-UiO-66 ≤10°, (B) A-UiO-66 = 122°±3°, and (C) F-UiO-66 = 135°±3°. Scale bars are 1 mm.


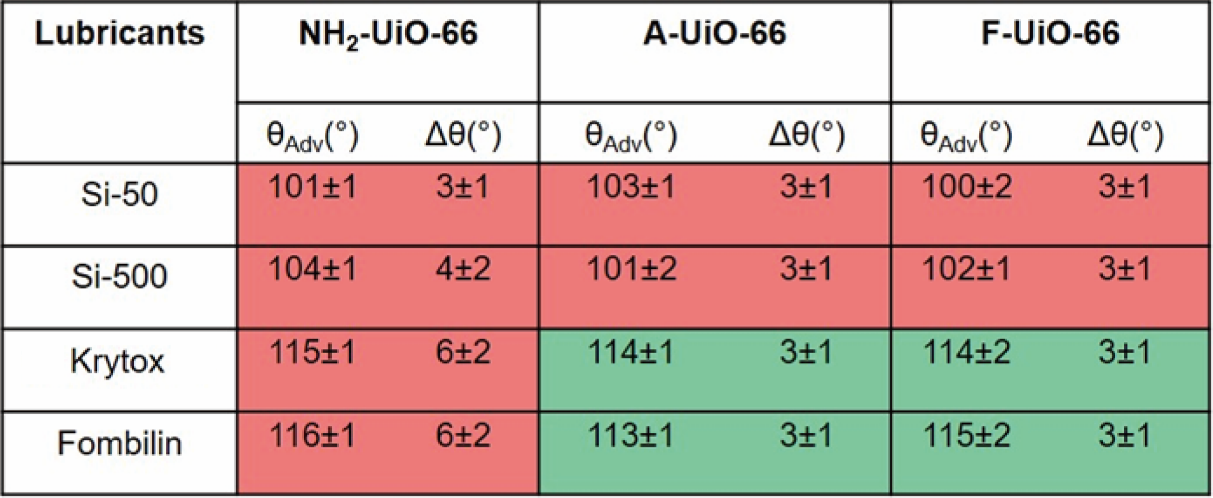


**Supplementary Figure S15.** The effect of pore surface chemistry on the SLIPS durability. Wettability data of hydrophilic (NH_2_-UiO-66) and hydrophobic (A-UiO-66 and F-UiO-66) MOFs infused with four different lubricants, Si50, Si500, Krytox, and Fomblin. Red and green colour represent the failure and stability of the surfaces under water shedding, respectively. Stability benchmark used was >10 water shedding cycles and Δθ ≤10°.

**
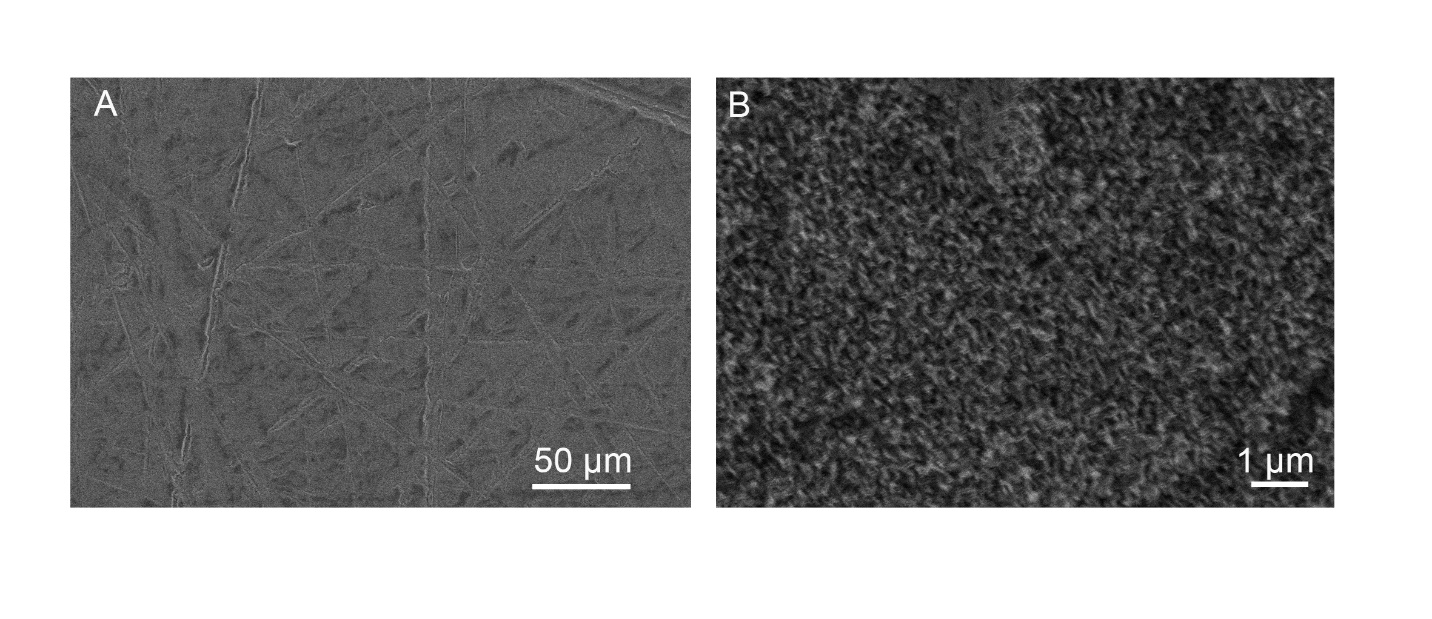
**

**Supplementary Figure S16.** SEM images of control boehmite sample (rough surface) prepared by etching in boiling water and imaged at different magnifications.


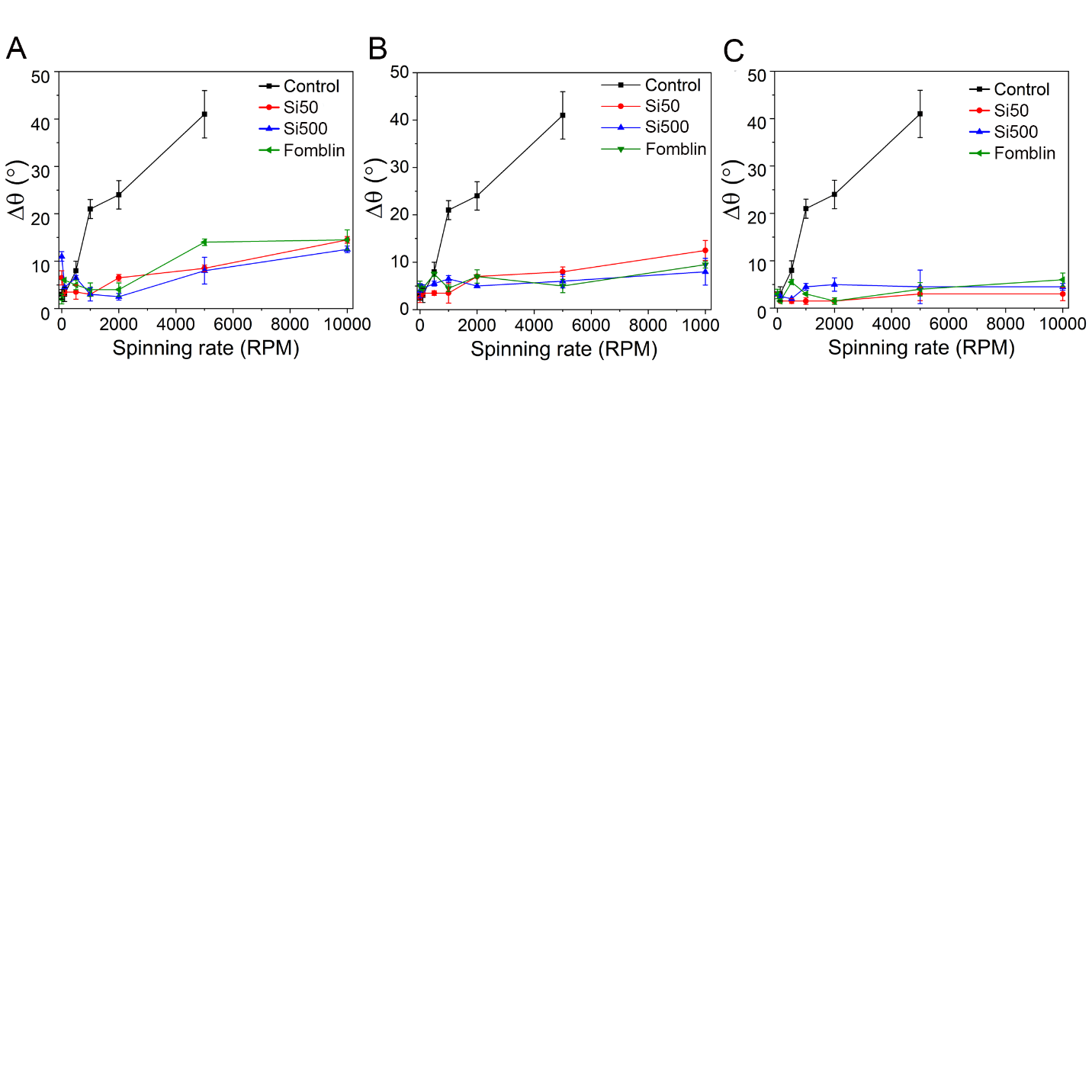


**Supplementary Figure** **S17.** Change in Δθ of various SLIPS with rotational speed: (A) NH_2_-UiO-66, (B) A-UiO-66, and (C) F-UiO-66. Spinning speeds ranged from 100 RPM to 10000 RPM.


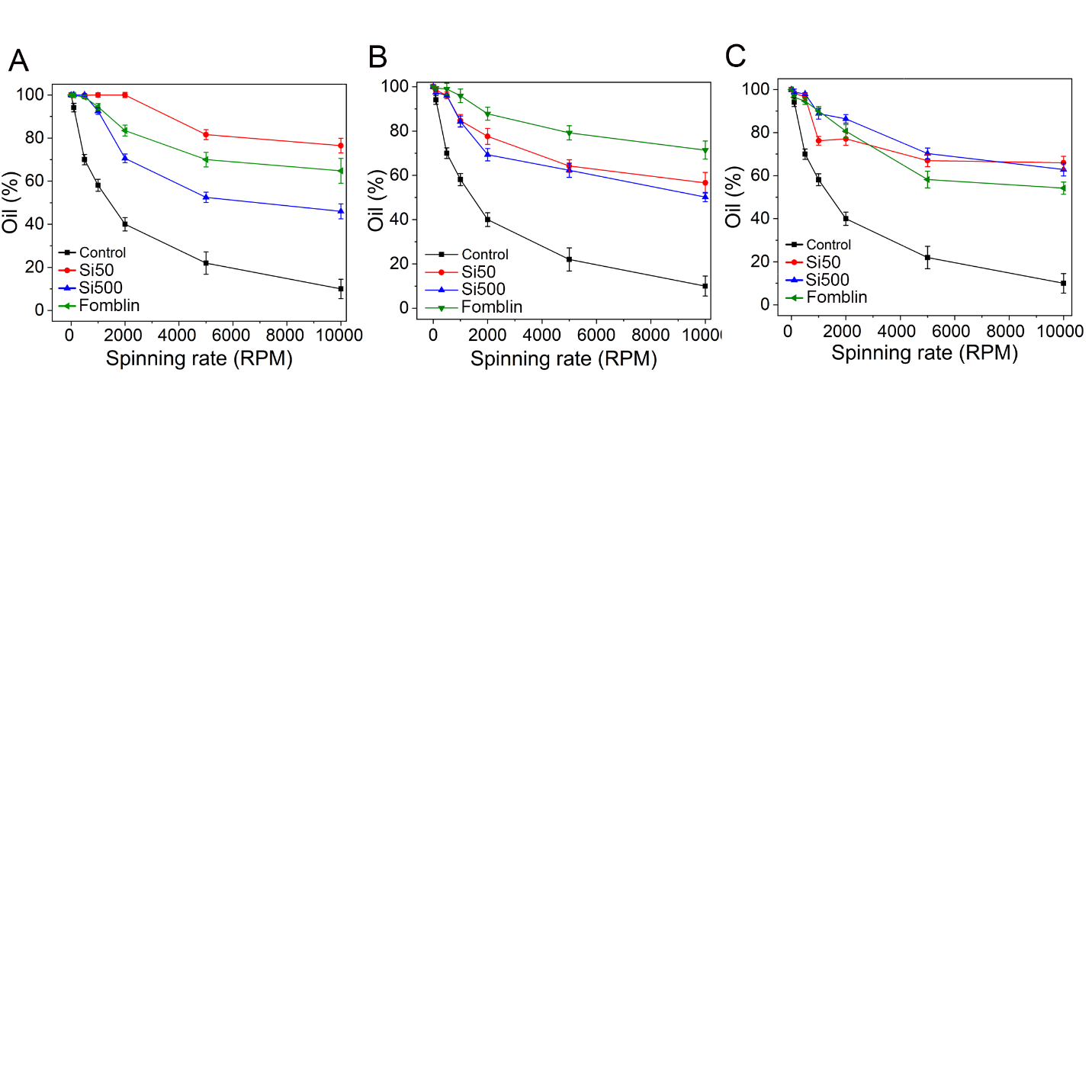


**Supplementary Figure S18.** Spinning stability data of MOF-based SLIPS showing percentage loss in lubricant weight for (A) NH_2_-UiO-66, (B) A-UiO-66, and (C) F-UiO-66.


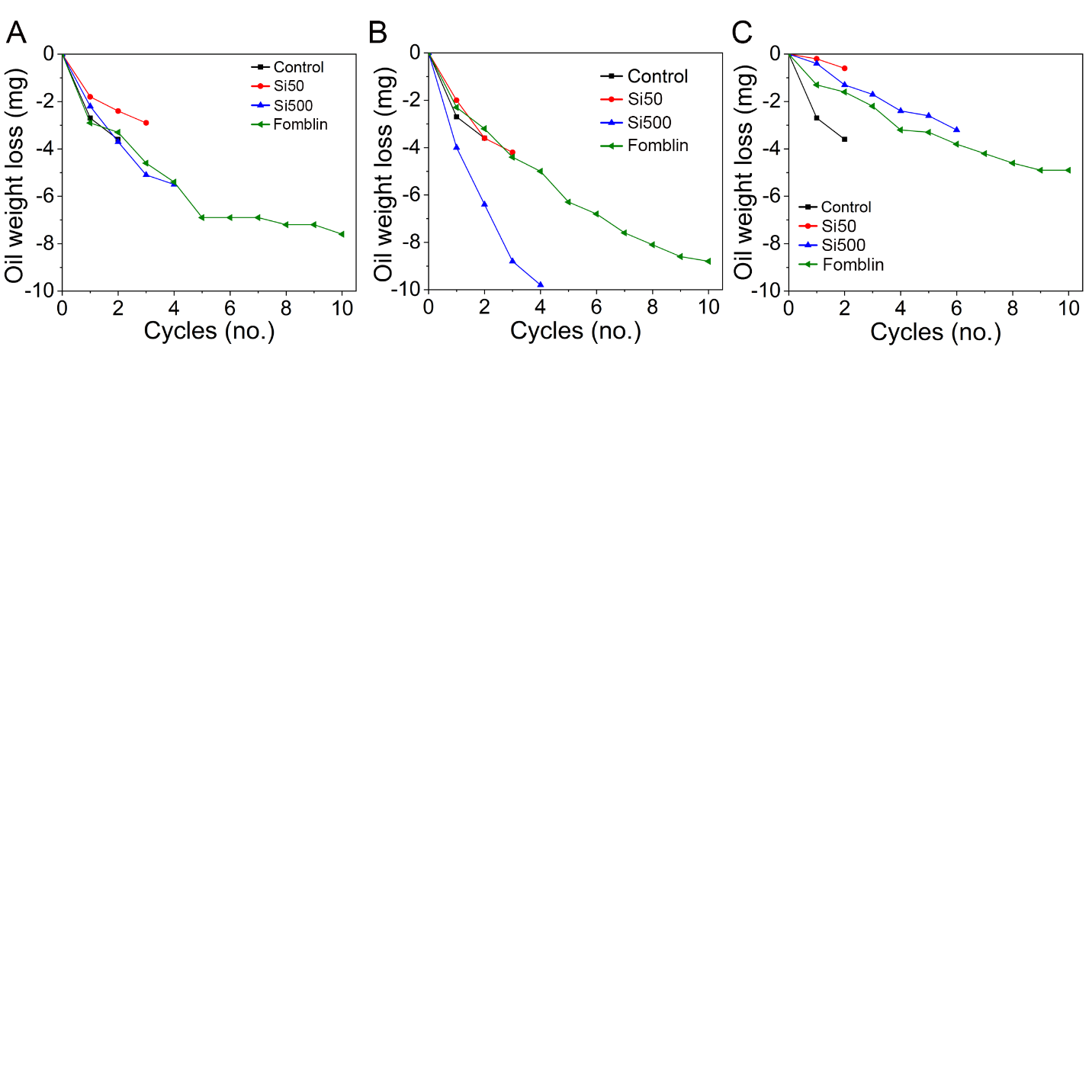


**Supplementary Figure S19.** Water shedding test on SLIPS. The loss of lubricants mass caused by depletion from (A) NH_2_-UiO-66, (B) A-UiO-66, and (C) F-UiO-66 recorded for 10 water shedding cycles. The volume of water in each cycle is 50 mL.


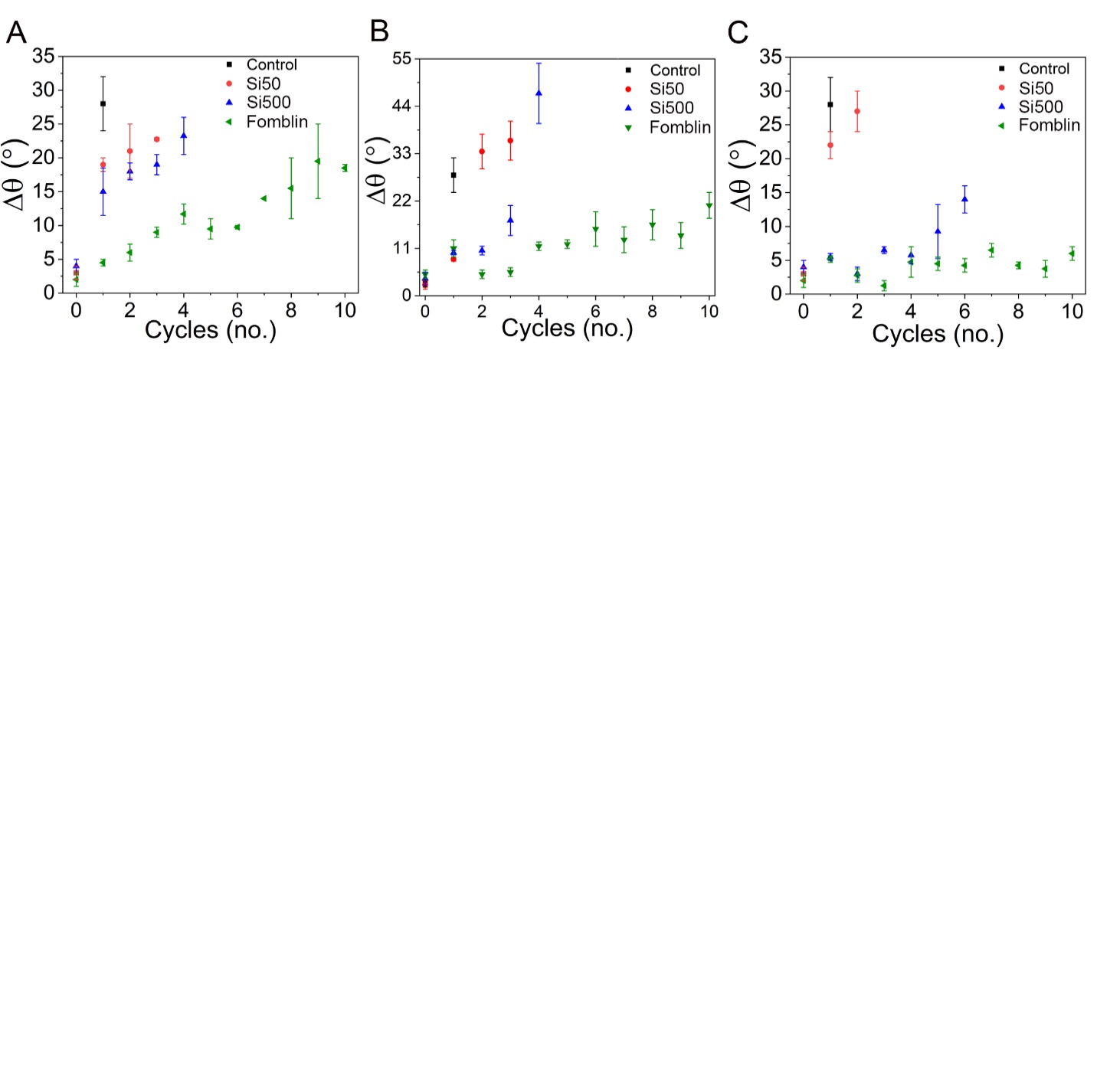


**Supplementary Figure S20.** The change in ∆θ caused by lubricant depletion from A-UiO-66 recorded for 10 water shedding cycles. The volume of water in each cycle is 50 mL.


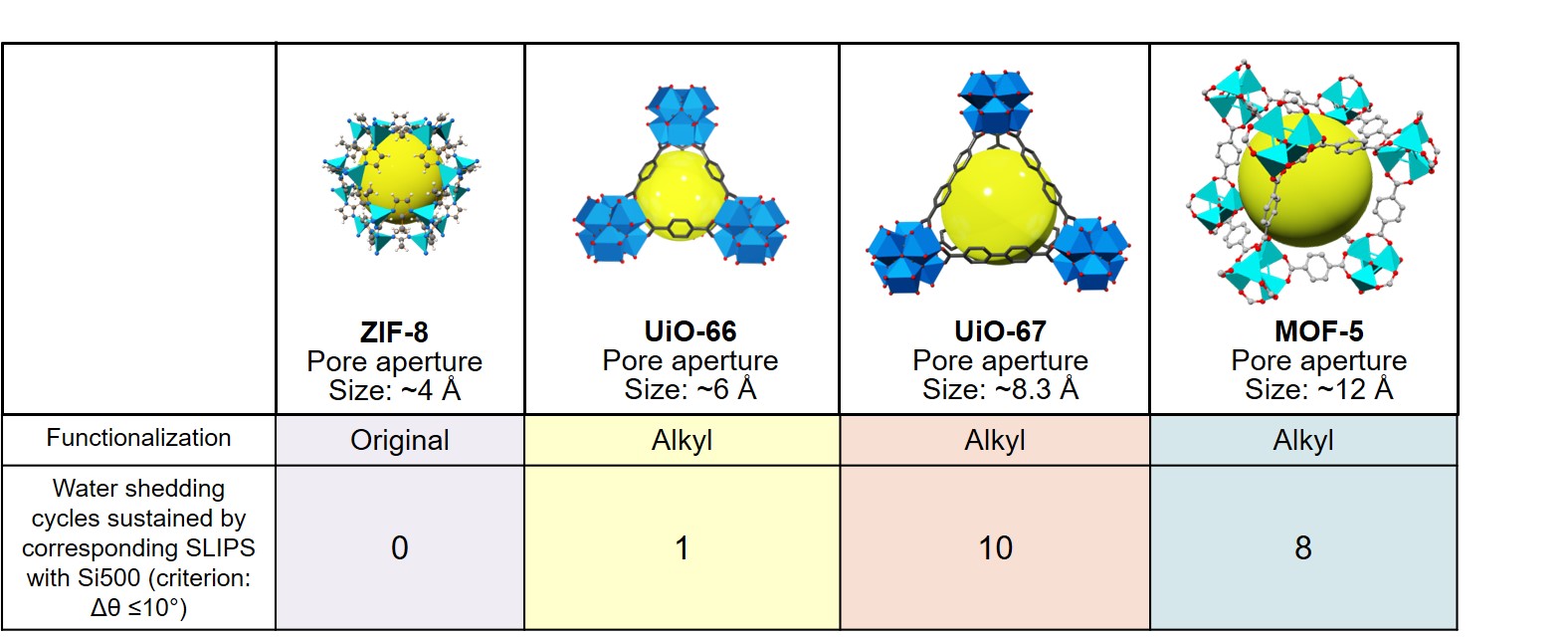


**Supplementary Figure S21.** Investigation of pore size from ~4Å - ~12Å on the durability of silicone (Si500) SLIPS. ZIF-8, UiO-66, UiO-67 and MOF-5 were selected from Figure 1J to study the effect of pore size on the SLIPS durability.


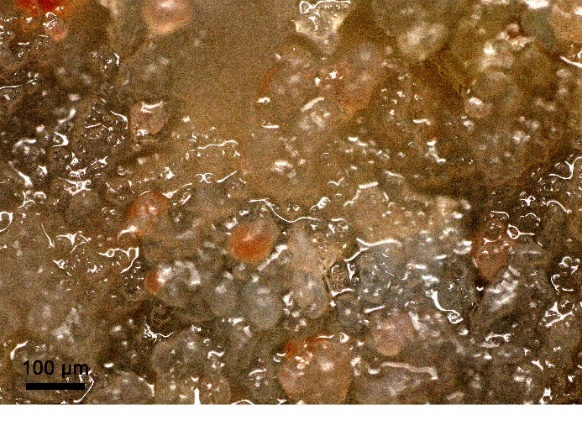


**Supplementary Figure S22.** Optical image of alkyl functionalised MOF-5 infused with Si500, showing large particles, > 50 µm in size.


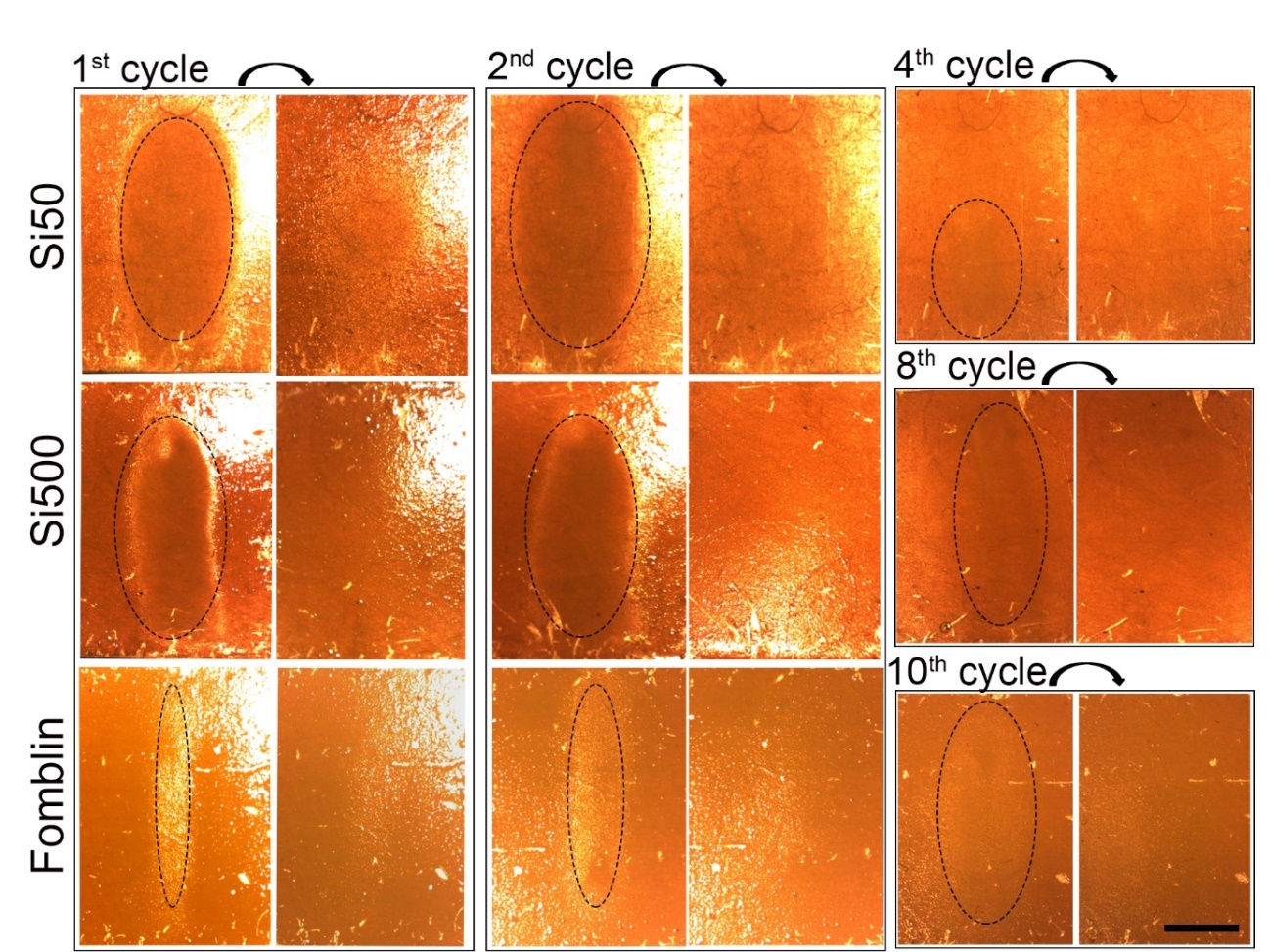


**Supplementary Figure S23.** Lubricant replenishment on F-UiO-66 SLIPS. The images are snapshots from videos, showing morphological changes before and after a given replenishment period. The average replenishment time for Si50, Si500 and Fomblin were 30 minutes, 180 minutes and 60 minutes, respectively. Surfaces infused with Si50 and Si500 were almost dried up after 3^rd^ and 7^th^ cycles. Depleted zones are marked with black dotted circles. Scale bars are 1 cm.





**Supplementary Figure S24.** FTIR spectra showing shift in C-F stretching in Fomblin after interacting with fluorine atoms in F-UiO-66, confirming strong intermolecular interactions. No shift was observed in other MOFs.


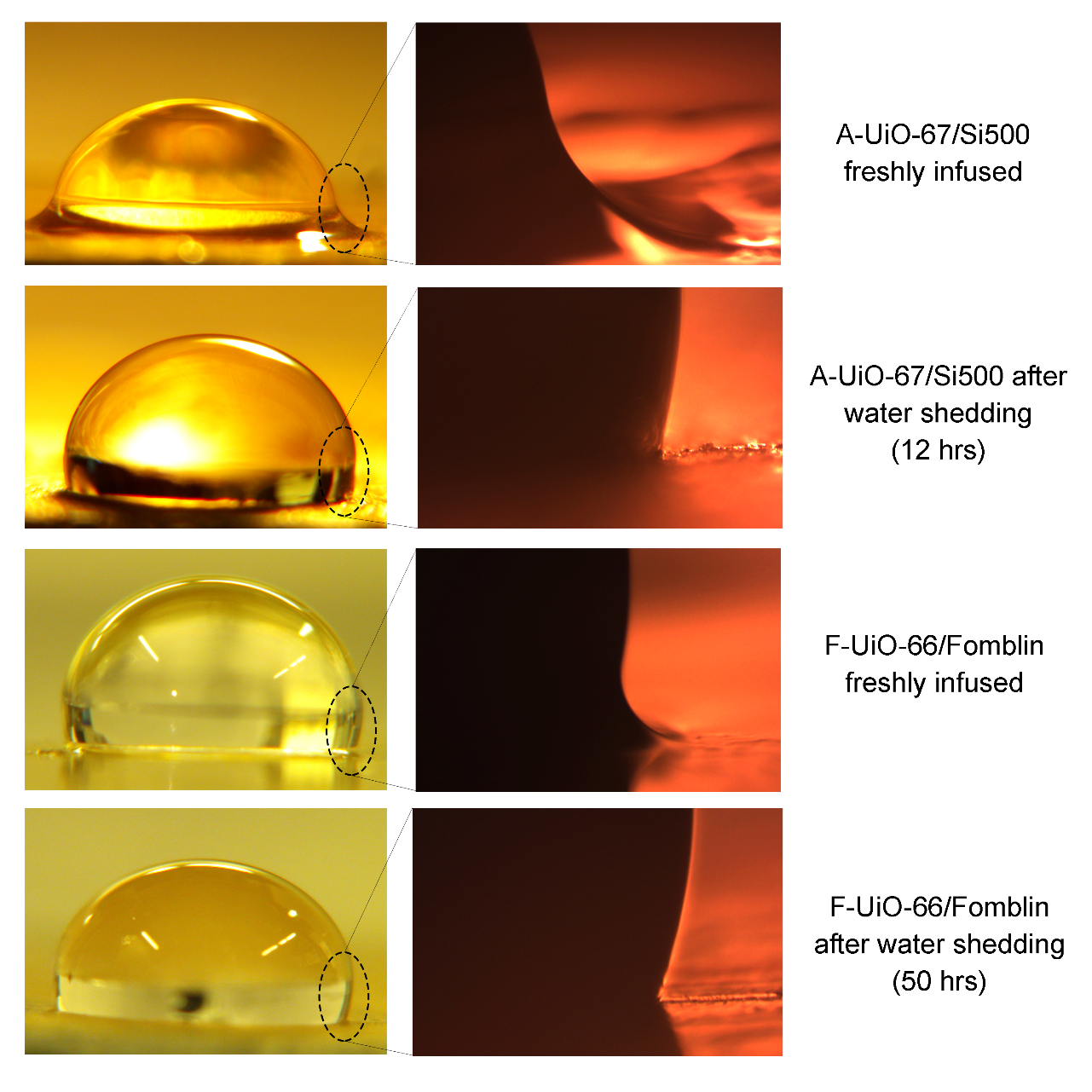


**Supplementary Figure S25.** Images from cloaking experiments showing wetting ridges on F-UiO-66/Fomblin and A-UiO-67/Si500 SLIPS before and after water shedding experiments. The water droplets were still able to slide on the surfaces albeit at slower speed.


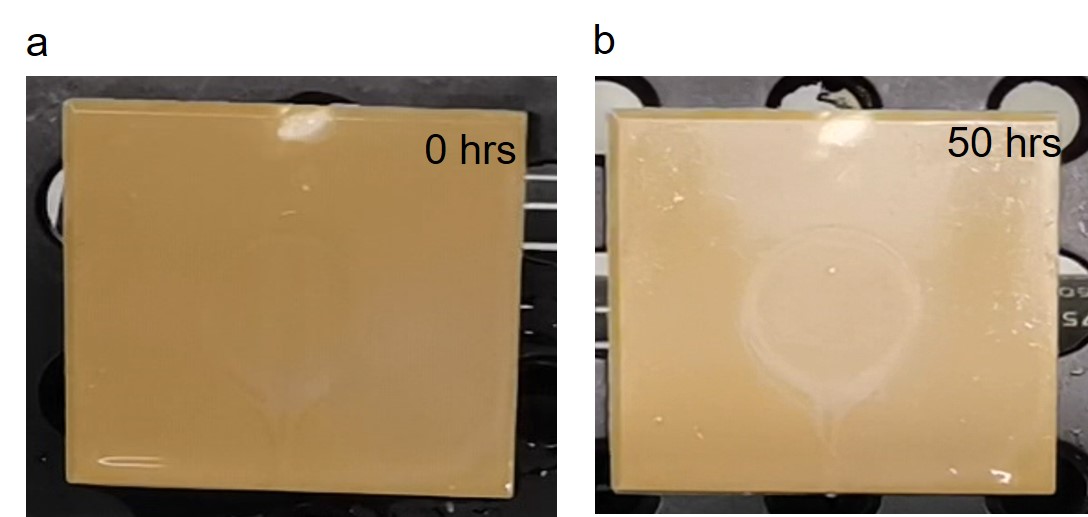


**Supplementary Figure S26.** **Long-term water shedding.** a) Fresh F-UiO-66/Fomblin SLIPS, and b) after 50 hrs of continuous water shedding. Lubricant depleted region is visible as faded spot in the middle of the sample. Movie showing slipperiness after 50 hrs is presented in Supplementary Movie 3.


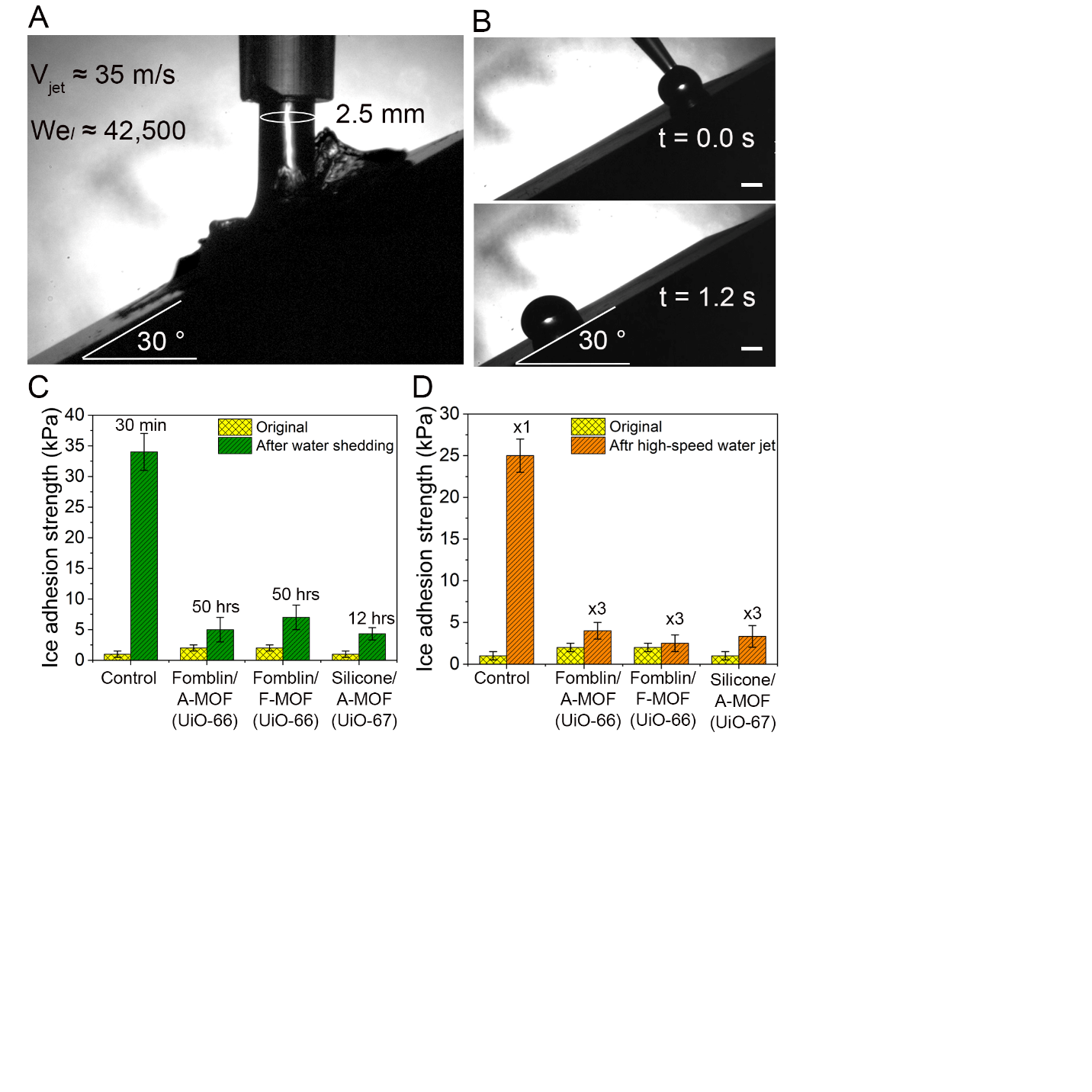


**Supplementary Figure S27.** Free sliding of water droplet after three repeated jet tests on F-UiO-66/Fomblin SLIPS. Scale bars are 1 mm.


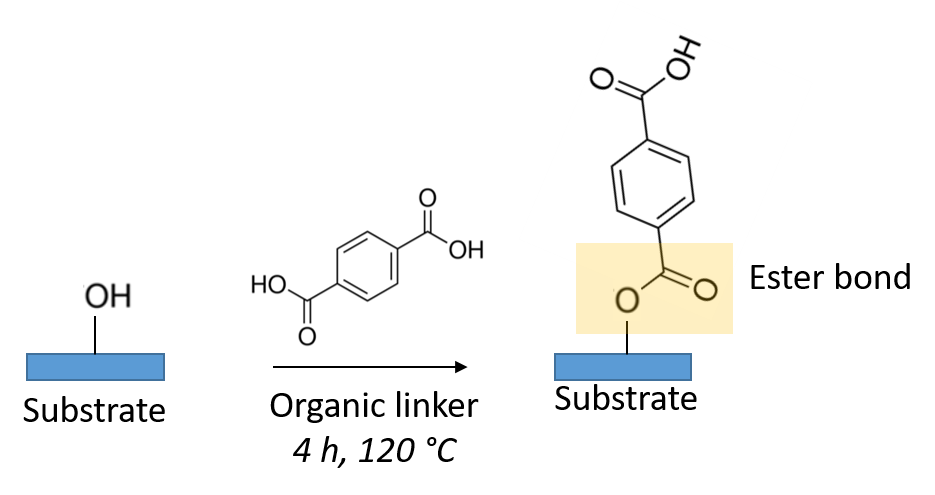


**Supplementary Figure S28.** Ester bond formation through condensation reaction between carboxylic acid and hydroxyl functional groups.


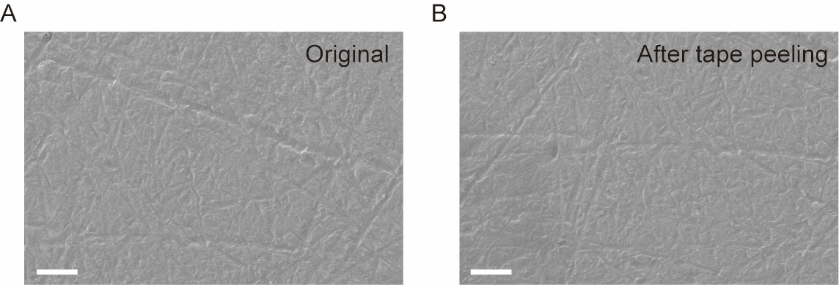


**Supplementary Figure S29.** SEM images showing the unchanged morphology on MOF-coated surface (A) before and (B) after tape peeling tests. Scale bar: 100 μm.


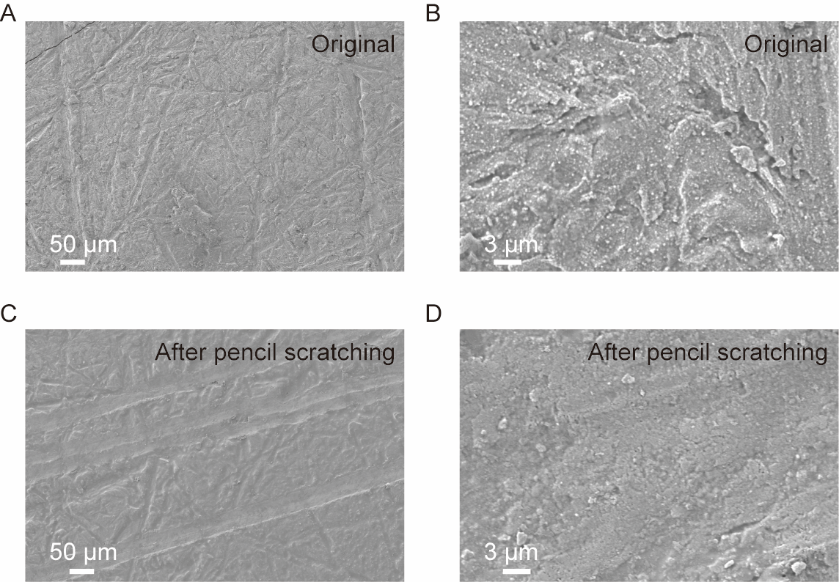


**Supplementary Figure S30.** SEM images showing the change of morphology on MOF-coated surface (A, B) before, and (C, D) after pencil scratch tests. The MOF materials remained intact on the substrates after the tests; however, the nanoscale rough structures on (C) the original MOF surfaces were compressed and flattened by pencil, as shown in D.


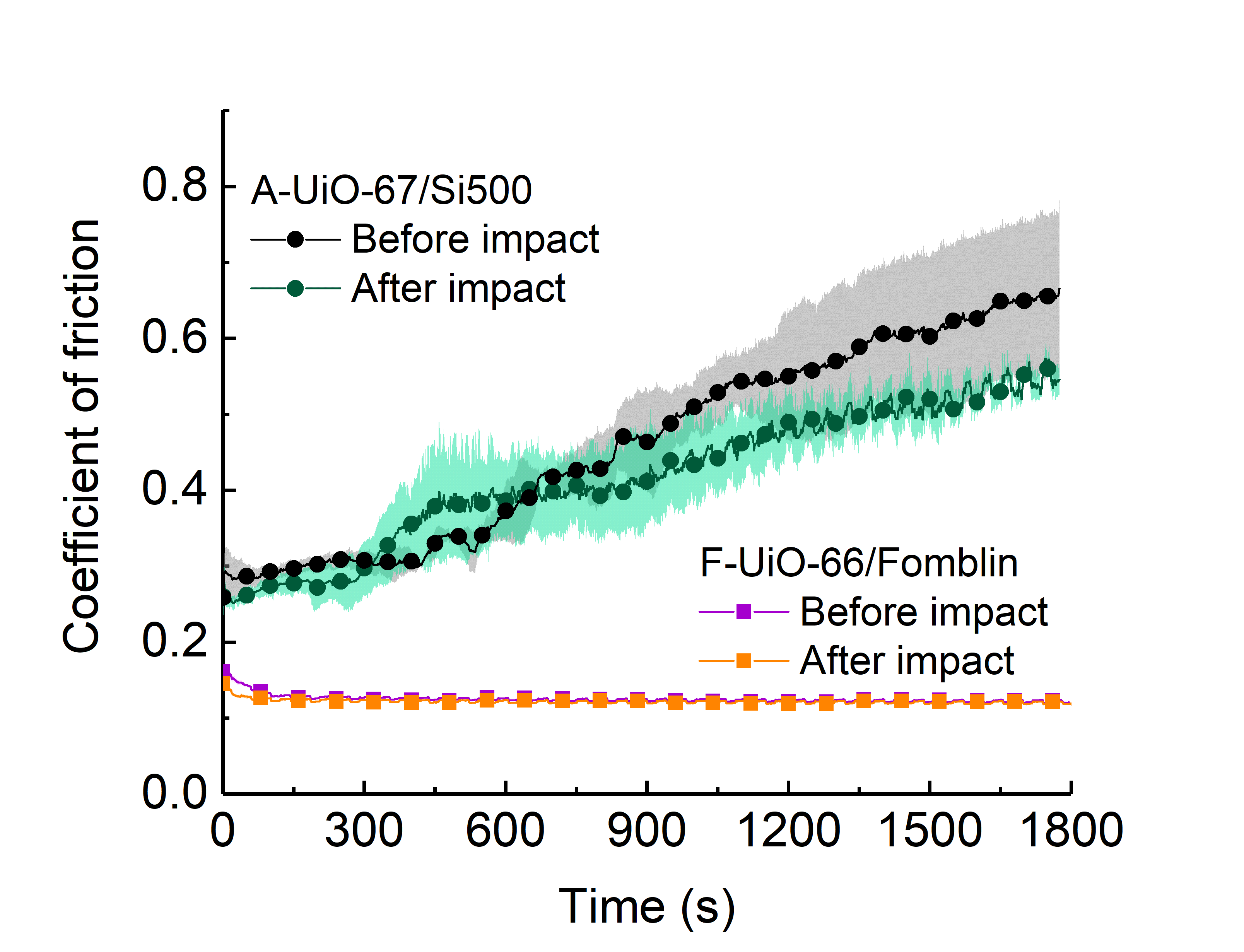


**Supplementary Figure S31.** **Friction coefficient after jet impact measured by a tribometer.** Two types of robust SLIPS, i.e. A-UiO-67/Si500 and F-UiO-66/Fomblin, were subjected to the tribological test before and after high-speed water jet impact.


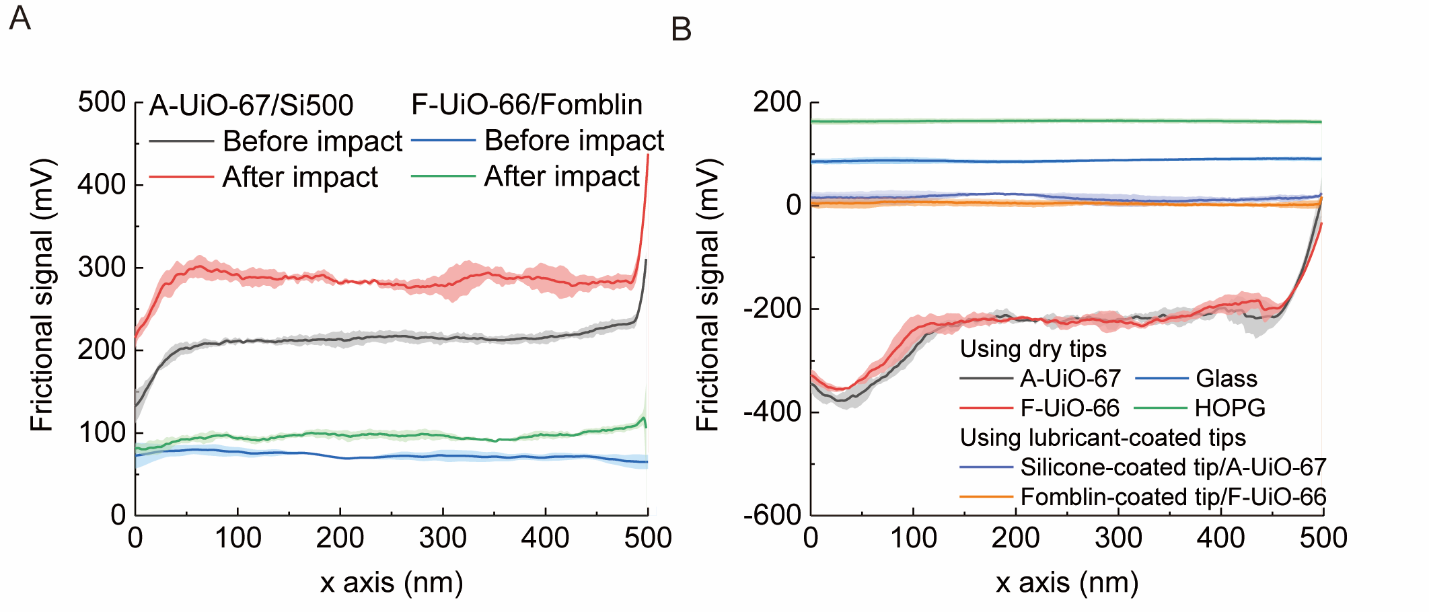


**Supplementary Figure S32.** **Nanotribological properties of surfaces measured by LFM in retrace scans.** (A) Frictional signal on SLIPS before and after high-speed water jet impact. (B) Frictional signal on different MOF surfaces using dry tips and lubricant-coated tips. Glass and highly oriented pyrolytic graphite (HOPG) were used as control. Averaged values were calculated from three different cross-sectional lines. The shade areas represent the standard error.


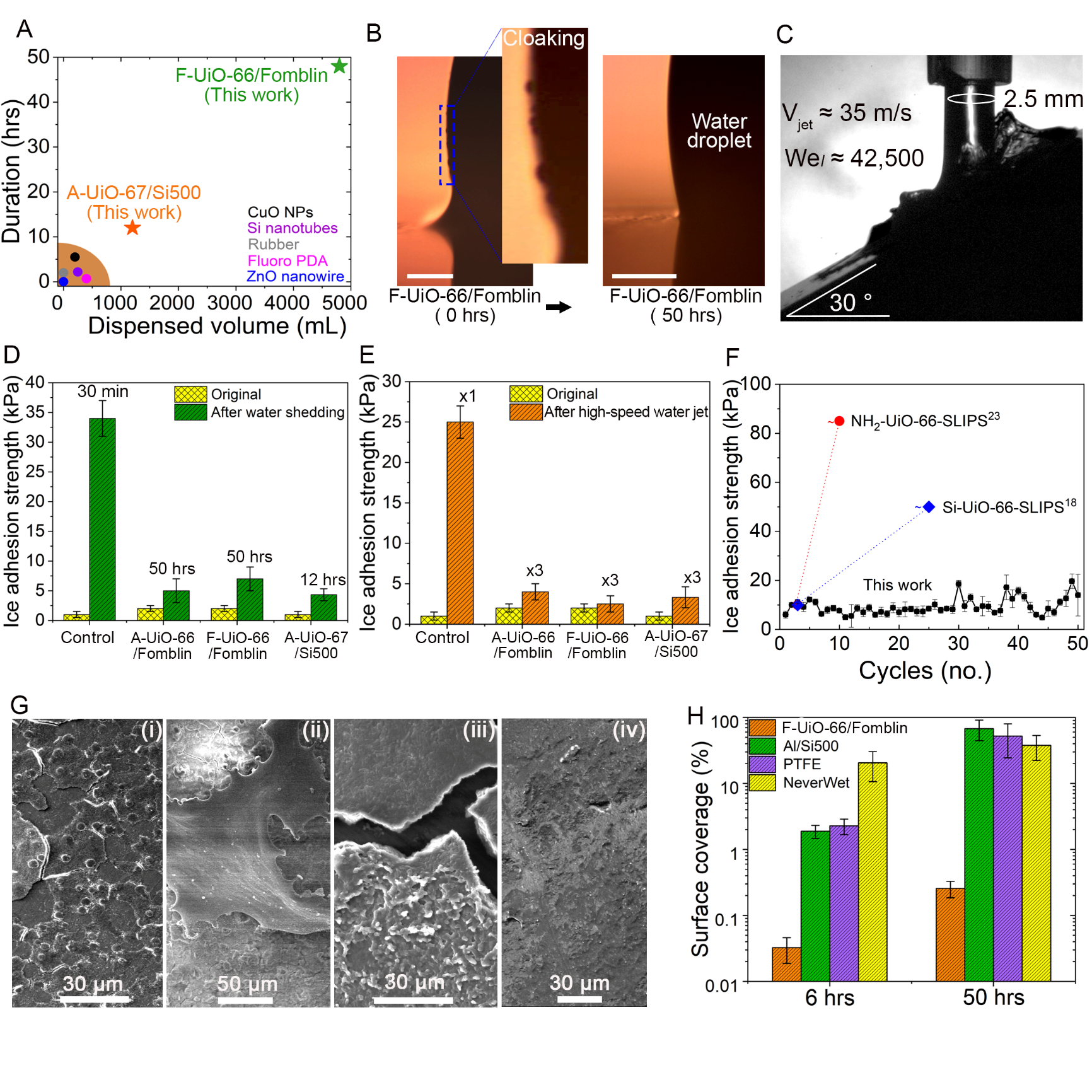


**Supplementary Figure S33.** Effect of continuous water shedding on ice adhesion strength on control surface (rough aluminium/Si500) and other MOF/lubricant combinations.


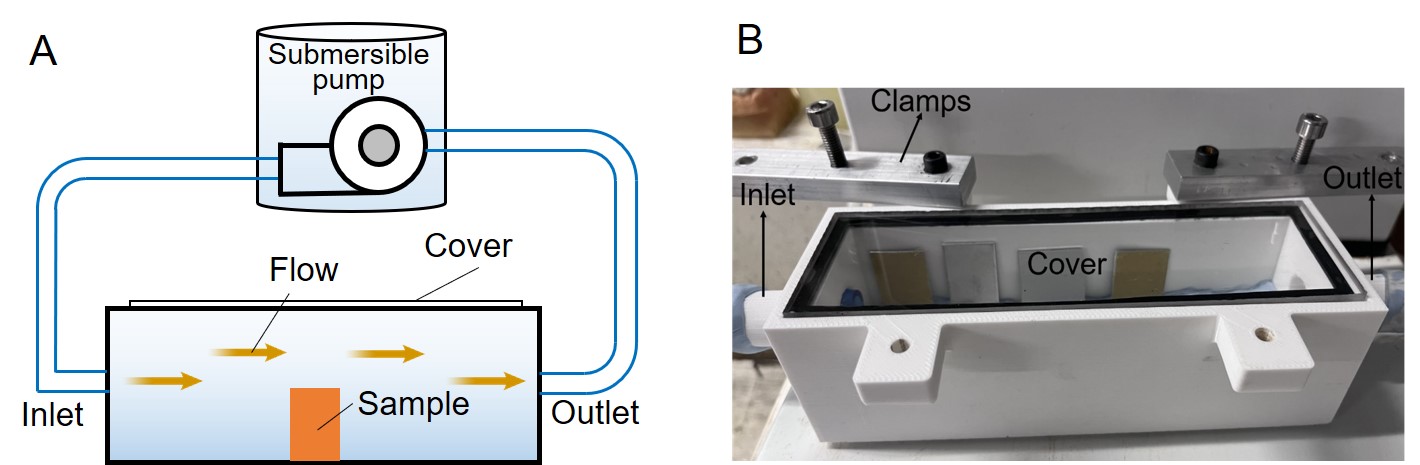


**Supplementary Figure S34**. (A) Schematic representation, and (B) photograph of closed-loop 3D printed chamber used for dynamic anti-fouling and anti-biofouling tests.


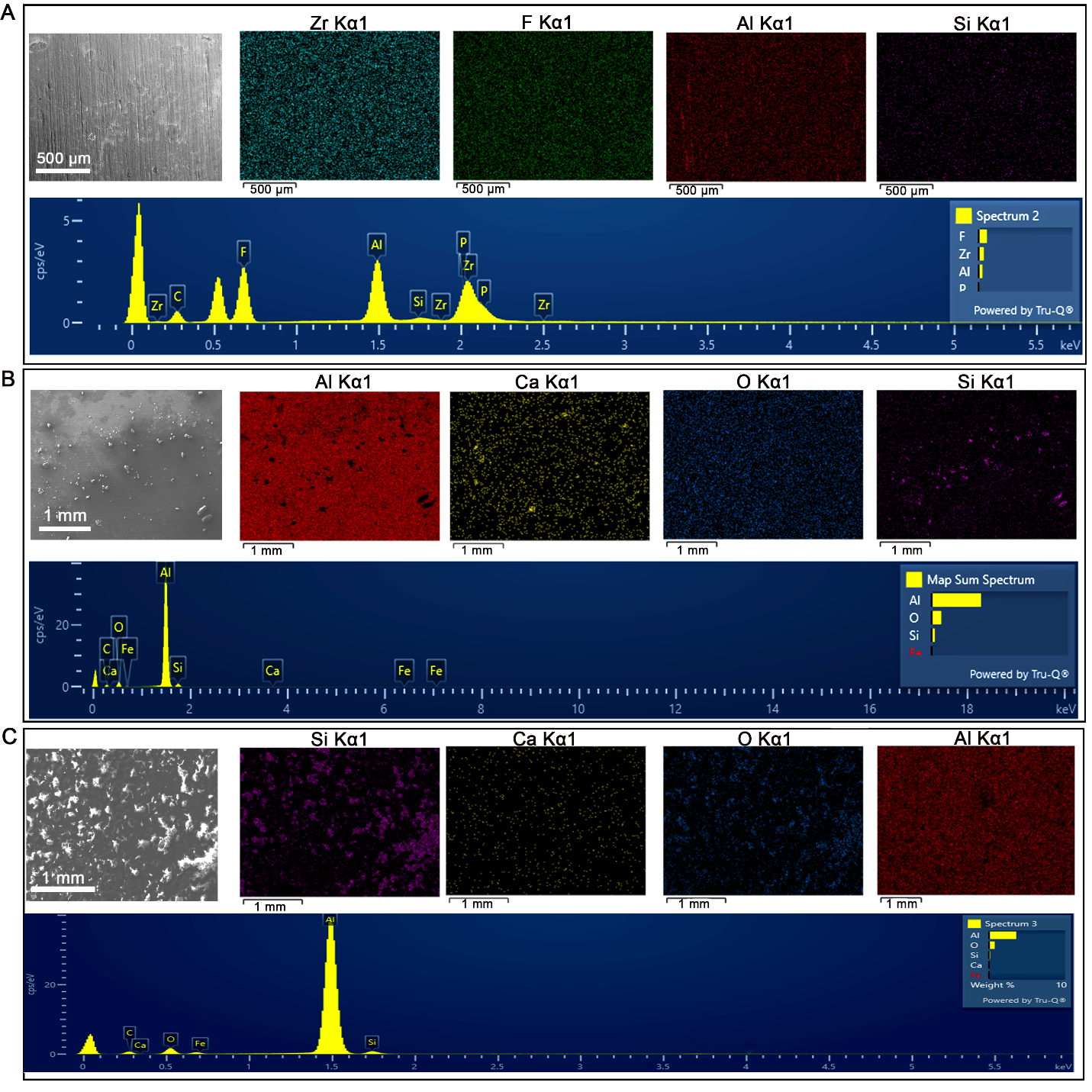


**Supplementary Figure S35.** SEM and EDS spectra of (A) F-UiO-66/Fomblin SLIPS, and two controls, including (B) Al/Si500 and (C) NewerWet®, subjected to dynamic anti-fouling tests for 96 hrs at 25 °C.


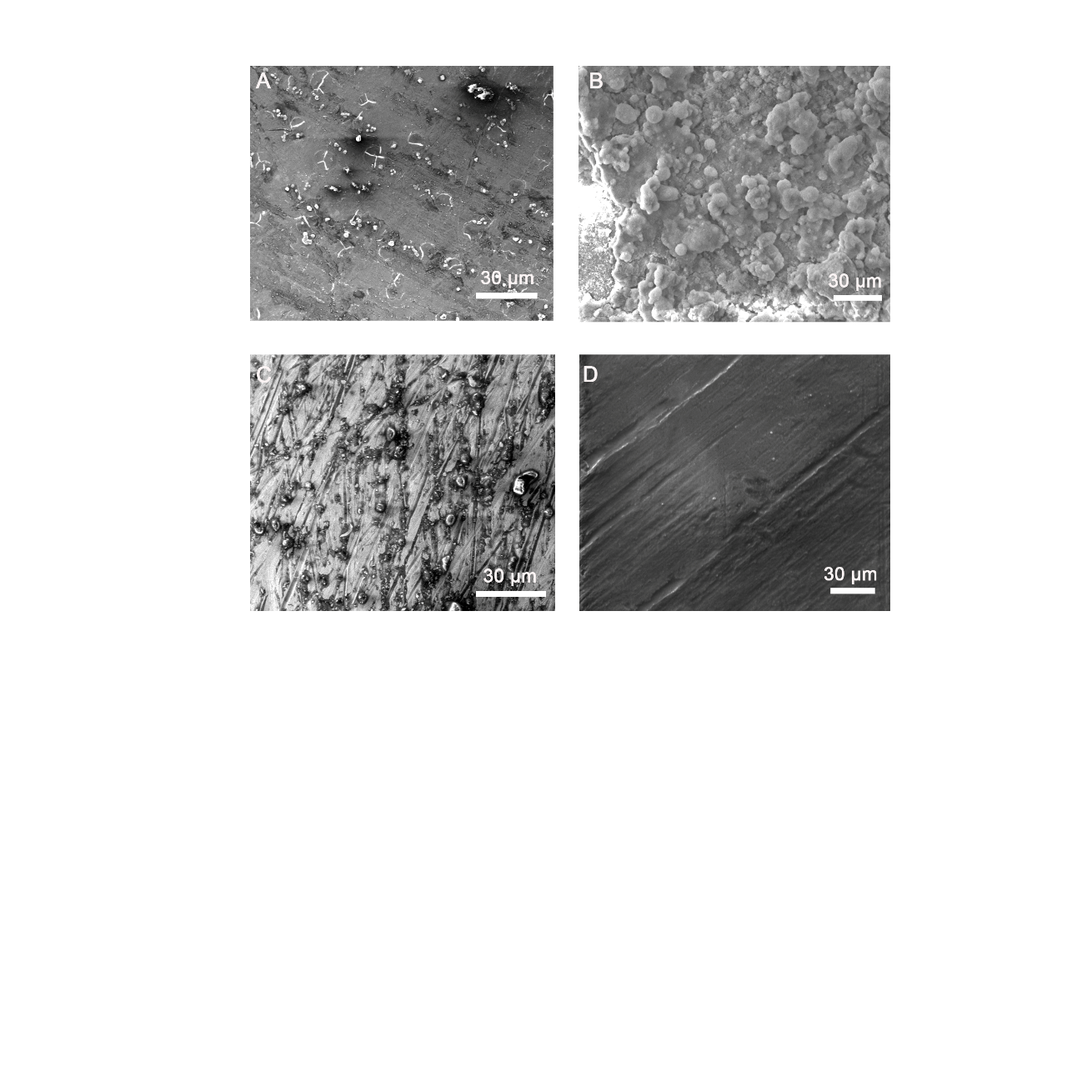


**Supplementary Figure S36.** SEM images of the (A) PTFE, (B) NeverWet®, (C) Al/Si500, and (D) F-UiO-66/Fomblin recorded after 6 hrs of incubation. Deposition of bacteria (S. aureus) could be readily observed on the control samples.

1. **Captions for supplementary movies**

**Supplementary movie 1:** The confinement of Fomblin chain inside the octahedral UiO-66 pore whereas silicone chain failed to enter.

**Supplementary movie 2:** Replenishment of different lubricants, Si50, Si500, and Fomblin from F-UiO-66. Black arrows are used to show the depletion dynamically.

**Supplementary movie 3**: Water shedding test, free sliding of water droplets at the beginning of the experiment (0 hrs) and after 50 hrs.

**Supplementary movie 4**: Assessment of high-speed jet impact resistance (V_jet_ = ~35 m/s, nozzle = 2.5 mm) on control surface (boehmite with Si500 lubricant), NH_2_-UiO-66, and F-UiO-66 SLIPS. Each surface was impacted at the same velocity of ~35 m/s followed by gentle placement of a 15 µL water droplet to assess the pinning. The free sliding of the water droplets off the surface confirming the retention of slippery behaviour of the F-UiO-66/Fomblin SLIPS.

**Supplementary movie 5:** High-speed water jet impacting (V_jet_ = ~35 m/s, nozzle = 2.5 mm) on alkyl functionalised A-UiO-67 infused with silicone oil and assessment of impact resistance using post-impact water drop sliding.

**Supplementary movie 6**: Ice adhesion test on F-UiO-66/Fomblin SLIPS recorded with cameras in parallel. The force unit for force gauge readings is Newton (N).

1. **References**

[1] I. Pakamorė, J. Rousseau, C. Rousseau, E. Monflier, P.Á. Szilágyi, *Green Chemistry* 2018, **20** 5292-5298.

[2] Y.-R. Lee, M.-S. Jang, H.-Y. Cho, H.-J. Kwon, S. Kim, W.-S. Ahn, *Chemical Engineering* *Journal* 2015, **271**, 276-280.

[3] S. Wang, Y. Lv, Y. Yao, H. Yu, G. Lu, *Inorganic Chemistry Communications* 2018, **93**, 56-60.

[4] H. Li, M. Eddaoudi, M. O'Keeffe, O.M. Yaghi, *Nature* 1999, **402**, 276-279.

[5] V. Singh, X. Men, M.K. Tiwari, *Nano Lett.* 2021, **21**, 3480-3486.

[6] P. Kim, M.J. Kreder, J. Alvarenga, J. Aizenberg, *Nano Lett*. 2013, **13**, 1793-1799.

[7] C. Peng, Z. Chen, M.K. Tiwari, *Nat Mater*. 2018, **17**, 355-360.

[8] J.D. Smith, R. Dhiman, S. Anand, E. Reza-Garduno, R.E. Cohen, G.H. McKinley, K.K. Varanasi, *Soft Matter*, 2013, **9**, 1772-1780.

[9] H. Liu, G. Cao, *Scientific Reports*, 2016, **6**, 23936.

[10] S. Plimpton, *Journal of Computational Physics*, 1995, **117**, 1-19.

[11] P.G. Boyd, S.M. Moosavi, M. Witman, B. Smit, *The Journal of Physical Chemistry Letters*, 2017, **8**, 357-363.

[12] B.H. Besler, K.M. Merz Jr., P.A. Kollman, *Journal of Computational Chemistry*, 1990, **11**, 431-439.

[13] S. Nosé, *The Journal of Chemical Physics*, 1984, **81**, 511-519.

[14] J.J. Wardzala, J.P. Ruffley, I. Goodenough, A.M. Schmidt, P.B. Shukla, X. Wei, A. Bagusetty, M. De Souza, P. Das, D.J. Thompson, C.J. Karwacki, C.E. Wilmer, E. Borguet, N.L. Rosi, J.K. Johnson, *The Journal of Physical Chemistry C*, 2020, **124**, 28469-28478.

[15] S. Shalel-Levanon, A. Marmur, *Journal of Colloid and Interface Science*, 2003, **262**, 489-499.

[16] P.E. Luner, E. Oh, *Colloids and Surfaces A: Physicochemical and Engineering Aspects,* 2001, **181**, 31-48.

[17] E.R. Johnson, S. Keinan, P. Mori-Sánchez, J. Contreras-García, A.J. Cohen, W. Yang, *Journal of the American Chemical Society*, 2010, **132**, 6498-6506.

[18] T. Lu, F. Chen, *Journal of Computational Chemistry*, 2012, **33**, 580-592.

[19] W. Humphrey, A. Dalke, K. Schulten, *Journal of Molecular Graphics*, 1996, **14**, 33-38.

[20] T. Lu, Q. Chen, J. Mol. Model. 2020, **26**, 315.

[21] Q. Xu, X. Zhao, *Journal of Materials Chemistry*, 2012, **22**, 16416-16421.

[22] S. Emamian, T. Lu, H. Kruse, H. Emamian, *Journal of Computational Chemistry*, **2019**, 40, 2868-2881.

[23] R.M. Parrish, L.A. Burns, D.G.A. Smith, A.C. Simmonett, A.E. DePrince, E.G. Hohenstein, U. Bozkaya, A.Y. Sokolov, R. Di Remigio, R.M. Richard, J.F. Gonthier, A.M. James, H.R. McAlexander, A. Kumar, M. Saitow, X. Wang, B.P. Pritchard, P. Verma, H.F. Schaefer, K. Patkowski, R.A. King, E.F. Valeev, F.A. Evangelista, J.M. Turney, T.D. Crawford, C.D. Sherrill, *Journal of Chemical Theory and Computation*, 2017, **13**, 3185-3197.

[24] G.B. Webber, G.W. Stevens, F. Grieser, R.R. Dagastine, D.Y.C. Chan, *Nanotechnology*, 2008, **19**, 105709.

[25] J. Cai, T. Jin, J. Kou, S. Zou, J. Xiao, Q. Meng, *Langmuir*, 2021, **37**, 1623-1636.

[26] K. Voïtchovsky, J.J. Kuna, S.A. Contera, E. Tosatti, F. Stellacci, *Nature Nanotechnology*, 2010, **5**, 401-405.

[27] J.J. Kuna, K. Voïtchovsky, C. Singh, H. Jiang, S. Mwenifumbo, P.K. Ghorai, M.M. Stevens, S.C. Glotzer, F. Stellacci, *Nature Materials*, 2009, **8**, 837-842.

[28] H.-J. Butt, P. Siedle, K. Seifert, K. Fendler, T. Seeger, E. Bamberg, A.L. Weisenhorn, K. Goldie, A. Engel, *Journal of Microscopy*, 1993, **169**, 75-84.

[29] S. Guriyanova, B. Semin, T.S. Rodrigues, H.J. Butt, E. Bonaccurso, *Microfluidics and Nanofluidics,* 2010, **8**, 653-663.

[30] H.-J. Butt, B. Cappella, M. Kappl, *Surface Science Reports*, 2005, **59**, 1-152.

[31] C.L. Luu, T.T.V. Nguyen, T. Nguyen, T.C. Hoang, *Advances in Natural Sciences: Nanoscience and Nanotechnology*, 2015, **6**, 025004.

[32] Q. Liu, Y. Yang, M. Huang, Y. Zhou, Y. Liu, X. Liang, *Applied Surface Science*, 2015, **346**, 68-76.

[33] H.-H. Tran, Y. Kim, C. Ternon, M. Langlet, D. Riassetto, D. Lee, *Advanced Materials Interfaces*, 2021, **8**, 2002058.

[34] S.K. Ujjain, P.K. Roy, S. Kumar, S. Singha, K. Khare, *Scientific Reports*, **2016**, 6, 35524.

[35] S.K. Laney, M. Michalska, T. Li, F.V. Ramirez, M. Portnoi, J. Oh, I.G. Thayne, I.P. Parkin, M.K. Tiwari, I. Papakonstantinou, *Langmuir*, 2021, **37**, 10071-10078.

[36] S. Li, F. Zhao, Y. Bai, Z. Ye, Z. Feng, X. Liu, S. Gao, X. Pang, M. Sun, J. Zhang, A. Dong, W. Wang, P. Huang, *Chemical Engineering Journal*, 2022, **431**, 133945.

[37] L. Qin, H. Yang, F.J. Mawignon, Y. Zhang, G. Dong, A facile method for fabricating super-slippery surface with long term and high-efficiency sustained release performance, *Progress in Organic Coatings*, 2023, **174**, 107275.

[38] J. Zhao, M. Chen, J. Liu, F. Yan, *Thin Solid Films*, 2009, **517**, 3752-3759.

[39] M. Grizen, T. Maitra, J.P. Bradley, M.K. Tiwari, *Heat Transfer Engineering*, 2020, **41**, 1663-1672.

[40] Y. Zhu, G. McHale, J. Dawson, S. Armstrong, G. Wells, R. Han, H. Liu, W. Vollmer, P. Stoodley, N. Jakubovics, J. Chen, A*CS Applied Materials & Interfaces*, 2022, **14**, 6307-6319.

[41] H. Zhao, S. Khodakarami, C.A. Deshpande, J. Ma, Q. Wu, S. Sett, N. Miljkovic, A*CS Applied Materials & Interfaces*, 2021, **13**, 38666-38679.

[42] M.H. Chaudhry, *Applied Hydraulic Transients, Springer New York, New York, NY,* 2014, pp. 35-64.
